# Solvent-Modulated Specific Ion Effects: Poly(*N*-isopropylacrylamide) Brushes in Nonaqueous Electrolytes

**DOI:** 10.1021/acs.langmuir.3c02596

**Published:** 2023-12-20

**Authors:** Hayden Robertson, Isaac J. Gresham, Andrew R. J. Nelson, Kasimir P. Gregory, Edwin C. Johnson, Joshua D. Willott, Stuart W. Prescott, Grant B. Webber, Erica J. Wanless

**Affiliations:** † College of Science, Engineering and Environment, University of Newcastle, Callaghan, New South Wales 2308, Australia; ‡ School of Chemistry, University of Sydney, Sydney 2052, Australia; § Australian Centre for Neutron Scattering, ANSTO, Locked Bag 2001, Kirrawee DC, New South Wales 2232, Australia; ∥ Division of Biomedical Science and Biochemistry, Research School of Biology, The Australian National University, Canberra, Australian Capital Territory 0200, Australia; ⊥ Department of Chemistry, University of Sheffield, Dainton Building, Brook Hill, Sheffield S3 7HF, U.K.; # School of Chemical Engineering, UNSW Sydney, Sydney, New South Wales 2052, Australia

## Abstract

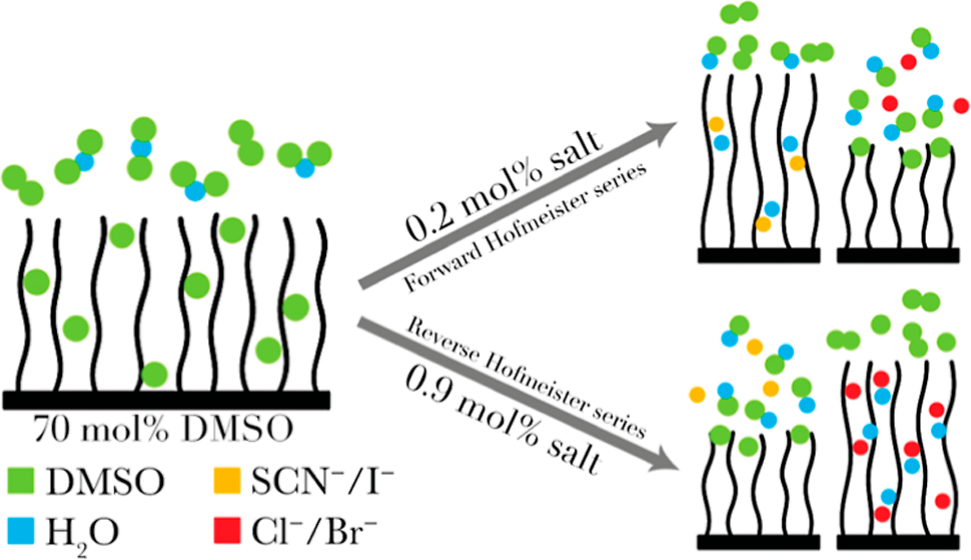

Pertinent to cryopreservation as well as energy storage and batteries, nonaqueous electrolytes and their mixtures with water were investigated. In particular, specific ion-induced effects on the modulation of a poly(*N*-isopropylacrylamide) (PNIPAM) brush were investigated in various dimethyl sulfoxide (DMSO)–water solvent mixtures. Spectroscopic ellipsometry and neutron reflectometry were employed to probe changes in brush swelling and structure, respectively. In water-rich solvents (i.e., pure water and 6 mol % DMSO), PNIPAM undergoes a swollen to collapsed thermotransition with increasing temperature, whereby a forward Hofmeister series was noted; K^+^ and Li^+^ electrolytes composed of SCN^–^ and I^–^ salted-in (stabilized) PNIPAM chains, and electrolytes of Cl^–^ and Br^–^ salted-out (destabilized) the polymer. The cation was seen to play a lesser role than that of the anion, merely modulating the magnitude of the anion effect. In 70 mol % DMSO, a collapsed to swollen thermotransition was noted for PNIPAM. Here, concentration-dependent specific ion effects were observed; a forward series was observed in 0.2 mol % electrolytes, whereas increasing the electrolyte concentration to 0.9 mol % led to a series reversal. While no thermotransition was observed in pure DMSO, a solvent-induced specific ion series reversal was noted; SCN^–^ destabilized the brush and Cl^–^ stabilized the brush. Both series reversals are attributed to the delicate balance of interactions between the solvent, solute (ion), and substrate (brush). Namely, the stability of the solvent clusters was hypothesized to drive polymer solvation.

## Introduction

Polymer brushes are thin films that consist of surface-tethered polymer chains with a high grafting density. Depending on the polymer identity, the physicochemical properties of brushes are capable of responding to changes in temperature,^1−3^ pH,^4,5^ solvent composition,^6^ as well as electrolyte concentration and identity.^7−13^ The presence of these brush coatings modifies interfacial properties such as surface charge, polymer conformation, lubricity, and adhesion: establishing a strong basis for smart interfaces.^14−17^ In particular, thermoresponsive polymers such as poly(*N*-isopropylacrylamide) (PNIPAM) or poly(oligo(ethylene glycol) methyl ether methacrylates) (POEGMAs) are well-studied for their temperature-induced phase changes around a critical solution temperature (CST). For free PNIPAM in aqueous solution, this is a lower CST (LCST): a solvated to desolvated transition with increasing temperature.^1^ For thermoresponsive polymer brushes, this translates to a swollen to collapsed transition with increasing temperature. Thermoresponsive polymer brushes have a strong history as exemplar systems for interrogating various physicochemical phenomena as they have the potential to provide information about the system regardless of polymer solvation;^7−9,18^ the structure of a polymer brush can be examined in both “good” and “poor” solvents.^6,14,16^ This is not the case for ungrafted polymers, gels, and polymers grafted to particles due to inevitable colloidal instability.

**Figure 1:**
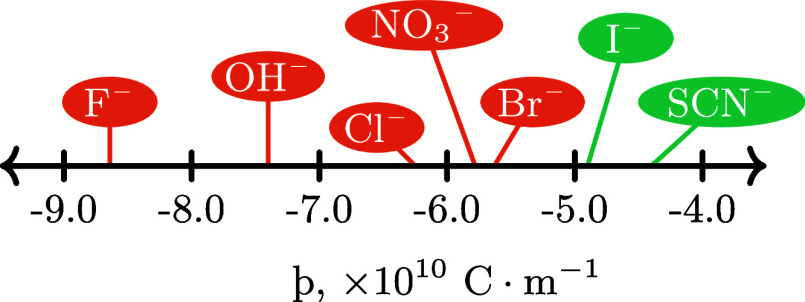
þ (“sho”) values for a subset of anions across the Hofmeister series. Orange indicates classically salting-out anions and green indicates salting-in anions. The anions investigated span a broad range of þ values while retaining the simplest structures (mostly monatomic, all monovalent). þ values are calculated in a vacuum and are agnostic to solvent composition.

Specific ion effects (SIE) pertain to any electrolyte-imparted phenomenon dependent on ion identity, not just charge or concentration.^19^ These effects are ubiquitous in nature and industry, governing a wide range of systems: from the stability of colloidal dispersions^20,21^ and proteins,^22^ to solution^23^ and interfacial properties,^24^ as well as polymer solubility.^7−9,18^ First reported by Franz Hofmeister, one particular subset of SIE is the Hofmeister series, which orders ions based on their ability to stabilize (salt-in) or destabilize (salt-out) egg-white globulin in aqueous solution.^25^ Historically, salting-in and salting-out ions have been referred to as chaotropic and kosmotropic ions, respectively, due to their hypothesized structure-making and breaking influence on water.^19,26−28^ However, recent computational advances comparing SIE in the absence of solvent (i.e., vacuum) to the presence of solvent have revealed that SIE cannot be universally understood by considering the effect of the ion on solvent structure alone; rather, the many interactions between the solvent(s), solute (i.e., ion), and substrate (e.g., polymer) must be considered.^29,30^ Gregory et al. have recently quantified ion specificity in terms of þ (“sho”), an interaction-site-specific ion radial charge density parameter that is calculated in vacuum, and is hence solvent independent.^30^ Anions that impart a salting-out effect on macromolecules in aqueous solution typically possess a more negative þ value than those that salt-in macromolecules in aqueous solution. An illustration of the relative magnitude of þ values^30^ for various anions in the Hofmeister series is presented in Figure 1.

Previous investigations of PNIPAM and polymers from the POEGMA family (both grafted and ungrafted) in aqueous solution have demonstrated that ions on the right of the Hofmeister series, such as SCN^–^ and I^–^, salt-in these polymers, increasing the LCST and polymer solubility. Conversely, anions on the left of the series, such as Cl^–^ and Br^–^, decrease the LCST and salt-out these polymers.^8,19,31,32^ Zhang et al. have previously proposed three mechanisms to describe these phenomena.^31^ In short, the authors suggest that all anions act to destabilize the polymer (i.e., salt-out) via polarization of the hydrogen bond between the polymer and solvent, as well as increasing the surface tension around the hydrophobic moieties on the polymer. Additionally, more hydrophobic and charge diffuse anions, such as SCN^–^ and I^–^, override the aforementioned salting-out effects by binding to the polymer chains, yielding a net salting-in effect. While aqueous SIE have been well-studied both experimentally and computationally, the manifestation of SIE in nonaqueous solvents and their mixtures with water have not received such attention to date. However, the work of Mazzini and Craig demonstrated that SIE occur in many solvents irrespective of their hydrogen-bonding capacity,^33,34^ ruling out one of the causal phenomena proposed by Zhang et al.^31^ Further improving our understanding of ion-specific behavior in nonaqueous electrolytes and their mixtures with water is a step toward unraveling ion specificity in natural environments, and is integral to establishing a universal predictive theory of SIE.

DMSO is a polar aprotic solvent that has sparked particular academic interest due to its interesting nonideal mixing behavior, as well as its industrial use as a cryopreservant and being a significant byproduct in the wood pulping industry.^35−39^ In a precursor study, we investigated the modulation of the PNIPAM brush thermoresponse and polymer conformation as a function of various DMSO–water solvent compositions across 5 to 55 °C.^6^ Pure DMSO is a “good” solvent for PNIPAM, such that PNIPAM exhibits no thermoresponse when exposed to the pure solvent within the temperature range studied.^40,41^ However, both spectroscopic ellipsometry and neutron reflectometry (NR) confirmed a cononsolvency region, whereby the mixture of these two “good” solvents (water and DMSO) resulted in a “poor” solvent regime for PNIPAM brushes. In water-rich regimes, small additions of DMSO resulted in a decrease in the LCST of PNIPAM, up to a DMSO mole fraction (*x*_D_) of approximately 0.10: a salting-out effect caused by a chaotropic cosolvent. A cononsolvency regime (i.e., a collapsed PNIPAM region) was then noted for 0.20 ≤ *x*_D_ ≤ 0.60, with slight re-entrant swelling across this region with increasing *x*_D_. Here, favorable DMSO–water interactions prevail, forming solvent clusters that inhibit polymer solvation; water molecules will preferentially form DMSO–water clusters over PNIPAM–water interactions.^6^ At *x*_D_ = 0.70, an upper CST (UCST) was noted: a collapsed to swollen phase transition as the hydrogen bonds between DMSO–water aggregates rupture with increasing temperature. These DMSO–water clusters face entropic limitations and are unable to penetrate deep into the brush; the remaining DMSO molecules form nonsite-specific interactions (DMSO–PNIPAM and DMSO–DMSO) and govern polymer solvation. The UCST behavior exhibited by PNIPAM in *x*_D_ = 0.70 was attributed to the thermal disruption of DMSO–water hydrogen bonds, rupturing solvent aggregates, and increasing polymer solvation with increasing temperature.^6^

Two recent studies have investigated SIE in both pure DMSO and DMSO-rich solvent compositions for ungrafted PNIPAM^42^ and polymer-supported boronic acid (PBA).^43^ The authors noted that all anions, regardless of their identity (i.e., þ value), imparted their effects in the same direction. Conversely, investigations by Mazzini and Craig have noted a Hofmeister series reversal in a (poly(2-(methacryloyloxy)ethyl)trimethylammonium)chloride (PMETAC) brush solvated by pure DMSO.^33,34^ These studies on both neutral and charged polymers lack a fundamental explanation of the mechanism behind the manifestation of SIE in DMSO and DMSO–water binary solvents.

Herein, we probe SIE in various DMSO–water binary solvents using a PNIPAM brush exemplar. Spectroscopic ellipsometry is employed to investigate changes in brush thickness and thermoresponse. Specular NR is used to probe modulations in brush conformation. Both techniques examine the brush as a function of temperature as well as solvent and electrolyte composition. Adopting this approach, we are able to examine the behavior and polymer conformation in colloidally unstable or poor solvent regimes, enhancing our understanding of SIE. Four distinct binary DMSO–water solvent compositions were selected to encompass the pure solvent conditions (*x*_D_ = 0 and *x*_D_ = 100), as well as LCST (*x*_D_ = 0.06) and UCST (*x*_D_ = 0.70) behavior for PNIPAM, which were informed by previous investigations.^6,42^ Cations were selected to ensure solubility across a broad range of solvent compositions, and anions were selected to reflect a broad range of þ values across the Hofmeister series: Cl^–^, Br^–^, I^–^, and SCN^–^; see Figure 1. In this work, we have for the first time taken the novel þ parameter, derived from quantum chemical computations, and compared its magnitude against an experimental ion-specific phenomenon against which it has not been previously bench-marked.

## Experimental Section

### Materials

Silicon wafers with a native oxide layer of approximately 2 nm used for spectroscopic ellipsometry (0.675 mm thick) were purchased from Silicon Valley Microelectronics (USA). Silicon blocks with a native oxide layer of approximately 1 nm (ø 100 mm; 10 mm thick) used for NR were purchased from El-Cat Inc. (USA). The sodium hydroxide (NaOH) used during surface cleaning and preparation was purchased from ChemSupply. Surface functionalization reagents triethylamine (TEA, 99%), (3-aminopropyl) triethoxysilane (APTES, >99%), and 2-bromoisobutyrate bromide (BIBB, >99%) were purchased from Sigma-Aldrich and used as received. Tetrahydrofuran (THF, >99%) was purchased from RCI Labscan Ltd. and then dried over 4 Å molecular sieves prior to use. Methanol and ethanol, used as polymerization cosolvents and to clean surfaces, respectively, were purchased from Thermo Fisher Scientific and used as received. Polymerization reagents copper bromide (CuBr_2_, 99.999%), 1,1,4,7,10,10-hexamethyltriethylenetetramine (HMTETA, 97%), and (+)-sodium l-ascorbate (>98%) were purchased from Sigma-Aldrich and used as received. The *N*-isopropylacrylamide (NIPAM) monomer was purchased from Sigma-Aldrich and purified by recrystallization from hexane (Sigma-Aldrich) prior to use. Potassium thiocyanate (KSCN, 99%), lithium thiocyanate (LiSCN, hydrate), lithium iodide (LiI, 99.9%), and lithium bromide (LiBr, ≥99%) were purchased from Sigma-Aldrich. Potassium iodide (KI) and potassium bromide (KBr) were purchased from Ajax Finechem, potassium chloride (KCl, ≥99%) was purchased from Fisher Chemicals, and lithium chloride (LiCl, anhydrous) was purchased from BDH laboratory supplies. All salts used for in situ measurements were dried at 110 °C prior to use. Dimethyl sulfoxide (DMSO, anhydrous, ≥99.9%) and deuterated dimethyl sulfoxide (DMSO-*d*_6_, anhydrous, 99.9 atom % D) were purchased from Sigma-Aldrich and used as received. Milli-Q water (Merck Millipore, 18.2 MΩ cm at 25 °C) was used throughout, excluding NR experiments, which used D_2_O (Sigma-Aldrich). All glassware was thoroughly washed with Milli-Q water and ethanol prior to washing in a 10% HNO_3_ acid bath for at least 24 h.

### Polymer Brush Synthesis

All polymer brushes for NR and spectroscopic ellipsometry were synthesized according to our previously reported method.^6,9,18^ However, as two different thickness regimes were required for spectroscopic ellipsometry and NR, two different polymerization protocols were utilized. In order to achieve appropriate polymerization kinetics for NR, the methanol/water solvent ratio was chosen as 4:1 v/v, and the monomer/catalyst/ligand/reducing agent molar ratio was 900/1/10/10 with NIPAM/CuBr_2_/HMTETA/sodium ascorbate.^6,18^ A significantly thicker film was required for spectroscopic ellipsometry to distinguish between the polymer and the solvent (i.e., DMSO) due to similar refractive indices. Hence, the reaction rate was increased by employing a monomer/catalyst/ligand/reducing agent molar ratio of 900/1.5/15/10 with NIPAM/CuBr_2_/HMTETA/ascorbic acid.^9^ A summary of the dry film thickness for each PNIPAM brush sample is presented in Table 1 (measured at standard laboratory conditions: *T* ≈ 22 °C; RH ≈ 30%). Hereon, all samples will be referred to by their dry brush thickness as measured by air–solid ellipsometry and reported in Table 1.

**Table 1:** Summary of PNIPAM Dry Brush Thickness^a^ as Determined by Air–Solid Ellipsometry and Neutron Reflectometry for Each Brush Sample Investigated during Specified In Situ Measurement

sample examined during specified in situ measurement	ellipsometrically determined dry brush thickness^b^ (Å)	NR determined dry brush thickness (Å)
ellipsometry; *x*_D_ = 0	306 ± 3	
ellipsometry	714 ± 6	
NR; K^+^, *x*_D_ = 0, 0.06, 1.0	198 ± 3	221 ± 1
NR; Li^+^, *x*_D_ = 0, 0.06	247 ± 5	262 ± 1
NR; *x*_D_ = 0.70	244 ± 7	260 ± 1

a Associated uncertainties for ellipsometry measurements are taken as the standard deviation from multiple measurements across the surface. For NR measurements, uncertainties are derived from PT-MCMC sampling; see details in the Neutron Reflectometry Analysis Section.

b The ellipsometrically determined dry brush thickness will be used as a sample identifier for subsequent experiments.

### Ellipsometry

All ellipsometry measurements were performed on the Accurion EP4 variable angle spectroscopic imaging ellipsometer at the Australian Centre for Neutron Scattering (ANSTO, Lucas Heights, Australia),^6,7,18^ with the exception of the dry film measurement for the 198 Å brush used for NR, which was measured on an EP3 single wavelength (532 nm) imaging ellipsometer. The thickness of this film was probed across four locations on the surface and five equally spaced angles of incidence from 50 to 70°. Dry film surface mapping of all other brushes was performed at a single wavelength (658 nm) across 31 points on the surface with four equally spaced angles of incidence from 40 to 70°. Spectroscopic in situ measurements were conducted on a single polymer brush encased by the standard solid–liquid cell. The incident radiation was 65° normal to the surface, and ten equidistant wavelengths were employed from 410 to 900 nm. All temperature cycles were performed with increasing temperature, and the sequence of experimental conditions was in the order of increasing DMSO content. A salt-free solvent flush was performed when changing to a new electrolyte identity. The selection of salts and concentrations studied reflect solubility in a wider range of solvents that are the subject of future investigations.

The analysis of ellipsometry data was performed with the *refellips* software package.^44^ For all dry measurements, the data were modeled using four components informed by previous investigations.^6,18^ The model consisted of four uniform layers (slabs), delineating each component’s optical properties, thickness, and roughness. From “fronting” to “backing” (i.e., in the direction of irradiation), the model structure was air, polymer, silica, and silicon. Fronting is the semi-infinite medium through which the radiation travels before it hits the interface of interest, the backing medium is the semi-infinite medium through which the transmitted beam travels away from the interface of interest.^45^ This model structure was also employed for in situ measurements of the “thin” PNIPAM film, where the air component was replaced with water. However, for in situ measurements of the “thick” PNIPAM film, a model analogous to our NR approach was employed, which utilizes a piecewise cubic hermite interpolating polynomial (PCHIP) with a single knot (spline control point) placed between the proximal polymer slab and the water-fronting media.^46^ Here, a simple slab is insufficient as it does not capture the diffuse nature of this significantly thicker brush.^47^ The model structure utilized for the in situ analysis was solvent, polymer spline, polymer slab, silica, and silicon. For both dry and in situ measurements, the model optical properties of the polymer brush were set using a Cauchy model, with parameters fixed at *A* = 1.45 and *B* = 0.005. A Cauchy model was also employed for the solvent fronting media, and parameters were permitted to vary to allow for variations in solvent composition as DMSO was incorporated into the system. Brush thickness was extracted from the model as twice the first moment of the corresponding polymer volume fraction (VF) profile
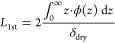
1where ϕ(*z*) is the VF of the polymer at distance *z* from the substrate and δ_dry_ is the interfacial volume of the dry film. The adsorbed amount of the polymer was constrained by the dry brush thickness. The temperature-modulated behavior of the “thick” PNIPAM brush in pure water as monitored by spectroscopic ellipsometry is presented in Figure S2.1. It is important to note that it was previously not possible to distinguish between the brush and solvent when at high *x*_D_.^6^ However, by increasing the total amount of polymer in the optical path, we are able to distinguish between the refractive indices of the polymer and solvent (i.e., DMSO) within the system. All code and data required to reproduce the analysis presented here are available on Zenodo.^48^

In pure water and DMSO-water binary mixtures of *x*_D_ = 0.06, PNIPAM exhibits a clear LCST thermoresponse.^6^ The LCST was obtained from ellipsometry data by modeling the brush thickness (*y*) as a function of temperature (*t*) as a generalized sigmoidal (i.e., logistical) function
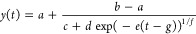
2where the resultant LCST can be extracted as *g* and the swollen film thickness is *a*; the remaining parameters define the shape of the transition. An example fit is presented in Figure S2.1, which returns a LCST of 32.2 °C for the “thick” PNIPAM brush in water. The choice of employing the generalized sigmoidal function to characterize the thermotransition stems from our previous investigation of PNIPAM in DMSO–water mixtures.^6^ The origin is the distinct shape of the PNIPAM brush thickness data obtained from spectroscopic ellipsometry (i.e., the lack of plateau in brush thickness at low temperatures in DMSO–water mixtures; e.g., Figure S2.5); a conventional sigmoidal function is not sufficient to locate the CST.

### Neutron Reflectometry

Specular NR measurements were collected on the PLATYPUS time-of-flight neutron reflectometer at the 20 MW OPAL nuclear reactor at the ANSTO, Lucas Heights, Australia.^49^ Air–solid measurements were collected at two angles of incidence, 0.65 and 3.0°, yielding a *Q*-range of 0.008–0.25 Å^–1^. The reflectivity for all in situ measurements was collected over a *Q*-range of 0.01–0.30 Å^–1^ with a constant dθ/θ = 0.033 and a 65 mm footprint. As per our previous in situ investigations, samples were placed in the standard solid–liquid cells encased by temperature jackets, with reflectivity measured in an upward-reflecting geometry.^6,7,18,46^ Consistent with spectroscopic ellipsometry measurements, all temperature ramps were performed with increasing temperature, and the cell was flushed with pure solvent prior to the addition of each different electrolyte.

### Neutron Reflectometry Analysis

All specular NR data were reduced and modeled using the *refnx* software package.^46,50^ We employ a model for the polymer brush system informed by previous investigations,^6,7,46^ and consistent with our spectroscopic ellipsometry analysis approach for both dry and in situ measurements. Briefly, the model comprised two main components: slabs and a PCHIP. These components contain parameters regarding the respective thickness, roughness, VF of the solvent, and scattering length density (SLD) of each layer. From “fronting” to “backing”, the model structure contained slabs for the silicon, silica, proximal polymer layers, and solvent. A PCHIP was then employed subsequent to the proximal polymer slab to describe the VF profile of the diffuse brush periphery. The PCHIP employed for in situ NR analysis contained four knots, whereby the VF and distance between each knot were permitted to vary. The adsorbed interfacial volume was constrained by the dry brush thickness (Table 1) and monotonic VF profiles were enforced across all conditions.

The theoretical SLD profile, ρ_N_(*z*), is then calculated from the polymer VF profile, ϕ(*z*), via^8,46^
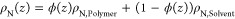
3where ρ_N,Polymer_ and ρ_N,Solvent_ represent the SLD of the polymer and solvent, respectively, and *z* is the orthogonal distance from the substrate, where *z* = 0 represents the interface between the oxide and polymer. The Abeles matrix formalization was then employed to determine the respective reflectivity profile, which was subsequently compared to the experimentally determined reflectivity. Parallel tempered Markov chain Monte Carlo (PT-MCMC) simulations were then used to sample the posterior distribution of the data in search of the most probable set of model structures. All optimized fits and polymer VF profiles presented in this study are extracted as the median from the PT-MCMC samples, which all showed narrow distributions. The temperature-modulated structure of a 198 Å PNIPAM brush in D_2_O is presented in Figure S3.1. Further details regarding this modeling approach are explored by Gresham et al.,^46^ with all relevant data and code required to reproduce these analyses available on the Zenodo repository.^48^

## Results and Discussion

The modulation of polymer conformation and thermoresponse was monitored as a function of electrolyte identity and solvent (DMSO–water) composition. Initially, we measured the baseline behavior of the PNIPAM brush in water (*x*_D_ = 0) with potassium and lithium salts of Cl^–^, Br^–^, I^–^, and SCN^–^. The thermoresponse and changes in brush thickness were monitored using in situ spectroscopic ellipsometry. Modulations in brush nanostructure were investigated with in situ time-of-flight NR. The behavior of PNIPAM is first examined in electrolyte solutions of the two pure solvents: water and DMSO. The response of PNIPAM brushes is then investigated in two distinct DMSO–water solvent compositions: water-rich (*x*_D_ = 0.06) and DMSO-rich (*x*_D_ = 0.70). We conclude with a comparison of behavior across all solvent compositions. Herein, we use the terms salting-in and salting-out to describe changes in brush thickness and LCST, i.e., data derived from spectroscopic ellipsometry. The two electrolyte concentrations selected throughout this work were 0.2 and 0.9 mol %, which are equivalent to approximately 100 and 500 mM in water. We report electrolyte concentrations as mol % to maintain a constant solute/solvent molecule ratio.

### Pure Solvents

#### 0 mol % DMSO

**Figure 2:**
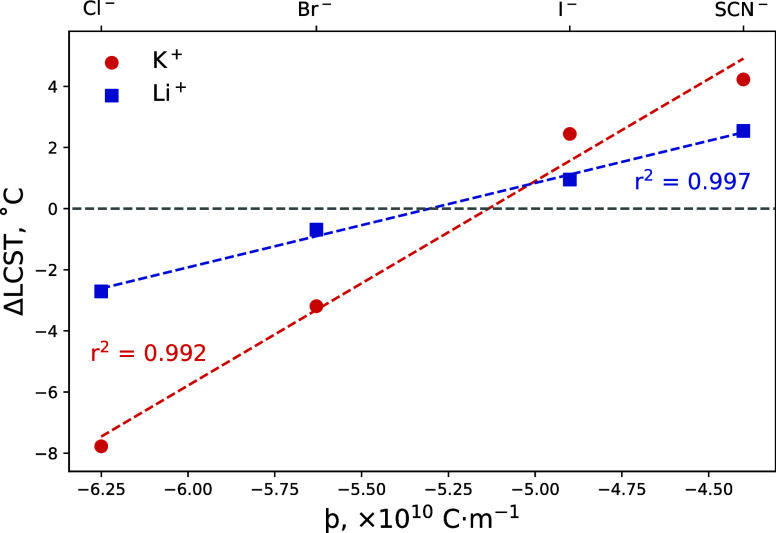
Change in LCST relative to pure water (LCST = 32.1 °C) as a function of anion þ value for the 306 Å PNIPAM brush in various 0.9 mol % aqueous potassium and lithium electrolytes. Top *x*-axis identifies ions probed and lower *x*-axis shows their respective þ value. Dashed lines are linear fits for potassium (K^+^; red circles) and lithium (Li^+^; blue squares) data. Uncertainty is smaller than the data point.

Spectroscopic ellipsometry was employed to establish the baseline behavior of a 306 Å PNIPAM brush (i.e., the dry brush sample thickness, see Table 1) in water (*x*_D_ = 0) across the 0.9 mol % electrolytes of interest: potassium and lithium salts of Cl^–^, Br^–^, I^–^, and SCN^–^. The brush thickness was monitored as a function of temperature, and the LCST was identified via eq 2. The resultant changes in LCST in each electrolyte relative to the salt-free condition, as a function of the anion’s þ value,^30^ are presented in Figure 2. All þ values are taken from the work of Gregory et al. and are presented in Table S1.1.^30^ Figures presenting data for the change in brush thickness as a function of temperature for each electrolyte are presented in Figure S2.2.

As expected, both Cl^–^ and Br^–^ impart a salting-out effect on the brush, decreasing the LCST below that observed in pure water. In comparison, SCN^–^ and I^–^ manifest a salting-in effect, increasing the LCST relative to water. The ΔLCST yields a strong linear relationship with anion þ for both counter-cations, where the direction of influence imparted by each anion (i.e., salting-in/out) aligns with previous investigations of PNIPAM and POEGMA, both grafted and ungrafted.^8,9,19,31,32^ Gregory et al. have demonstrated for many thermoresponsive polymers in aqueous electrolytes that the ion-modulated thermoresponse exhibits a strong linear dependence on the ion’s radial charge density: þ.^30^ The authors posited that while ion–polymer interactions were strongly correlated with þ, ion–water interactions prevailed and were the dominant driving force behind the modulated thermoresponse.

Figure 2 illustrates that the direction of the SIE each anion imparts on the brush is agnostic of the cation identity, with the cation modulating the magnitude of the response; K^+^ yields a stronger effect (i.e., a steeper gradient) than Li^+^. Notably, the deviation of KSCN from linearity is expected due to the anisotropy of the SCN^–^ anion, and hence the presence of multiple interaction sites; recall that þ is interaction site-specific. In this multicomponent system, both cation–anion and cation–PNIPAM interactions also occur. However, as Li^+^ (31.61 × 10^–10^ C/m) has a much higher radial charge density than K^+^ (9.24 × 10^–10^ C/m) and suppresses the magnitude of each anion’s effect, it would appear that a strengthening of cation–anion interaction weakens the anionic effect, as opposed to a direct cation–PNIPAM interaction. In the latter case, we might expect a vertical shift in the data instead of the gradient. Previous investigations of PNIPAM-modified silica particles in KCl and LiCl electrolytes by Humphreys et al. also showed that the anion was more important in dictating the overall influence of the electrolyte.^10^ Gregory et al. later noted a linear relationship between the ΔLCST in the above work by Humphreys et al. and þ.^30^ Recent computational advances have hypothesized that cation-specific effects are typically more complex than anion-specific effects due to the similarity in electrostatic cation–solvent and cation–solute interactions, ultimately enabling non-negligible dispersion interactions.^29,30^ Moreover, the magnitude of the impact of cations on measured SIE correlates poorly with the cation þ value.^30^ Consistency across various brush samples is also observed, as analogous results were obtained for the 714 Å PNIPAM brush. Brush thickness as a function of temperature and the extracted ΔLCST as a function of þ for the thick PNIPAM brush are presented in Figures S2.3 and S2.4, respectively. The changes in polymer molecular weight between the “thick” and “thin” brushes investigated appear to play a nondominant role in the manifestation of SIE on the thermoresponse of the brush.

**Figure 3:**
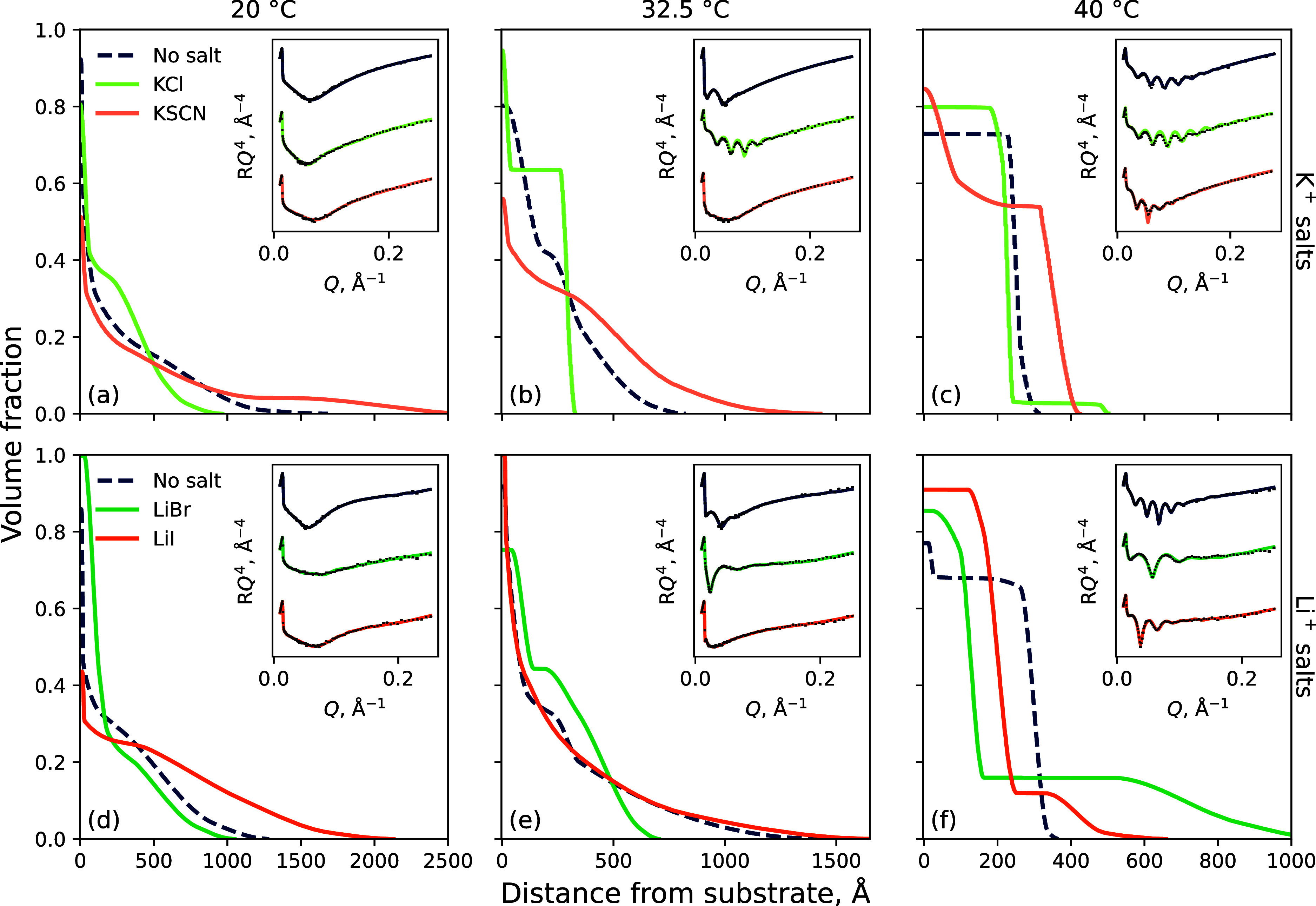
NR determined polymer VF profiles of the (a–c) 198 Å PNIPAM brush in aqueous potassium electrolytes and the (d–f) 247 Å PNIPAM brush in lithium electrolytes at (a,d) 20, (b,e) 32.5, and (c,f) 40 °C. Insets present the measured reflectivity with superimposed optimized models. Each “no salt” condition corresponds to the respective brush probed. Electrolyte concentration is fixed at 0.9 mol %; electrolytes of 0.2 mol % are presented in Figure S3.2. Spread of fits is presented in Figure S4.1.

The impact of these electrolytes on the temperature-dependent conformation of a PNIPAM brush was then investigated with NR. Consistent with previous investigations,^6,18^ we describe changes in polymer conformation relative to three main components: the proximal region adjacent to the silica substrate, the inner region of the brush, and the outer diffuse tail. The behavior of the brush was probed at three temperatures that capture the swollen, transition, and collapsed regimes of a PNIPAM brush in water: 20, 32.5, and 40 °C. Figure 3 presents the polymer VF profile of the brush in pure D_2_O and 0.9 mol % (a–c) potassium electrolytes and (d–f) lithium electrolytes. The impact of 0.2 mol % of these electrolytes on the PNIPAM brush structure is presented in Figure S3.2.

Briefly, at 20 °C and in the absence of salt (Figure 3a,d; dark blue-dashed), the brush is in its relatively most swollen state with a low polymer VF in the inner region. Upon increasing the temperature to 32.5 and 40 °C, the solvation of the brush decreases; the polymer VF in the inner region has increased, and the extent of the brush normal to the surface has decreased. Specifically, at 40 °C, the brush has collapsed into a slab-like conformation. We note the presence of the previously demonstrated “bottom-up collapse”^3,51^ and can conclude that the behavior of both brushes used in the NR experiment is very similar and consistent with previous NR studies of PNIPAM brushes.^6,8,18^ Upon the addition of salt, the changes in polymer conformation are as expected; SCN^–^ and I^–^ are anticipated to promote brush swelling and Cl^–^ and Br^–^ promote brush collapse.^8,19^ In the presence of a salting-in salt (i.e., KSCN and LiI), the brush is seen to be more solvated relative to the pure water condition. Conversely, upon exposing the brush to a salting-out salt (i.e., KCl and LiBr), polymer solvation decreases, and the brush is seen to collapse relative to water. Both of these phenomena are most apparent at 32.5 and 40 °C. Interestingly, however, in its most collapsed state at 40 °C, the periphery of the brush is more swollen in the presence of LiBr than in its absence or the presence of LiI. The changes in brush structure in the presence of salt revealed by NR are concordant with spectroscopic ellipsometry measurements, which probe changes in brush swelling and thermotransition.

#### 100 mol % DMSO

**Figure 4:**
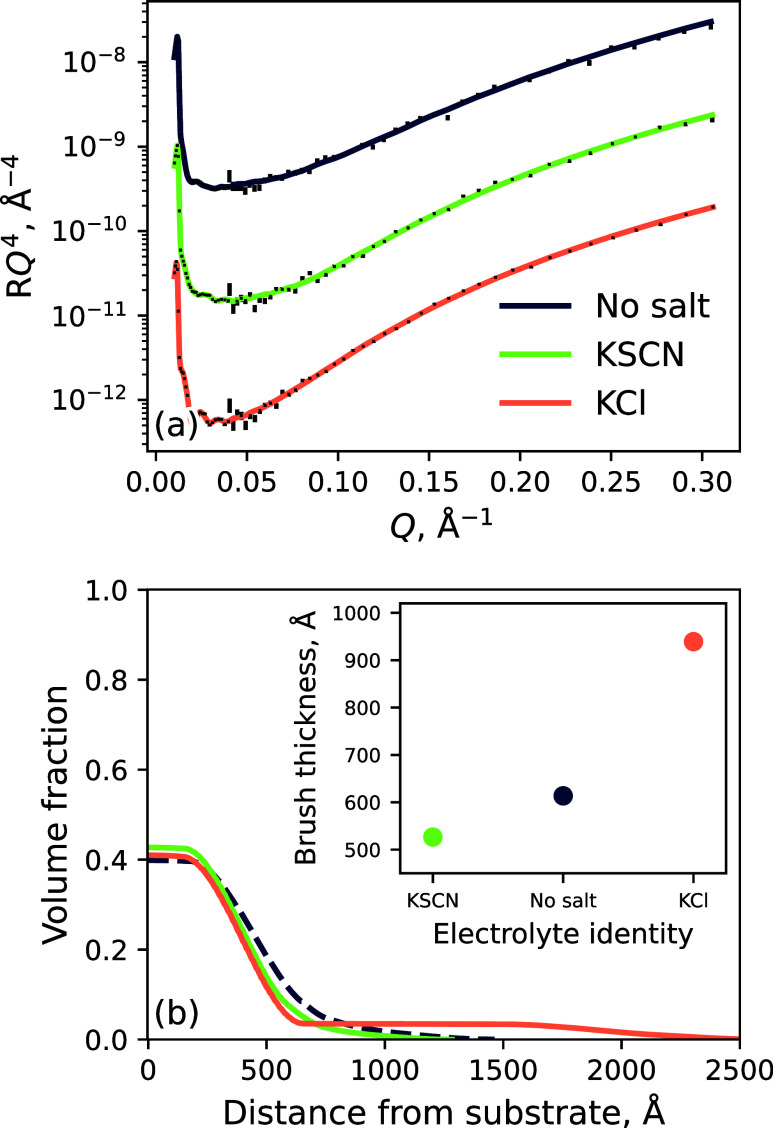
(a) Neutron reflectivity with superimposed models and (b) polymer VF profiles of the 198 Å PNIPAM brush in 100 mol % DMSO salt solutions of 0.2 mol % KSCN (green) and KCl (orange). Inset in (b) presents the solvated brush thickness in pure DMSO, 0.2 mol % KSCN, and 0.2 mol % KCl as determined by the VF profile first moment. Data presented here was captured at 32.5 °C. Spread of fits is presented in Figure S4.4.

In pure DMSO (*x*_D_ = 1.0), PNIPAM is known to not exhibit a thermoresponse: no significant change in brush swelling or structure with temperature is observed.^6^ Interestingly, the addition of salt to the pure DMSO system does induce changes to the brush swelling state. Figure 4 presents the NR-derived change in polymer conformation and swelling in 0.2 mol % KSCN and KCl electrolytes in DMSO at 32.5 °C. Subtle changes in brush structure relative to pure DMSO are observed for both electrolytes, while discernible changes in brush thickness are measured. Across all conditions probed, the brush exhibits a diffuse periphery with a well-solvated inner region. In 0.2 mol % KSCN, the brush thickness is observed to be less than the thickness in pure DMSO; however, in 0.2 mol % KCl, the PNIPAM chains are more solvated, and an increased thickness is observed. Here, we observe a solvent-induced specific ion series reversal in pure DMSO. We hypothesize that the type of SIE manifested depends on the delicate balance of solvent aggregation, polymer solvation, and ion solvation. In particular, SCN^–^ anions stabilize DMSO aggregates via an entropically favorable hydrophobic interaction, reducing the number of solvent molecules needed to solvate the brush; the brush is desolvated or salted-out. Conversely, Cl^–^ anions destabilize DMSO aggregates, increasing the number of available solvent molecules to solvate PNIPAM. Furthermore, as a result, we propose that some associated Cl^–^ anions are excluded from the bulk solvent and penetrate the brush, increasing brush swelling by excluded volume effects and electrostatic repulsion via polymer–anion association. Previous investigations by Mazzini et al. present concordant results, demonstrating the solvent-modulated direction of the Hofmeister series: a Hofmeister series reversal in pure DMSO.^33^ Size exclusion chromatography experiments revealed that in *x*_D_ = 1.0, SCN^–^ and I^–^ were strongly solvated, whereas Cl^–^ and Br^–^ were weakly solvated; the inverse to water. The authors also demonstrated a series reversal by probing the ion-induced swelling modulation of a PMETAC brush via quartz crystal microbalance with dissipation monitoring (QCM-D).

### DMSO–Water Mixtures

#### 6 mol % DMSO

**Figure 5:**
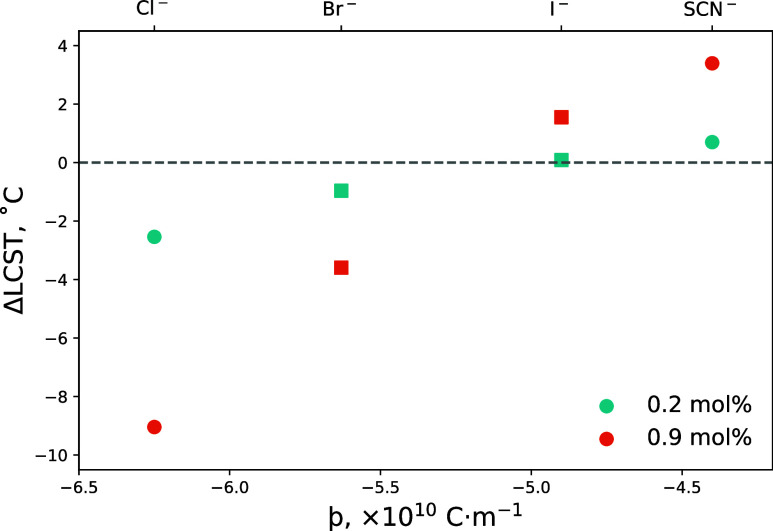
Ellipsometrically derived changes in LCST (relative to no salt; LCST = 29.3 °C) of the 714 Å PNIPAM brush in electrolytes composed of 0.2 and 0.9 mol % KSCN, LiI, LiBr, and KCl in a 6 mol % DMSO solvent composition. Top *x*-axis identifies the probed anions and the lower their respective þ values. Square symbols represent a Li^+^ counter-cation and circle symbols represent a K^+^ counter-cation. Corresponding changes in brush thickness as a function of temperature are presented in Figure S2.5. Uncertainty is smaller than the data point.

We have previously investigated the behavior of a PNIPAM brush in salt-free binary DMSO–water solvents.^6^ Briefly, in *x*_D_ = 0.06, PNIPAM exhibits LCST-type behavior where the thermotransition is shifted to lower temperatures relative to water. This decrease in LCST arises from the formation of favorable DMSO-water aggregates leading to a decreased availability of free water to solvate polymer chains. Here, we extend the previous study to investigate SIE in binary DMSO–water electrolytes. The ellipsometrically-derived change in LCST of a 714 Å PNIPAM brush as a function of both anion identity and electrolyte concentration is presented in Figure 5. The corresponding change in thickness of this PNIPAM brush in *x*_D_ = 0.06 as a function of temperature is presented in Figure S2.5. Both KCl and LiBr again impart a salting-out effect on the brush, decreasing the LCST relative to water, whereas KSCN and LiI yield a salting-in effect on the brush. Across all examined anions, the magnitude of the effect is seen to increase with increasing electrolyte concentration. Moreover, for both electrolyte concentrations examined, SCN^–^ imparts the greatest salting-in effect and Cl^–^ the greatest salting-out effect. Both of these phenomena are analogous to measurements of a PNIPAM brush in electrolytes of pure water (Figures 2, 3, and S2.4), and the changes in LCST correlate well with þ.

In order to unravel the observed SIE, we must consider the impact of both the anion and cation on the formation of DMSO–water aggregates. From the examination of the trends for each concentration in Figure 5, the strength of the cation in modulating the magnitude of the observed effect seems to have diminished greatly in *x*_D_ = 0.06. We hypothesize that in *x*_D_ = 0.06, the cations favor the formation of a DMSO–cation complex; that is, both K^+^ and Li^+^ possess a similar propensity for interacting with DMSO. As a direct consequence of this DMSO–cation interaction, we hypothesize that the dependence of the manifested specific effect on the cation identity is decreased relative to when the solvent is pure water (Figure 2). In that case, this may result in increased availability of water molecules to solvate the brush or the (more strongly hydrated) anions.

**Figure 6:**
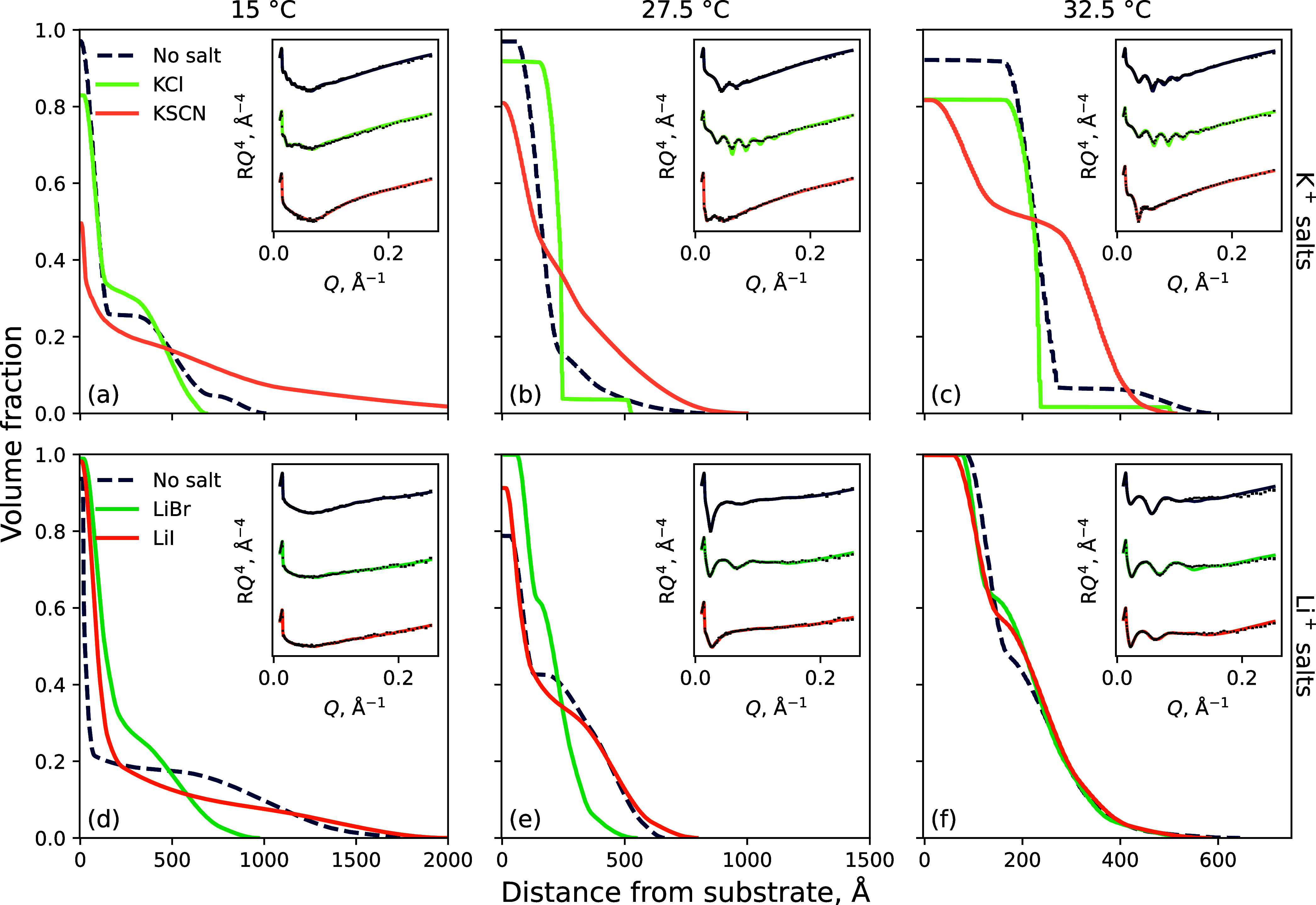
Polymer VF profiles derived by NR of the (a–c) 198 Å PNIPAM brush in 6 mol % DMSO potassium electrolytes and the (d–f) 247 Å PNIPAM brush in lithium electrolytes at (a,d) 15 °C, (b,e) 27.5, and (c,f) 32.5 °C. Electrolyte concentration is fixed at 0.9 mol %; electrolytes of 0.2 mol % are presented in Figure S3.3. Insets present the measured reflectivity with superimposed optimized models. Each “no salt” condition corresponds to the respective brush probed. Spread of fits is presented in Figure S4.2.

NR of a PNIPAM brush in these 6 mol % DMSO electrolytes (Figure 6) yielded behavior concordant with the spectroscopic ellipsometry results. Changes in polymer conformation were monitored at 15, 27.5, and 32.5 °C to capture the polymer VF profiles in the swollen, transition, and collapsed regimes, respectively, in the “no salt” *x*_D_ = 0.06 binary solvent. Here, the brush behavior is analogous to that exhibited in pure water (Figure 3), undergoing collapse with increasing temperature.^6^ Upon addition of 0.9 mol % KSCN or LiI, the brush appears to be more swollen relative to the pure solvent condition, as the VF of solvent in the inner region decreases and the diffuse region extends out further from the substrate. Conversely, in the presence of KCl or LiBr, the brush exhibits a more collapsed structure at each temperature examined. Specifically, coupled with a decreased brush extent, the VF of polymer in the inner region of the brush increases relative to the pure solvent condition. The impact of 0.2 mol % electrolytes in *x*_D_ = 0.06 solvents is presented in Figure S3.3.

Liu et al. have investigated changes in the LCST of ungrafted PNIPAM in sodium electrolytes in DMSO–water binary solvents composed of *x*_D_ = 0.06 via turbidity measurements.^42^ Overall, the authors note concordant behavior to that seen in our brush system, observing that in a salt-free solvent composition of *x*_D_ = 0.06, the LCST decreases relative to pure water. Upon the addition of NaSCN, the thermotransition was seen to increase slightly; salting-in free PNIPAM. Conversely, in the presence of NaCl, salting-out behavior was observed, resulting in a decrease in the thermotransition.

#### 70 mol % DMSO

**Figure 7:**
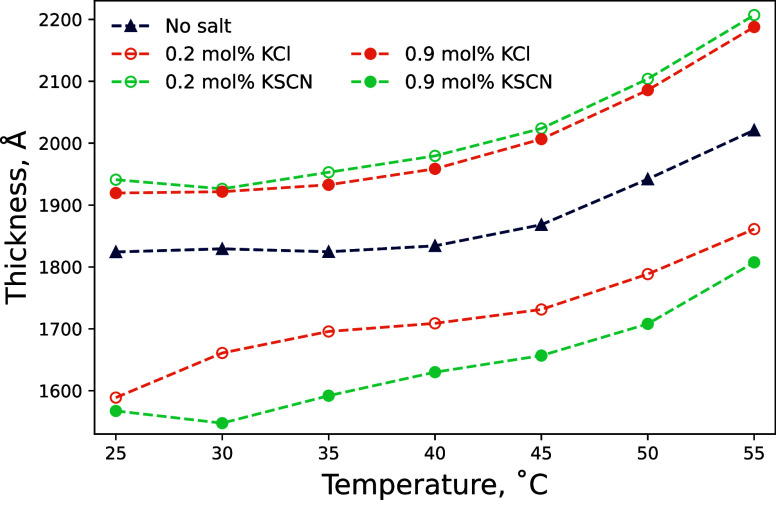
Ellipsometrically determined polymer film thickness of the 714 Å PNIPAM brush in KSCN and KCl electrolytes in 70 mol % DMSO–water solvent mixtures as a function a temperature. Open symbols correspond to 0.2 mol % and closed symbols correspond to 0.9 mol %. Corresponding changes in film thickness for the brush exposed to LiI and LiBr are presented in Figure S2.6. Uncertainty is smaller than the data point.

In a binary solvent of *x*_D_ = 0.70, both grafted and ungrafted PNIPAM are known to exhibit UCST behavior: a desolvated to solvated (collapsed to swollen) transition with increasing temperature.^6,42^ The phenomenon is illustrated in Figure 7, which presents the ellipsometrically determined brush thickness as a function of increasing temperature. In line with previous investigations,^6^ in *x*_D_ = 0.70, the thickness of the PNIPAM film is seen to increase with increasing temperature. In this study, we overcame previous refractive index contrast limitations between the polymer and solvent by effectively increasing the amount of polymer present, i.e., increasing the brush thickness.

Figure 7 presents PNIPAM brush thickness as a function of temperature for 0.2 and 0.9 mol % solutions of KSCN and KCl electrolytes in a 70 mol % DMSO–water solvent mixture. Analogous behavior is observed for LiBr and LiI in Figure S2.6. The thermoresponse exhibited by PNIPAM in *x*_D_ = 0.70 and KSCN or KCl is significantly broader relative to the transition exhibited in pure water or *x*_D_ = 0.06. Similar to the LCST behavior exhibited at low *x*_D_, the brush thickness and thermotransition can be modulated by both salt identity and concentration, though here the relationship is more complex. In 0.2 mol % electrolytes (open symbols) of KCl and KSCN, the brush thickness is modulated as expected based on PNIPAM brush behavior in water and 6 mol % DMSO–water solvents: KCl decreases the brush thickness at a given temperature relative to the pure solvent condition, whereas KSCN increases the brush thickness. In contrast, increasing the electrolyte concentration to 0.9 mol % in *x*_D_ = 0.70 does not simply increase the magnitude of the effect, as seen for water and *x*_D_ = 0.06 electrolytes. Rather, in *x*_D_ = 0.70, the impact of 0.9 mol % Cl^–^ and SCN^–^ anions has swapped: Cl^–^ is now salting-in the brush, whereas SCN^–^ is salting-out the brush. This behavior is consistent with that observed for pure DMSO in Figure 4 and will be further discussed below.

We have previously argued that polymer solvation in a salt-free 70 mol % solvent is governed by the stability of solvent clusters: water molecules will preferentially form DMSO–water clusters over PNIPAM–water interactions.^6^ These DMSO–water clusters face entropic limitations and are unable to penetrate deep into the brush; the remaining DMSO molecules form non-site-specific interactions (DMSO–PNIPAM and DMSO–DMSO) and govern polymer solvation. The UCST behavior exhibited by PNIPAM in *x*_D_ = 0.70 has been attributed to the thermal disruption of DMSO–water hydrogen bonds, rupturing solvent aggregates and increasing polymer solvation with increasing temperature.^6^ In this study, we increase the system’s dimensionality to include charged species. However, we propose that solvent cluster stability continues to govern the manifested SIE and brush behavior.

Polymer solvation in *x*_D_ = 0.70 is strongly governed by the bulk solvent structure (DMSO–water clusters). We propose that solvent cluster stability governs the manifested SIE and brush behavior as it is acutely sensitive to the identity and concentration of ions present. In *x*_D_ = 0.70 electrolytes, there exists a delicate balance between the entropy and enthalpy governing polymer solvation and intermolecular solvent association. We have observed a concentration-dependent specific ion series: a forward Hofmeister series at 0.2 mol % and a reverse Hofmeister series at 0.9 mol %. In 0.2 mol % electrolytes composed of *x*_D_ = 0.70, the behavior of PNIPAM is concordant with that in water-rich regimes (*x*_D_ = 0 and *x*_D_ = 0.06). Investigations by Liu et al. suggest that both SCN^–^ and Cl^–^ weakly interact with solvent aggregates via the polarization of the DMSO–water hydrogen bond.^42^ We suggest that the strongly solvated Cl^–^ anion will also interact with available solvent molecules and DMSO–DMSO aggregates, reducing the number of free solvent molecules available to solvate the polymer brush. At the lower concentration examined here, despite a favorable interaction of SCN^–^ with the solvent clusters, a preferential interaction with the hydrophobic backbone of PNIPAM acts to increase polymer stability. The result is a net salting-in effect by KSCN and a salting-out effect by KCl.

Upon increasing the electrolyte concentration to 0.9 mol %, a series reversal is observed due to a shift in the balance between polymer solvation and intermolecular solvent association, results that are analogous to those observed in pure DMSO. We suggest that the manifested SIE is dictated by the change in the nature of the anion–solvent interactions with increasing salt concentration. We suggest two mechanisms at play resulting in Cl^–^ yielding a salting-in effect at a high concentration. The first is that the strong interaction between solvent aggregates and Cl^–^ anions prevails, and results in Cl^–^ salting-out the solvent clusters; Cl^–^ disrupts the aggregates, releasing solvent molecules, which can subsequently solvate PNIPAM. Second, the weaker solvent interaction with chloride tips the balance in favor of anion–polymer interactions. This in turn produces a salting-in effect due to charge repulsion between polymer chains and excluded volume effects. In the case of SCN^–^, non-site-specific anion–solvent interactions also prevail, as SCN^–^ interacts with the hydrophobic domain on both bulk DMSO and solvent aggregates. This ultimately drives solvent aggregate stability (i.e., salting-in solvent aggregates), which in turn results in a net salting-out effect of PNIPAM. As a further consequence of the increased SCN^–^–solvent interaction, there is a greater entropic barrier to penetrate the brush due to the presence of both SCN^–^ anions and relatively “bulky” solvent aggregates.

Comparisons for this role of ternary system complexity (here, the role of the solvent composition) are our previous investigations into SIE, exploring the role of the solute and substrate, respectively.^7,52^ First, in mixed electrolytes, NR and ellipsometry revealed anions changing the nature of their influence on the conformation of a POEGMA brush (e.g., changing from salting-in to salting-out) due to a shift in the delicate balance of interactions.^7^ Moreover, in statistical copolymers composed of both neutral and weakly basic monomer moieties, poly(2-(2-methoxyethoxy) ethyl methacrylate) (PMEO_2_MA) and poly(2-(diethylamino)ethyl methacrylate) (PDEA), respectively, NR revealed the manifested SIE were dependent by the nature of the ion–polymer interactions, namely, the degree of polymer charge dictated the presence of a forward or reverse Hofmeister series.^52^ The principles behind the balance of interactions in these previously examined ternary systems are not dissimilar to those observed here, whereby increasing the concentration of one species results in a nonmonotonic SIE trend, exhibiting a “turnover” in anion type with increasing concentration.

Previous investigations by Liu et al. on untethered PNIPAM in 0.1 M electrolytes in *x*_D_ = 0.70 illustrate salting-in behavior for all anions;^42^ the manifested SIE simply impacted in magnitude, not type. The authors probed the behavior of untethered PNIPAM of a higher molecular weight than examined in this study, meaning a greater local concentration of polymer was present. The authors concluded a similar mechanism, whereby there exists a competition between anion polarization of solvent aggregates via the polarization of the DMSO–water hydrogen bond and anion adsorption onto the surface of PNIPAM. However, given the concentration-dependent SIE present in *x*_D_ = 0.70, it is important to note that Liu et al. fixed the salt concentration at 0.1 M in *x*_D_ = 0.70, which is equivalent to approximately 0.4 mol %; a concentration in-between the two concentrations examined in this study.

**Figure 8:**
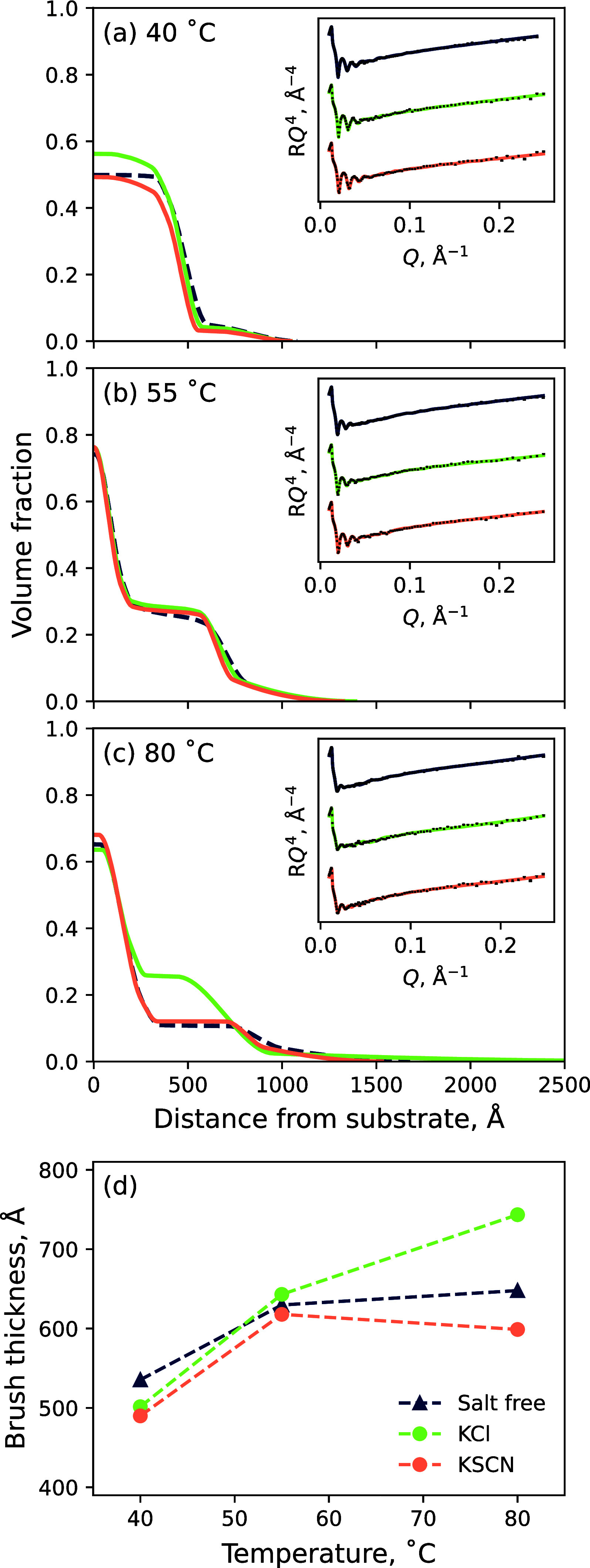
NR-derived polymer VF profiles of the 244 Å PNIPAM brush in a 70 mol % DMSO solvent and 0.9 mol % electrolytes of KCl and KSCN at (a) 40, (b) 55, and (c) 80 °C. (d) Respective brush thicknesses for each condition as derived from the VF profile first moment. All 0.2 mol % conditions are presented in Figure S3.4 and 0.9 mol % conditions presented in Figure S3.5. Insets present the measured reflectivity with superimposed optimized models. Spread of fits is presented in Figure S4.3.

NR was employed to investigate modulations in polymer conformation as a function of solvent and electrolyte identity across 40, 55, and 80 °C, chosen to again capture the brush across a collapsed, transition, and swollen state, respectively. Polymer VF profiles of a PNIPAM brush in *x*_D_ = 0.70 (Figure 8) illustrate concordant results to those presented in Figure 7, demonstrating that the PNIPAM brush in *x*_D_ = 0.70 swells with increasing temperature. Subtle changes in brush structure in 0.9 mol % electrolytes show complementary results to ellipsometry data, demonstrating that KCl yields a more swollen brush and KSCN yields a collapsed brush relative to pure 70 mol % DMSO. The ion-modulated brush structure can also be seen by examining changes in the first moment of the polymer VF profile (Figure 8d). The full suite of polymer VF profiles illustrating the impact of 0.2 mol % electrolytes on brush structure is presented in Figure S3.4 and 0.9 mol % electrolytes in Figure S3.5.

### Comparison across Solvent Compositions

**Figure 9:**
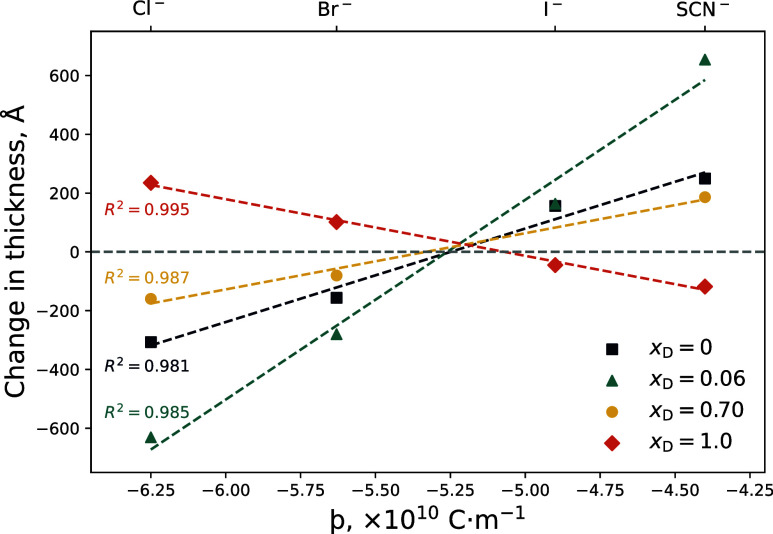
Changes in film thickness from ellipsometry of the 714 Å PNIPAM brush at the respective CST in various solvent compositions and electrolyte identities: KCl, LiBr, LiI, and KSCN. Top *x*-axis identifies the probed anions and the lower identifies their respective þ values. All electrolyte concentrations are 0.2 mol %. Change in thickness is relative to the “no salt” condition for each respective solvent composition. The chosen temperature was constant for each solvent composition and was selected to best represent the thermotransition region: 32.5 °C for water; 26 °C for 6 mol % DMSO; and 55 °C for 70 mol % DMSO. The intermediate temperature of 40 °C was selected for pure DMSO, which does not exhibit a thermotransition. Uncertainty is smaller than the data point.

**Figure 10:**
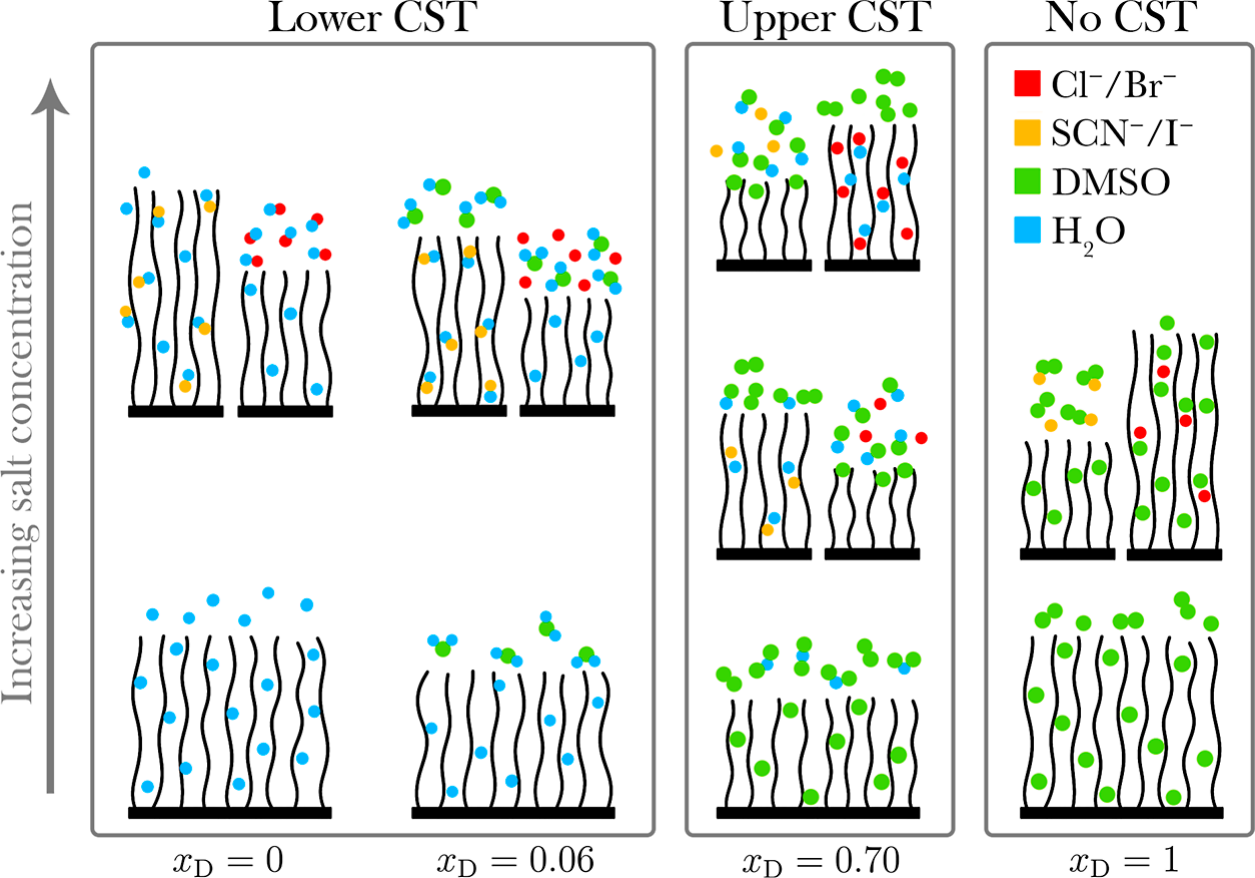
Schematic diagram of a polymer brush in DMSO–water electrolytes as presented herein. Contour lengths across all brushes are invariant; swelling and collapse behavior only are shown. Increasing DMSO mole fraction (*x*_D_) within the solvent composition is represented on the horizontal axis (*x*_D_ = 0, 0.06, 0.70, and 1) and increasing salt concentration is represented on the vertical axis (0, 0.2, and 0.9 mol %). A forward series is noted for the polymer brush in water-rich electrolytes and in 0.2 mol % electrolytes of *x*_D_ = 0.70. Concentration-induced series reversal is noted in *x*_D_ = 0.70 electrolytes from 0.2 to 0.9 mol %. Solvent-induced series reversal is noted in *x*_D_ = 1.

To summarize the results across the four solvent compositions examined (*x*_D_ = 0, 0.06, 0.70, and 1.0), we present a comparison (Figure 9) of the change in brush thickness in 0.2 mol % electrolytes (relative to the salt-free condition of that particular solvent composition) as a function of þ for each solvent composition. Presenting the data as a change in thickness rather than a change in CST decouples salt-specific and temperature effects. The electrolyte concentration was fixed at 0.2 mol % to allow for comparisons across all solvent compositions due to solubility constraints, and the temperature was selected as the thermotransition for each solvent composition. Figure S2.7 presents this alternative figure with 0.9 mol % electrolyte data in *x*_D_ = 0.70, with a visual representation illustrated in Figure 10.

In 0.2 mol % electrolytes composed of *x*_D_ = 0, 0.06, and 0.70, the forward Hofmeister series is manifested. However, in pure DMSO (*x*_D_ = 1.0), a reverse Hofmeister series is prevalent. The dashed lines in Figure 9 illustrate the strong linear correlation between the resultant change in brush thickness as a function of the anion þ value: positive gradients indicate a forward Hofmeister series; negative gradients indicate a reverse Hofmeister series. We hypothesize that the cation SIE has a stronger impact on the LCST (e.g., Figure 2), and changes to brush swelling (e.g., Figure 9) are negligible. Interestingly, irrespective of the solvent composition, the region around a theoretical þ value of approximately −5.2 × 10^–10^ C m^–1^ yields a null change in brush thickness. We propose that a hypothetical anion with this approximate þ value would not influence the swelling, thermoresponse, or structure of a PNIPAM brush. This phenomenon can also be seen in Figures 2 and 5, even though these data are related to changes in the LCST. Due to the persistence of this null þ value across all solvent conditions examined here, we hypothesize that this value is explicitly dictated by polymer–ion interactions rather than indirect ion–solvent interactions. Future studies will seek to further understand the universality of þ ≈ −5.2 × 10^–10^C m^–1^. Understanding how various ions impact colloidal stability is imperative for developing and enhancing applications, in particular lithium for energy storage and batteries.^19^

## Conclusions

This study investigated the changes in PNIPAM thermotransition, swelling, and conformation as a function of both solvent and electrolyte identity. PNIPAM was examined across four different solvent compositions, encompassing a LCST regime in the water-rich region (*x*_D_ = 0 and *x*_D_ = 0.06), a UCST regime in the DMSO-rich region (*x*_D_ = 0.70), and a suppression of the thermoresponse in pure DMSO (*x*_D_ = 1.0). Spectroscopic ellipsometry was employed to monitor changes in brush swelling to deduce modulations in thermotransitions. NR was used to probe solvent and electrolyte-induced modulations in brush structure.

In water (*x*_D_ = 0), the ion-modulated behavior of PNIPAM aligned with previously reported investigations. Notably, however, we examined how both K^+^ and Li^+^-based electrolytes impacted the phase transition and brush conformation of PNIPAM, salts that are of current pertinence to energy storage. A strong correlation between the change in PNIPAM LCST and the þ value (interaction-site-specific radial charge density parameter) was observed. The behavior of PNIPAM brushes in electrolytes in *x*_D_ = 0.06 was analogous to the behavior of the brushes in water, whereby both SCN^–^ and I^–^ anions induced a salting-in effect, increasing polymer solubility. Conversely, Cl^–^ and Br^–^ induced salting-out behavior on the brush, decreasing polymer solubility. Again, the influence of these ions on the LCST of PNIPAM is strongly correlated with þ.

The behavior of PNIPAM in binary solvents of *x*_D_ = 0.70 was also probed, with spectroscopic ellipsometry revealing a broad UCST thermotransition. In the presence of 0.2 mol % electrolytes, this thermotransition was modulated concordant with ion-specific behavior in water-rich solvents: SCN^–^ and I^–^ increase polymer solubility, while Cl^–^ and Br^–^ decrease polymer solubility. Interestingly, however, upon increasing the concentration of the electrolyte to 0.9 mol %, an opposing phenomenon is observed: SCN^–^ and I^–^ decrease polymer solubility, while Cl^–^ and Br^–^ increase solubility; a reverse Hofmeister series. This concentration-dependent ion-specific modulation of PNIPAM was attributed to the delicate balance between polymer solvation and solvent aggregation. In electrolytes composed of pure DMSO (*x*_D_ = 1.0), no thermotransition is observed; however, the degree of swelling was modulated by SIE. Indeed, again, a reverse Hofmeister series was present: SCN^–^ decreased brush thickness, while Cl^–^ increased brush thickness.

Of note, comparisons of ion-modulated brush swelling across all examined solvent compositions yielded a þ value, which imparted no change to the polymer brush swelling or thermotransition. This proposed theoretical þ value was −5.2 × 10^–10^ C m^–1^ and represents the turning point in the direction of the influence (e.g., salting-out to salting-in), which transcends across solvent compositions and electrolyte strength. Further studies to investigate this transitional þ value are of great interest as spectator ions, which do not alter the system’s observed behavior, are often required, notably in buffers and biological systems.

## Supporting Information Available

Relevant data and code required to reproduce the analyses presented are readily available on Zenodo at 10.5281/zenodo.8248060. The Supporting Information is available free of charge at https://pubs.acs.org/doi/10.1021/acs.langmuir.3c02596.

þ values; additional spectroscopic ellipsometry; NR data; and NR PT-MCMC spread of fits (PDF)

## References

[ref1] Lutz J.-F.; Akdemir O.; Hoth A. Point by point comparison of two thermosensitive polymers exhibiting a similar LCST: Is the age of poly(NIPAM) over?. J. Am. Chem. Soc. 2006, 128, 13046–13047. 10.1021/ja065324n. 17017772

[ref2] Ishida N.; Biggs S. Effect of grafting density on phase transition behavior for poly(N-isopropylacryamide) brushes in aqueous solutions studied by AFM and QCM-D. Macromolecules 2010, 43, 7269–7276. 10.1021/ma101113g.

[ref3] Laloyaux X.; Mathy B.; Nysten B.; Jonas A. M. Surface and bulk collapse transitions of thermoresponsive polymer brushes. Langmuir 2010, 26, 838–847. 10.1021/la902285t. 19842635

[ref4] Yamamoto S. I.; Pietrasik J.; Matyjaszewski K. Temperature- and pH-responsive dense copolymer brushes prepared by ATRP. Macromolecules 2008, 41, 7013–7020. 10.1021/ma8011366.

[ref5] Willott J. D.; Murdoch T. J.; Webber G. B.; Wanless E. J. Physicochemical behaviour of cationic polyelectrolyte brushes. Prog. Polym. Sci. 2017, 64, 52–75. 10.1016/j.progpolymsci.2016.09.010.

[ref6] Robertson H.; Nelson A. R. J.; Prescott S. W.; Webber G. B.; Wanless E. J. Cosolvent effects on the structure and thermoresponse of a polymer brush: PNIPAM in DMSO–water mixtures. Polym. Chem. 2023, 14, 1526–1535. 10.1039/D2PY01487D.

[ref7] Robertson H.; Johnson E. C.; Gresham I. J.; Prescott S. W.; Nelson A.; Wanless E. J.; Webber G. B. Competitive specific ion effects in mixed salt solutions on a thermoresponsive polymer brush. J. Colloid Interface Sci. 2021, 586, 292–304. 10.1016/j.jcis.2020.10.092. 33189318

[ref8] Murdoch T. J.; Humphreys B. A.; Willott J. D.; Gregory K. P.; Prescott S. W.; Nelson A.; Wanless E. J.; Webber G. B. Specific Anion Effects on the Internal Structure of a Poly(N-isopropylacrylamide) Brush. Macromolecules 2016, 49, 6050–6060. 10.1021/acs.macromol.6b01001.

[ref9] Humphreys B. A.; Willott J. D.; Murdoch T. J.; Webber G. B.; Wanless E. J. Specific ion modulated thermoresponse of poly(N-isopropylacrylamide) brushes. Phys. Chem. Chem. Phys. 2016, 18, 6037–6046. 10.1039/C5CP07468A. 26840183

[ref10] Humphreys B. A.; Wanless E. J.; Webber G. B. Effect of ionic strength and salt identity on poly(N-isopropylacrylamide) brush modified colloidal silica particles. J. Colloid Interface Sci. 2018, 516, 153–161. 10.1016/j.jcis.2018.01.058. 29367066

[ref11] Freitag R.; Garret-Flaudy F. Salt effects on the thermoprecipitation of poly-(N-isopropylacrylamide) oligomers from aqueous solution. Langmuir 2002, 18, 3434–3440. 10.1021/la0106440.

[ref12] Zhang Y.; Furyk S.; Sagle L. B.; Cho Y.; Bergbreiter D. E.; Cremer P. S. Effects of Hofmeister anions on the LCST of PNIPAM as a function of molecular weight. J. Phys. Chem. C 2007, 111, 8916–8924. 10.1021/jp0690603. PMC2553222 18820735

[ref13] Zajforoushan Moghaddam S.; Thormann E. Hofmeister effect of salt mixtures on thermo-responsive poly(propylene oxide). Phys. Chem. Chem. Phys. 2015, 17, 6359–6366. 10.1039/C4CP05677A. 25648868

[ref14] Chen W. L.; Cordero R.; Tran H.; Ober C. K. 50th Anniversary Perspective: Polymer Brushes: Novel Surfaces for Future Materials. Macromolecules 2017, 50, 4089–4113. 10.1021/acs.macromol.7b00450.

[ref15] Açarı İ. K.; Sel E.; Özcan İ.; Ateş B.; Köytepe S.; Thakur V. K. Chemistry and engineering of brush type polymers: Perspective towards tissue engineering. Adv. Colloid Interface Sci. 2022, 305, 102694 10.1016/j.cis.2022.102694. 35597039

[ref16] Wang R.; Wei Q.; Sheng W.; Yu B.; Zhou F.; Li B. Driving Polymer Brushes from Synthesis to Functioning. Angew. Chem., Int. Ed. 2023, 62, 202219312 10.1002/anie.202219312. 36950880

[ref17] Ritsema Van Eck G. C.; Chiappisi L.; De Beer S. Fundamentals and Applications of Polymer Brushes in Air. ACS Appl. Polym. Mater. 2022, 4, 3062–3087. 10.1021/acsapm.1c01615. 35601464 PMC9112284

[ref18] Robertson H.; Willott J. D.; Gregory K. P.; Johnson E. C.; Gresham I. J.; Nelson A. R. J.; Craig V. S. J.; Prescott S. W.; Chapman R.; Webber G. B.; Wanless E. J. From Hofmeister to hydrotrope: Effect of anion hydrocarbon chain length on a polymer brush. J. Colloid Interface Sci. 2023, 634, 983–994. 10.1016/j.jcis.2022.12.114. 36571860

[ref19] Gregory K. P.; Elliott G. R.; Robertson H.; Kumar A.; Wanless E. J.; Webber G. B.; Craig V. S. J.; Andersson G. G.; Page A. J. Understanding specific ion effects and the Hofmeister series. Phys. Chem. Chem. Phys. 2022, 24, 12682–12718. 10.1039/D2CP00847E. 35543205

[ref20] Zhang F.; Skoda M. W.; Jacobs R. M.; Dressen D. G.; Martin R. A.; Martin C. M.; Clark G. F.; Lamkemeyer T.; Schreiber F. Gold nanoparticles decorated with oligo(ethylene glycol) thiols: Enhanced hofmeister effects in colloid-protein mixtures. J. Phys. Chem. C 2009, 113, 4839–4847. 10.1021/jp810869h.

[ref21] Lõpez-Leõn T.; Ortega-Vinuesa J. L.; Bastos-González D. Ion-specific aggregation of hydrophobic particles. ChemPhysChem 2012, 13, 2382–2391. 10.1002/cphc.201200120. 22556130

[ref22] Lo Nostro P.; Ninham B. W. Hofmeister Phenomena: An Update on Ion Specificity in Biology. Chem. Rev. 2012, 112, 2286–2322. 10.1021/cr200271j. 22251403

[ref23] Jenkins H. D. B.; Marcus Y. Viscosity B-Coefficients of Ions in Solution. Chem. Rev. 1995, 95, 2695–2724. 10.1021/cr00040a004.

[ref24] Pegram L. M.; Record M. T. Hofmeister salt effects on surface tension arise from partitioning of anions and cations between bulk water and the air-water interface. J. Phys. Chem. B 2007, 111, 5411–5417. 10.1021/jp070245z. 17432897

[ref25] Kunz W.; Henle J.; Ninham B. W. “Zur Lehre von der Wirkung der Salze” (about the science of the effect of salts): Franz Hofmeister’s historical papers. Curr. Opin. Colloid Interface Sci. 2004, 9, 19–37. 10.1016/j.cocis.2004.05.005.

[ref26] Collins K. D. Ions from the Hofmeister series and osmolytes: Effects on proteins in solution and in the crystallization process. Methods 2004, 34, 300–311. 10.1016/j.ymeth.2004.03.021. 15325648

[ref27] Collins K. D.; Washabaugh M. W. The Hofmeister effect and the behaviour of water at interfaces. Q. Rev. Biophys. 1985, 18, 323–422. 10.1017/S0033583500005369. 3916340

[ref28] Hamaguchi K.; Geiduschek E. P. The Effect of Electrolytes on the Stability of the Deoxyribonucleate Helix. J. Am. Chem. Soc. 1962, 84, 1329–1338. 10.1021/ja00867a001.

[ref29] Gregory K. P.; Webber G. B.; Wanless E. J.; Page A. J. Lewis Strength Determines Specific-Ion Effects in Aqueous and Nonaqueous Solvents. J. Phys. Chem. A 2019, 123, 6420–6429. 10.1021/acs.jpca.9b04004. 31314519

[ref30] Gregory K. P.; Wanless E. J.; Webber G. B.; Craig V. S.; Page A. J. The electrostatic origins of specific ion effects: Quantifying the Hofmeister series for anions. Chem. Sci. 2021, 12, 15007–15015. 10.1039/D1SC03568A. 34976339 PMC8612401

[ref31] Zhang Y.; Furyk S.; Bergbreiter D. E.; Cremer P. S. Specific ion effects on the water solubility of macromolecules: PNIPAM and the Hofmeister series. J. Am. Chem. Soc. 2005, 127, 14505–14510. 10.1021/ja0546424. 16218647

[ref32] Magnusson J. P.; Khan A.; Pasparakis G.; Saeed A. O.; Wang W.; Alexander C. Ion-sensitive “isothermal” responsive polymers prepared in water. J. Am. Chem. Soc. 2008, 130, 10852–10853. 10.1021/ja802609r. 18651734

[ref33] Mazzini V.; Liu G.; Craig V. S. Probing the Hofmeister series beyond water: Specific-ion effects in non-aqueous solvents. J. Chem. Phys. 2018, 148, 222805 10.1063/1.5017278. 29907022

[ref34] Mazzini V.; Craig V. S. Specific-ion effects in non-aqueous systems. Curr. Opin. Colloid Interface Sci. 2016, 23, 82–93. 10.1016/j.cocis.2016.06.009.

[ref35] Lovelock J. E.; Bishop M. W. Prevention of Freezing Damage to Living Cells by Dimethyl Sulphoxide. Nature 1959, 183, 1394–1395. 10.1038/1831394a0. 13657132

[ref36] Zheng Y. J.; Ornstein R. L. A molecular dynamics and quantum mechanics analysis of the effect of DMSO on enzyme structure and dynamics: Subtilisin. J. Am. Chem. Soc. 1996, 118, 4175–4180. 10.1021/ja9539195.

[ref37] Anchordoguy T. J.; Cecchini C. A.; Crowe J. H.; Crowe L. M. Insights into the cryoprotective mechanism of dimethyl sulfoxide for phospholipid bilayers. Cryobiology 1991, 28, 467–473. 10.1016/0011-2240(91)90056-T. 1752134

[ref38] Wohnhaas C. T.; Leparc G. G.; Fernandez-Albert F.; Kind D.; Gantner F.; Viollet C.; Hildebrandt T.; Baum P. DMSO cryopreservation is the method of choice to preserve cells for droplet-based single-cell RNA sequencing. Sci. Rep. 2019, 9, 10699 10.1038/s41598-019-46932-z. 31337793 PMC6650608

[ref39] Colucci M.; Maione F.; Bonito M. C.; Piscopo A.; Di Giannuario A.; Pieretti S. New insights of dimethyl sulphoxide effects (DMSO) on experimental in vivo models of nociception and inflammation. Pharmacol. Res. 2008, 57, 419–425. 10.1016/j.phrs.2008.04.004. 18508278

[ref40] Yamauchi H.; Maeda Y. LCST and UCST behavior of poly(N-isopropylacrylamide) in DMSO/water mixed solvents studied by IR and micro-raman spectroscopy. J. Phys. Chem. B 2007, 111, 12964–12968. 10.1021/jp072438s. 17949072

[ref41] Costa R. O.; Freitas R. F. Phase behavior of poly (N-isopropylacrylamide) in binary aqueous solutions. Polymer 2002, 43, 5879–5885. 10.1016/S0032-3861(02)00507-4.

[ref42] Liu L.; Wang T.; Liu C.; Lin K.; Liu G.; Zhang G. Specific anion effect in water-nonaqueous solvent mixtures: Interplay of the interactions between anion, solvent, and polymer. J. Phys. Chem. B 2013, 117, 10936–10943. 10.1021/jp406215c. 23980605

[ref43] Zhu R.; Baraniak M. K.; Jäkle F.; Liu G. Anion Specificity in Dimethyl Sulfoxide-Water Mixtures Exemplified by a Thermosensitive Polymer. J. Phys. Chem. B 2018, 122, 8293–8300. 10.1021/acs.jpcb.8b06125. 30086631

[ref44] Robertson H.; Gresham I. J.; Prescott S. W.; Webber G. B.; Wanless E. J.; Nelson A. *refellips*: A Python package for the analysis of variable angle spectroscopic ellipsometry data. SoftwareX 2022, 20, 101225 10.1016/j.softx.2022.101225.

[ref45] Lovell M. R.; Richardson R. M. Analysis methods in neutron and X-ray reflectometry. Curr. Opin. Colloid Interface Sci. 1999, 4, 197–204. 10.1016/S1359-0294(99)00039-4.

[ref46] Gresham I. J.; Murdoch T. J.; Johnson E. C.; Robertson H.; Webber G. B.; Wanless E. J.; Prescott S. W.; Nelson A. R. J. Quantifying the robustness of the neutron reflectometry technique for structural characterization of polymer brushes. J. Appl. Crystallogr. 2021, 54, 739–750. 10.1107/S160057672100251X.

[ref47] Gresham I. J.; Johnson E. C.; Robertson H.; Willott J. D.; Webber G. B.; Wanless E. J.; Nelson A. R. J.; Prescott S. W. Comparing polymer-surfactant complexes to polyelectrolytes. J. Colloid Interface Sci. 2024, 655, 262–272. 10.1016/j.jcis.2023.10.101. 37944374

[ref48] Robertson H.; Gresham I. J.; Nelson A. R. J.; Gregory K. P.; Johnson E. C.; Willott J. D.; Prescott S. W.; Webber G. B.; Wanless E. J. Supporting Information for Solvent modulated specific ion effects: PNIPAM brushes in non-aqueous electrolytes, 2023. 10.5281/zenodo.8248060. PMC10910595 38117209

[ref49] James M.; Nelson A.; Holt S. A.; Saerbeck T.; Hamilton W. A.; Klose F. The multipurpose time-of-flight neutron reflectometer “Platypus” at Australias OPAL reactor. Nucl. Instrum. Methods Phys. Res., Sect. A 2011, 632, 112–123. 10.1016/j.nima.2010.12.075.

[ref50] Nelson A. J.; Prescott S. W. *refnx*: neutron and X-ray reflectometry analysis in Python. J. Appl. Crystallogr. 2019, 52, 193–200. 10.1107/s1600576718017296. 30800030 PMC6362611

[ref51] Halperin A.; Kröger M.; Winnik F. M. Poly(N-isopropylacrylamide) Phase Diagrams: Fifty Years of Research. Angew. Chem., Int. Ed. 2015, 54, 15342–15367. 10.1002/anie.201506663. 26612195

[ref52] Johnson E. C.; Gresham I. J.; Prescott S. W.; Nelson A.; Wanless E. J.; Webber G. B. The direction of influence of specific ion effects on a pH and temperature responsive copolymer brush is dependent on polymer charge. Polymer 2021, 214, 123287 10.1016/j.polymer.2020.123287.

