# Supplementary material for: Solvent-Modulated Specific Ion Effects: Poly(N-isopropylacrylamide) Brushes in Nonaqueous Electrolytes
Source: Langmuir. 2023 Dec 20;40(1):335–47. doi: 10.1021/acs.langmuir.3c02596 (PMC10910595; doi:10.1021/acs.langmuir.3c02596)
Supplement: Supplementary file 1 — la3c02596_si_001.pdf [file la3c02596_si_001.pdf]

SI for "Solvent modulated specific ion effects: PNIPAM brushes in non-aqueous electrolytes"

Hayden Robertson[a], Isaac J. Gresham[b], Andrew R. J. Nelson[c], Kasimir P. Gregory[d], Edwin C. Johnson[e], Joshua D. Willott[a], Stuart W. Prescott[f], Grant B. Webber[a], and Erica J. Wanless[a,*]

[a] *College of Science, Engineering and Environment, University of Newcastle, Callaghan, Australia*
[b] *School of Chemistry, University of Sydney, Sydney, Australia*
[c] *Australian Centre for Neutron Scattering, ANSTO, Locked Bag 2001, Kirrawee DC, NSW 2232, Australia*
[d] *Department of Materials Physics, Research School of Physics, Australian National University, Canberra, ACT 0200, Australia*
[e] *Department of Chemistry, The University of Sheffield, Sheffield, UK*
[f] *School of Chemical Engineering, UNSW Sydney, NSW 2052, Australia*
[*] *email: erica.wanless@newcastle.edu.au*

All relevant data and code required to reproduce the analyses presented are readily available on Zenodo at `https://doi.org/10.5281/zenodo.8248060`.

Contents

1 þ values **2**

2 Ellipsometry **2**
2.1 Ellipsometry temperature ramps thin brush 2
2.2 Ellipsometry temperature ramps thick brush 3

3 Additional NR polymer VF profiles **6**
3.1 $x_D = 0$ 6
3.2 $x_D = 0.06$ 7
3.3 $x_D = 0.70$ 8

4 NR PT-MCMC Spread of fits **9**
4.1 D_2O 9
4.1.1 198 Å PNIPAM brush 9
4.1.2 247 Å PNIPAM brush 16
4.2 6 mol% DMSO 21
4.2.1 198 Å PNIPAM brush 21
4.2.2 247 Å PNIPAM brush 27
4.3 70 mol% DMSO 32
4.3.1 244 Å PNIPAM brush 32
4.4 100 mol% DMSO 45
4.4.1 198 Å PNIPAM brush 45

References **47**

1 þ values

Ion identity	þ, C·m^{-1}
Cl^-	-6.25×10^{-10}
Br^-	-5.63×10^{-10}
I^-	-4.90×10^{-10}
SCN^-	-4.40×10^{-10}
K^+	9.24×10^{-10}
Li^+	31.61×10^{-10}

Table S1.1: Relevant þ values for this work. All values are taken from the work of Gregory *et al.*[1]

2 Ellipsometry

All ellipsometry data pertaining to the ‘thin’ brush was modelled with uniform layers (slabs), whereas the diffuse nature of the ‘thick’ brush modelled with a Piecewise Cubic Hermite Interpolating Polynomial. The temperature-modulated behaviour of the ‘thick’ PNIPAM brush in pure water as monitored by spectroscopic ellipsometry is presented in Figure S2.1, which illustrates the polymer VF profile of the brush at various temperatures, as well as the corresponding brush thickness. All data was fit using *refellips*.[2]

2.1 Ellipsometry temperature ramps thin brush

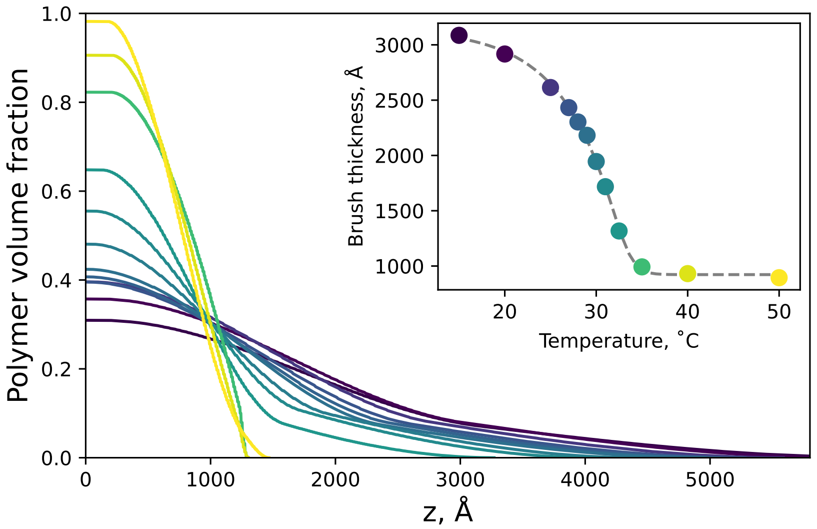

Figure S2.1: Spectroscopic ellipsometry of the 714 Å PNIPAM brush in water revealing polymer volume fraction as a function of distance from the substrate (z) and temperature. Inset presents the resultant brush thickness determined by extracting the first moment from each polymer VF profile *via* Equation 1. Dashed line in the inset shows the logistical model employed to extract an LCST of 32.2 °C *via* Equation 2.

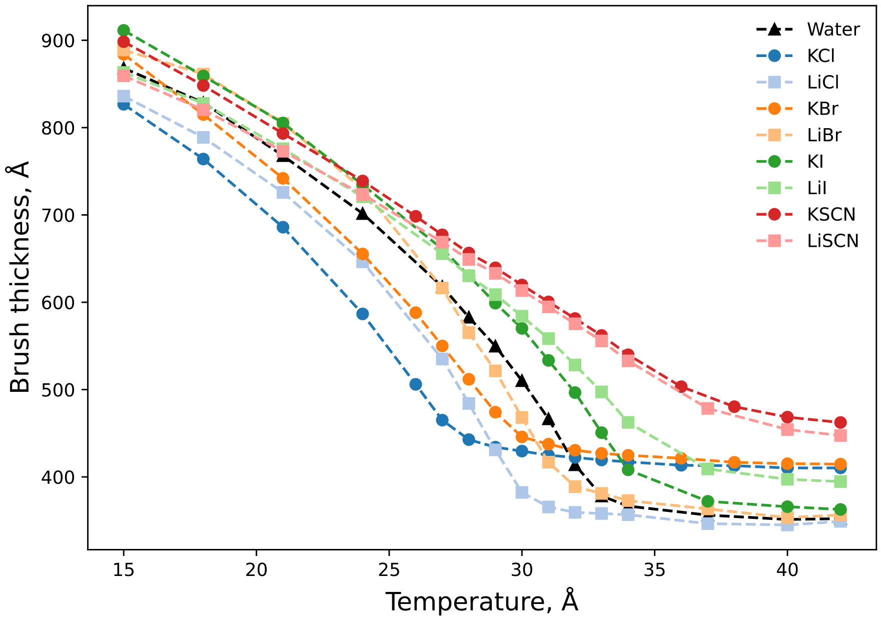

Figure S2.2: Brush thickness as a function of temperature and electrolyte identity for the thin (306 Å) PNIPAM brush in water. Electrolyte concentration was fixed at 0.9 mol%.

2.2 Ellipsometry temperature ramps thick brush

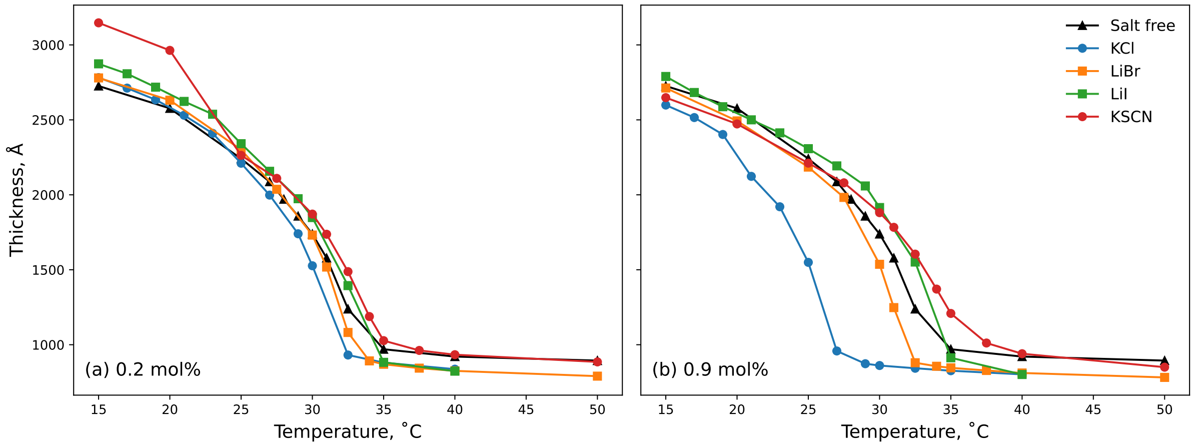

Figure S2.3: Brush thickness as a function of temperature and electrolyte identity for the thick (714 Å) PNIPAM brush in water for (a) 0.2 mol% and (b) 0.9 mol% electrolyte concentrations.

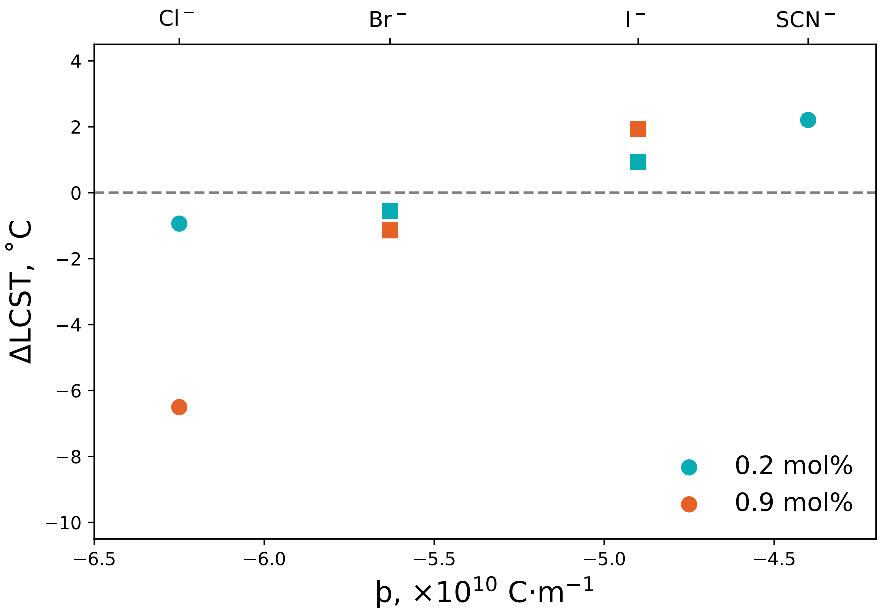

Figure S2.4: Change in LCST, relative to the thermotransition in pure water (LCST = 33.6 °C), for the thick (714 Å) PNIPAM brush in 0.2 mol% and 0.9 mol% aqueous electrolytes as a function of anion þ. Square symbols represent a Li^+ counter-cation and circle symbols a K^+ counter-cation. Top x-axis illustrates ions probed and lower x-axis shows their respective þ value.

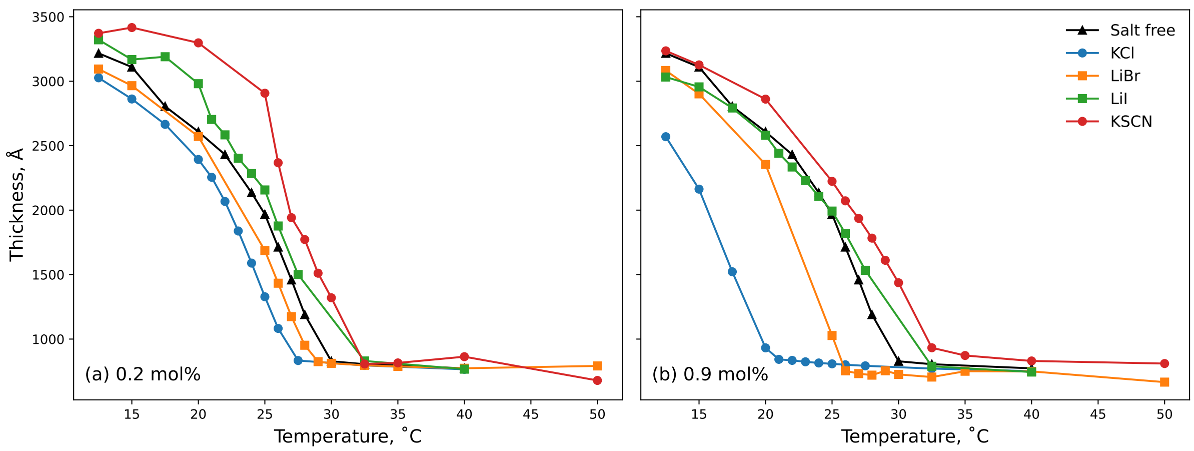

Figure S2.5: Brush thickness as a function of temperature and electrolyte identity for the thick (714 Å) PNIPAM brush in 6 mol% DMSO for (a) 0.2 mol% and (b) 0.9 mol% electrolyte concentrations.

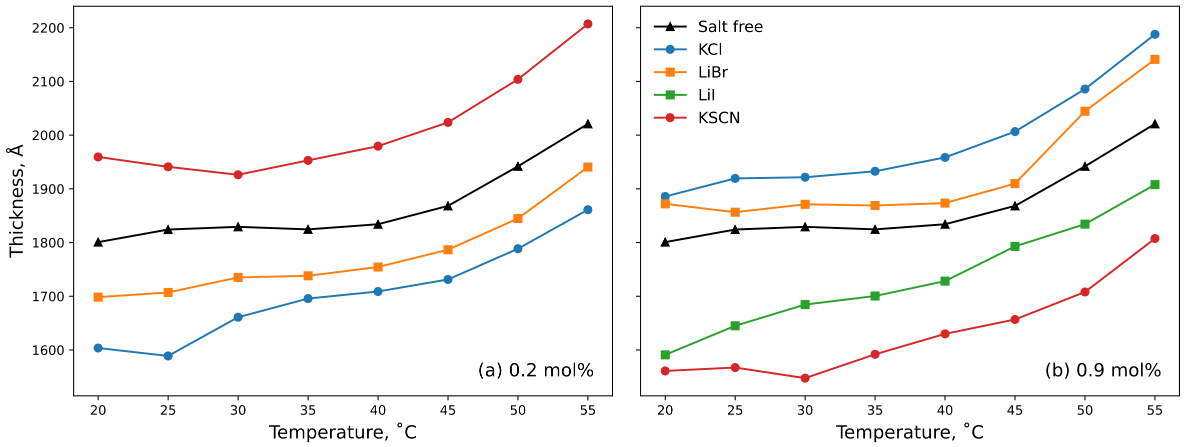

Figure S2.6: Brush thickness as a function of temperature and electrolyte identity for the thick (714 Å) PNIPAM brush in 70 mol% DMSO for (a) 0.2 mol% and (b) 0.9 mol% electrolyte concentrations.

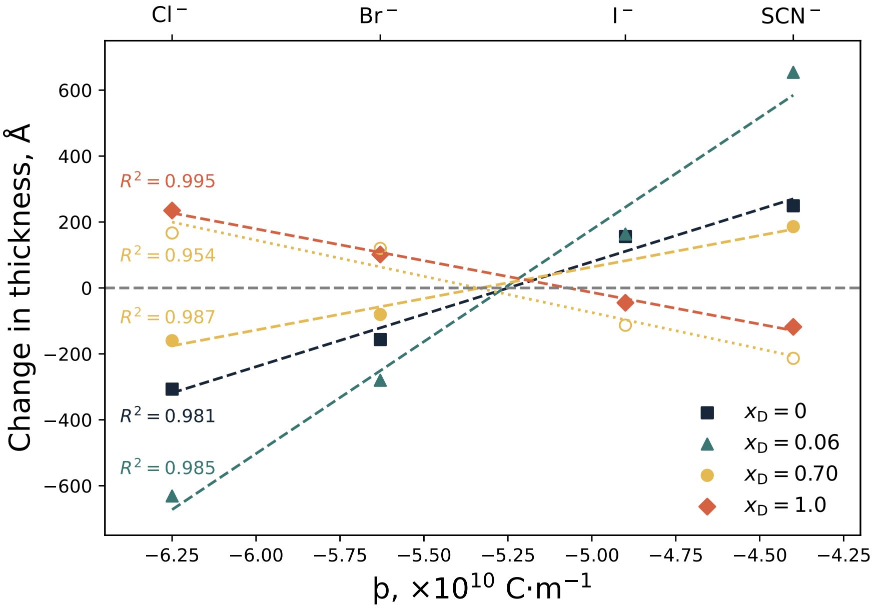

Figure S2.7: Changes in film thickness from ellipsometry of a 714 Å PNIPAM brush in various solvent compositions and electrolyte identities: KCl, LiBr, LiI, and KSCN. The top x-axis illustrates the þ values of the anions probed. Filled symbols are 0.2 mol% and open symbols are 0.9 mol%. The change in thickness is relative to the 'no salt' condition for each respective solvent composition. The chosen temperature was constant for each solvent composition, and was selected to best represent the thermotransition region: 32.5 °C for water; 26 °C for 6 mol% DMSO; 55 °C for 70 mol% and 40 °C for pure DMSO.

3 Additional NR polymer VF profiles

All neutron reflectometry was modelled using *refnx* in concordance with our previous protocols.[3,4]

3.1 $x_{\mathrm{D}} = 0$

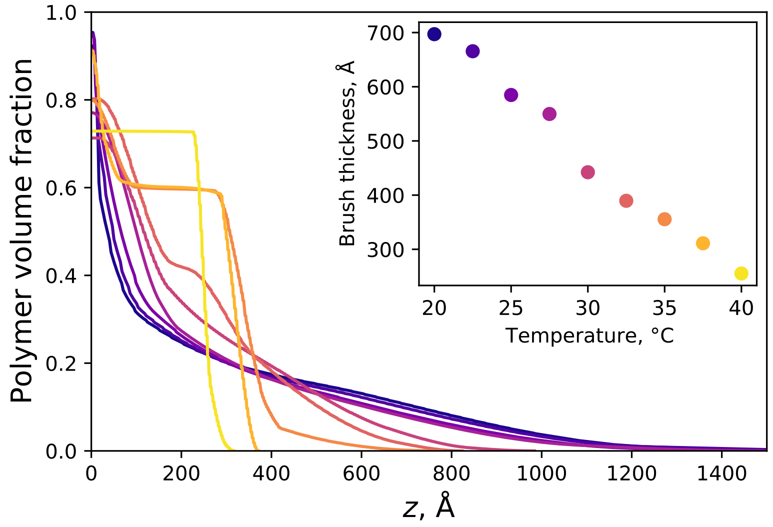

Figure S3.1: Neutron reflectometry derived polymer VF profile of the 198 Å PNIPAM brush in D_2O as a function of temperature. Inset presents the first moment derived brush thicknesses *via* Equation 1. Insufficient temperature precision is present to extract the LCST.

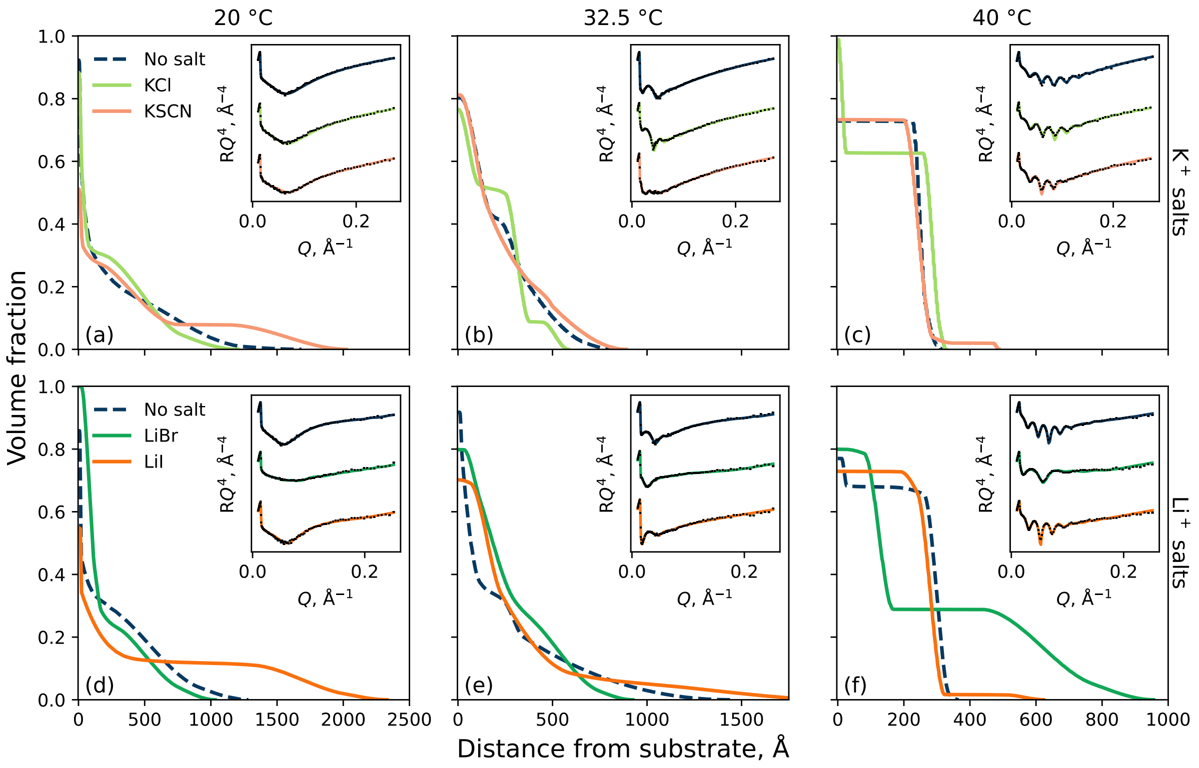

Figure S3.2: Neutron reflectometry determined polymer VF profiles of a (a,b,c) 198 Å PNIPAM brush in $x_{\mathrm{D}} = 0$ potassium electrolytes and a (d,e,f) 247 Å PNIPAM brush in lithium electrolytes at (a,d) 20 °C, (b,e) 32.5 °C, and (c,f) 40 °C. Insets present the measured reflectivity with superimposed optimised fits. Electrolyte concentration is fixed at 0.2 mol%.

3.2 $x_{\mathrm{D}} = 0.06$

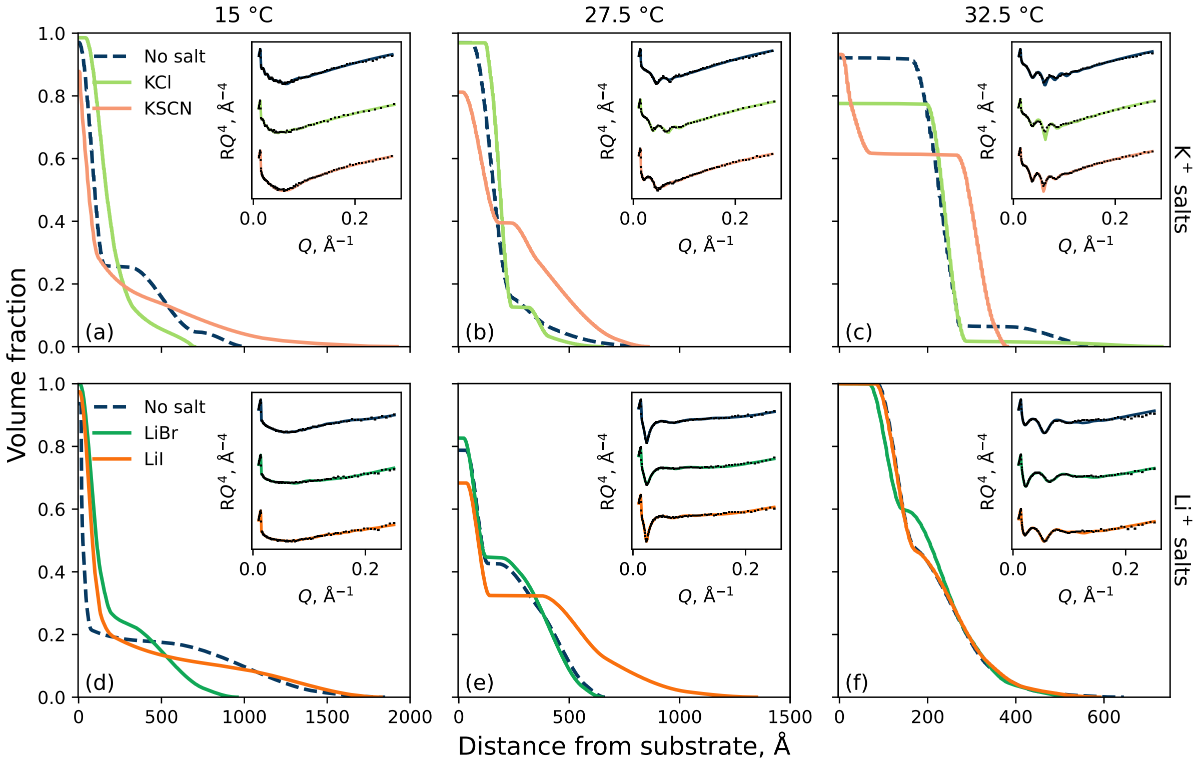

Figure S3.3: Polymer VF profiles derived by NR of a (a,b,c) 198 Å PNIPAM brush in $x_{\mathrm{D}} = 0.06$ potassium electrolytes and a (d,e,f) 247 Å PNIPAM brush in lithium electrolytes at (a,d) 15 °C, (b,e) 27.5 °C, and (c,f) 32.5 °C. Electrolyte concentration is fixed at 0.2 mol%. Insets present the measured reflectivity with superimposed optimised fits.

3.3 $x_{\mathrm{D}} = 0.70$

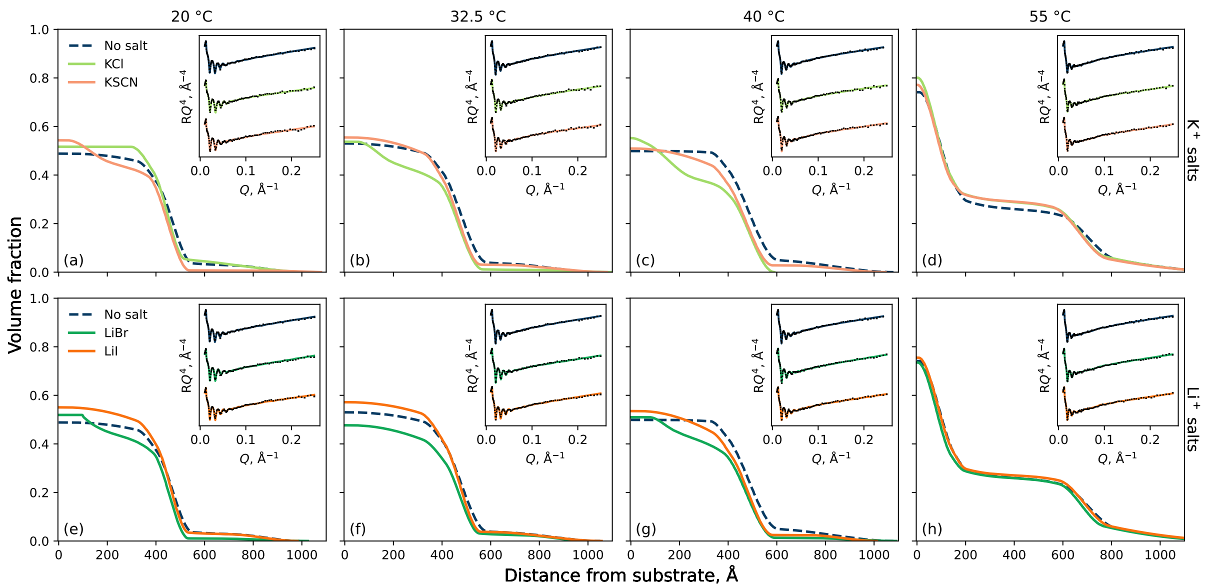

Figure S3.4: Polymer VF profiles derived by NR of a 244 Å PNIPAM brush in $x_{\mathrm{D}} = 0.70$ (a,b,c,d) potassium electrolytes and (e,f,g,h) lithium electrolytes at (a,e) 20 °C, (b,f) 32.5 °C, (c,g) 40 °C, and (d,h) 55 °C. Electrolyte concentration is fixed at 0.2 mol%. Insets present the measured reflectivity with superimposed optimised fits. The PT-MCMC derived spread of fits is presented in Figure SI.

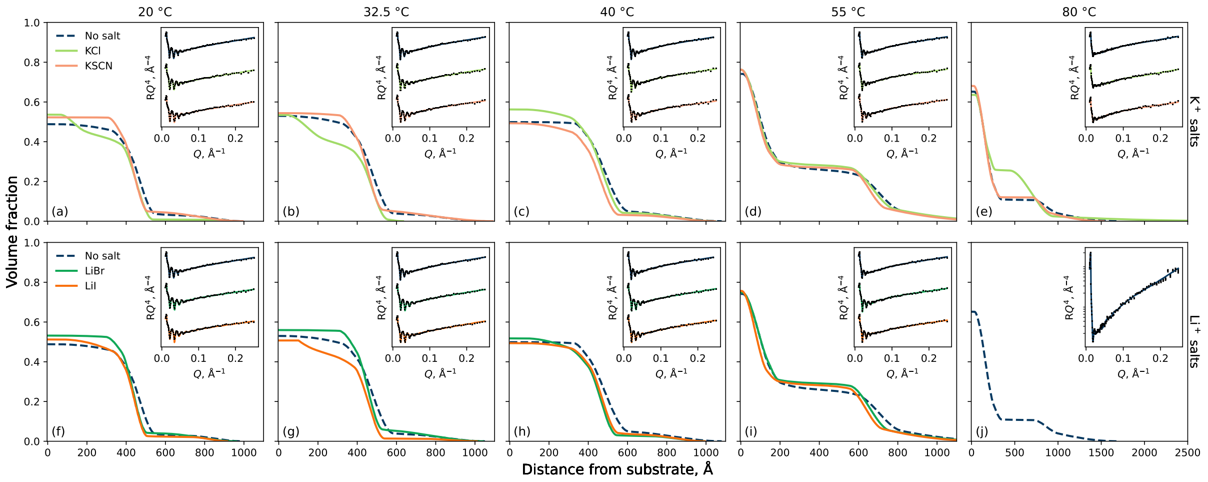

Figure S3.5: Polymer VF profiles derived by NR of a 244 Å PNIPAM brush in $x_{\mathrm{D}} = 0.70$ (a,b,c,d) potassium electrolytes and (e,f,g,h) lithium electrolytes at (a,e) 20 °C, (b,f) 32.5 °C, (c,g) 40 °C, and (d,h) 55 °C. Electrolyte concentration is fixed at 0.9 mol%. Insets present the measured reflectivity with superimposed optimised fits. The PT-MCMC derived spread of fits is presented in Figure SI.

4 NR PT-MCMC Spread of fits

4.1 D_2O

4.1.1 198 Å PNIPAM brush

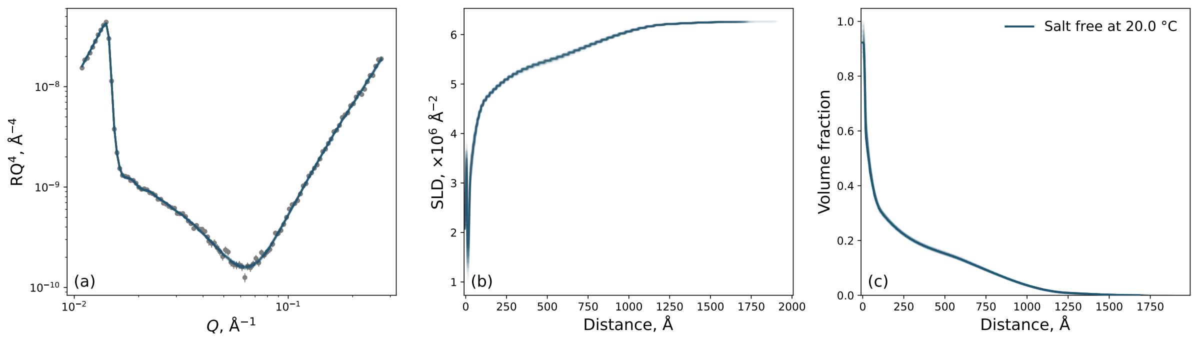

Figure S4.1.1i: (a) Reflectivity, (b) SLD and (c) polymer VF profiles of the 198 Å PNIPAM brush in $x_{\mathrm{D}} = 0$ at 20.0 °C with the superimposed distribution of fits from PT-MCMC sampling.

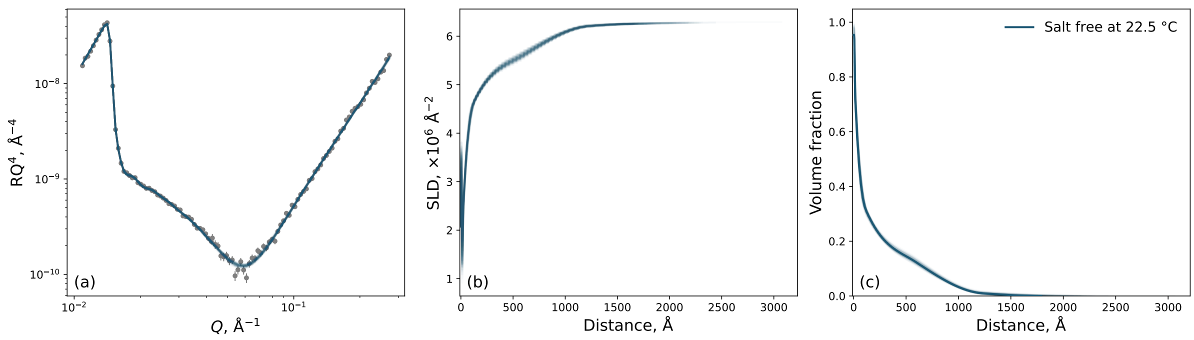

Figure S4.1.1ii: (a) Reflectivity, (b) SLD and (c) polymer VF profiles of the 198 Å PNIPAM brush in $x_{\mathrm{D}} = 0$ at 22.5 °C with the superimposed distribution of fits from PT-MCMC sampling.

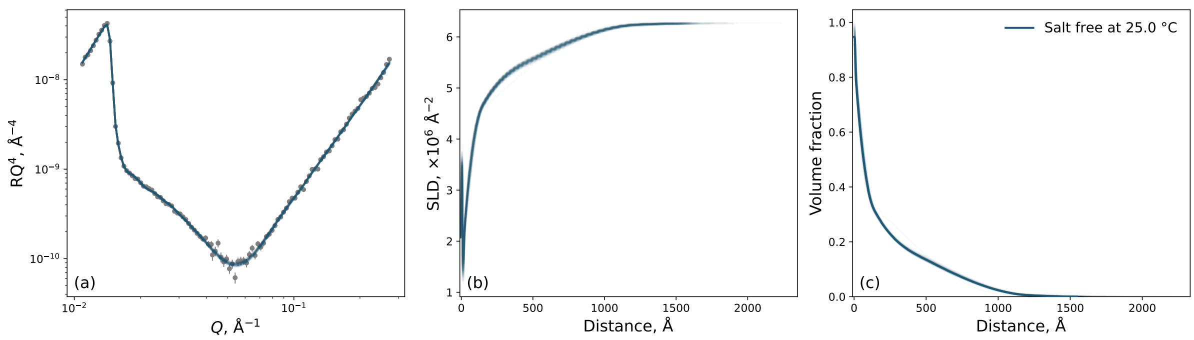

Figure S4.1.1iii: (a) Reflectivity, (b) SLD and (c) polymer VF profiles of the 198 Å PNIPAM brush in $x_{\mathrm{D}} = 0$ at 25.0 °C with the superimposed distribution of fits from PT-MCMC sampling.

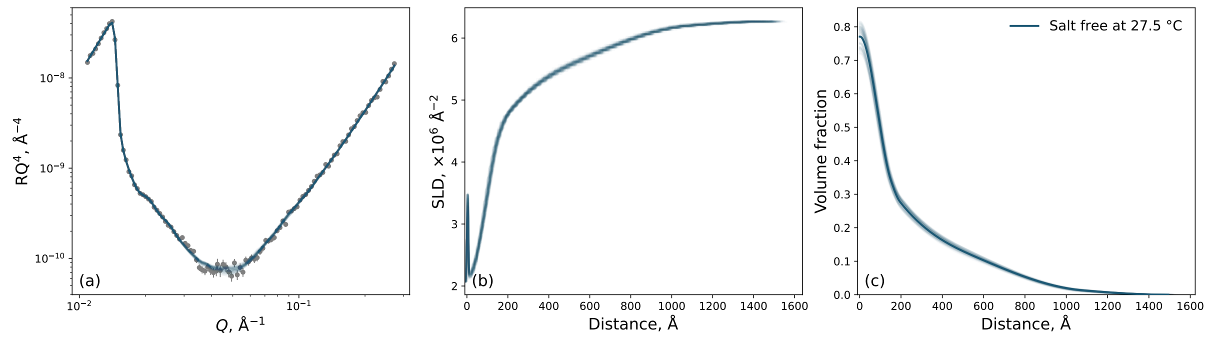

Figure S4.1.1iv: (a) Reflectivity, (b) SLD and (c) polymer VF profiles of the 198 Å PNIPAM brush in $x_{\mathrm{D}} = 0$ at 27.5 °C with the superimposed distribution of fits from PT-MCMC sampling.

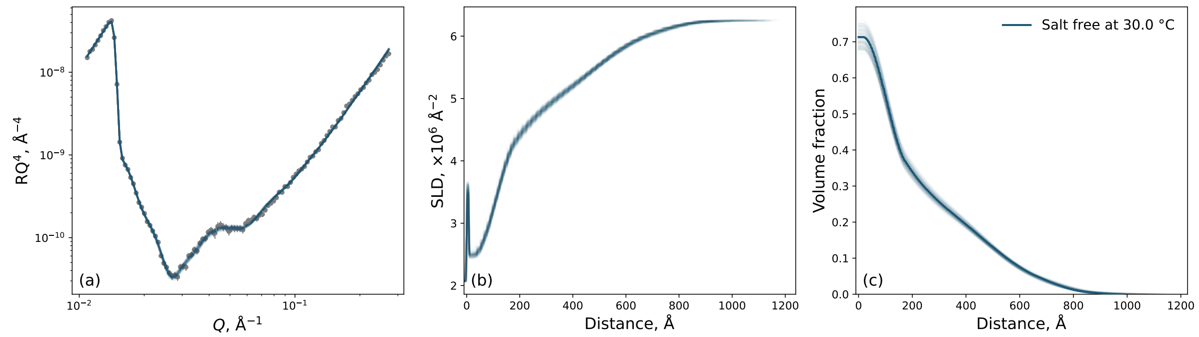

Figure S4.1.1v: (a) Reflectivity, (b) SLD and (c) polymer VF profiles of the 198 Å PNIPAM brush in $x_{\mathrm{D}} = 0$ at 30.0 °C with the superimposed distribution of fits from PT-MCMC sampling.

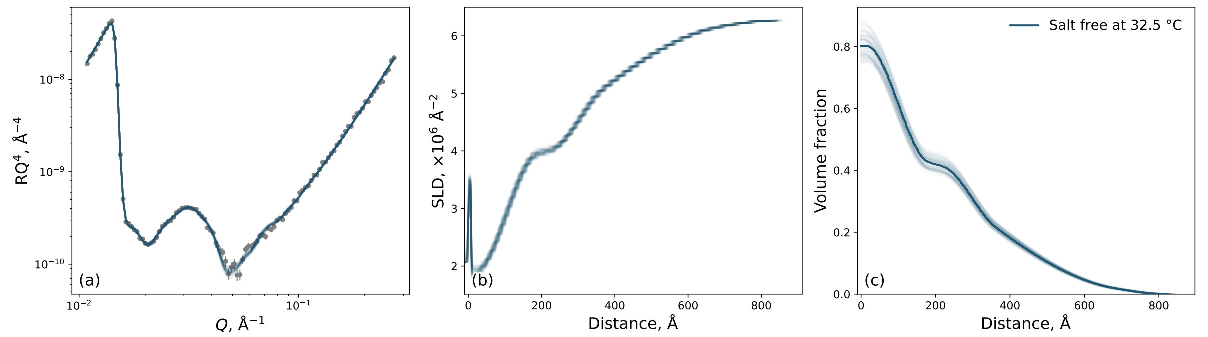

Figure S4.1.1vi: (a) Reflectivity, (b) SLD and (c) polymer VF profiles of the 198 Å PNIPAM brush in $x_{\mathrm{D}} = 0$ at 32.5 °C with the superimposed distribution of fits from PT-MCMC sampling.

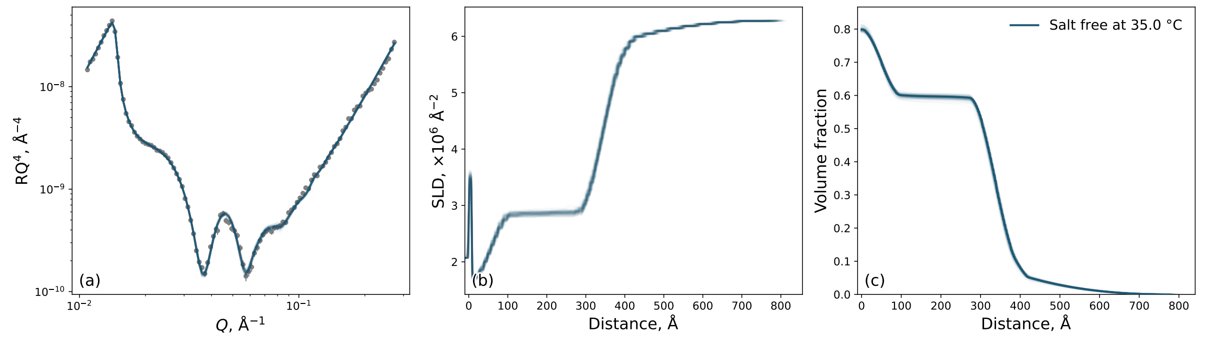

Figure S4.1.1vii: (a) Reflectivity, (b) SLD and (c) polymer VF profiles of the 198 Å PNIPAM brush in $x_{\mathrm{D}} = 0$ at 35.0 °C with the superimposed distribution of fits from PT-MCMC sampling.

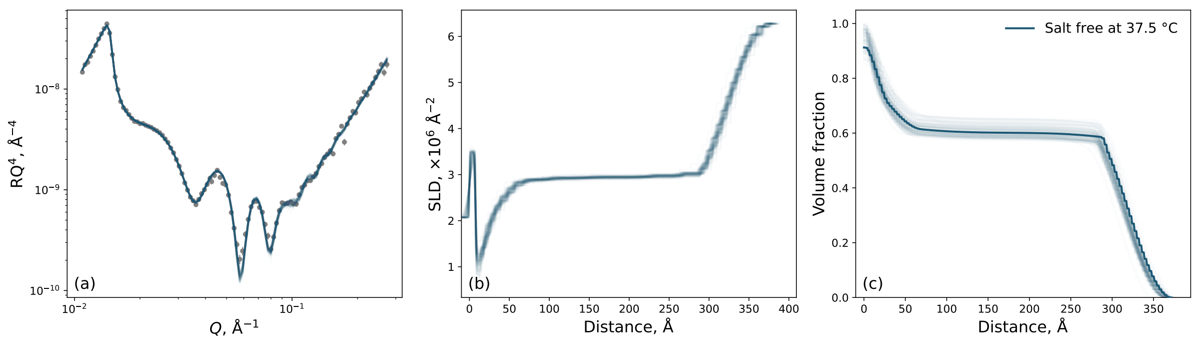

Figure S4.1.1viii: (a) Reflectivity, (b) SLD and (c) polymer VF profiles of the 198 Å PNIPAM brush in $x_{\mathrm{D}} = 0$ at 37.5 °C with the superimposed distribution of fits from PT-MCMC sampling.

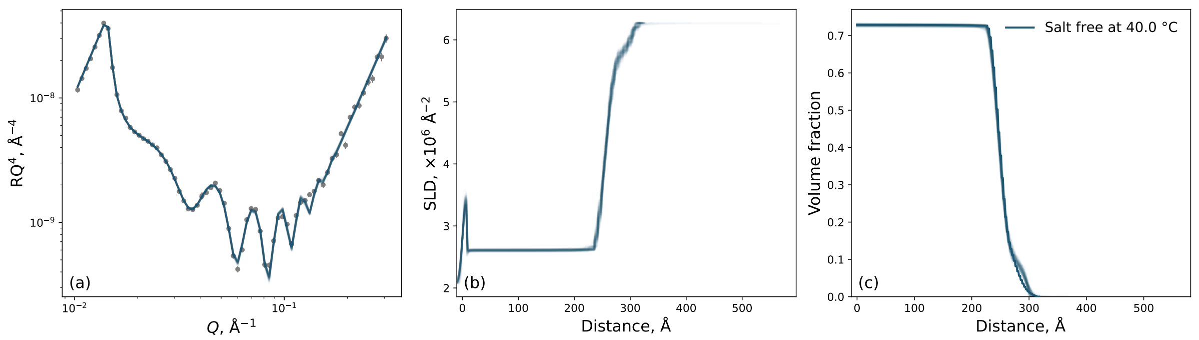

Figure S4.1.1ix: (a) Reflectivity, (b) SLD and (c) polymer VF profiles of the 198 Å PNIPAM brush in $x_{\mathrm{D}} = 0$ at 40.0 °C with the superimposed distribution of fits from PT-MCMC sampling.

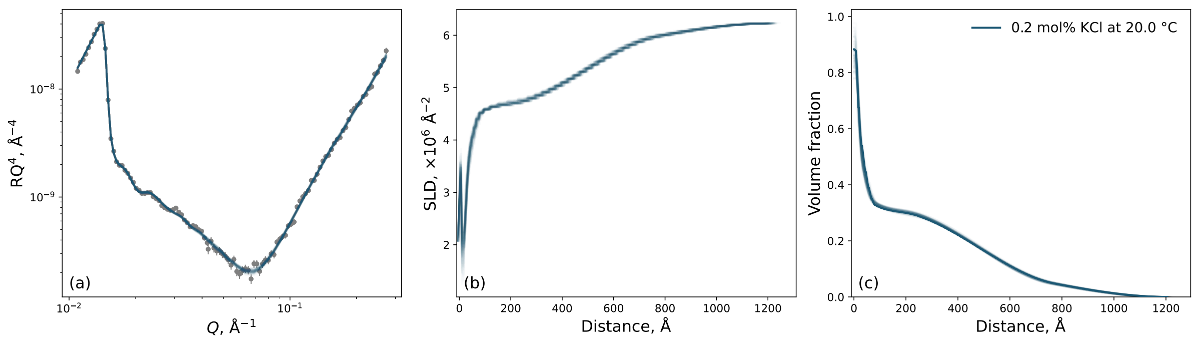

Figure S4.1.1x: (a) Reflectivity, (b) SLD and (c) polymer VF profiles of the 198 Å PNIPAM brush in a 0.2 mol% KCl electrolyte in $x_{\mathrm{D}} = 0$ at 20.0 °C with the superimposed distribution of fits from PT-MCMC sampling.

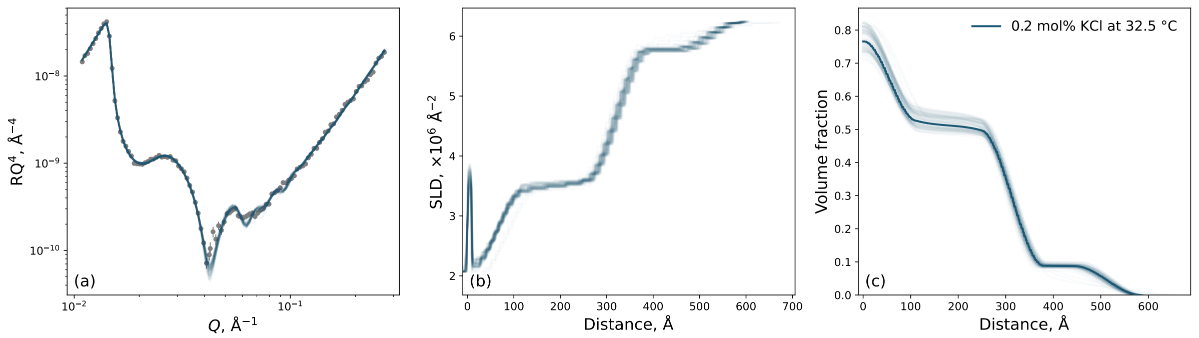

Figure S4.1.1xi: (a) Reflectivity, (b) SLD and (c) polymer VF profiles of the 198 Å PNIPAM brush in a 0.2 mol% KCl electrolyte in $x_\mathrm{D} = 0$ at 32.5 °C with the superimposed distribution of fits from PT-MCMC sampling.

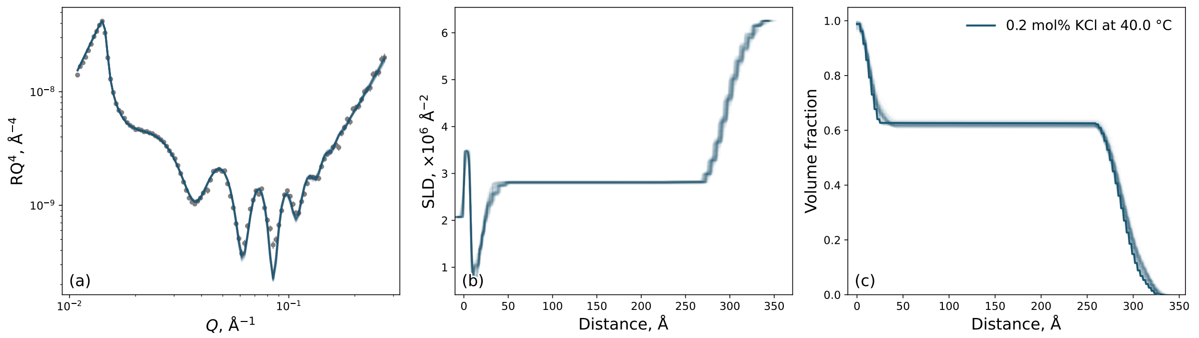

Figure S4.1.1xii: (a) Reflectivity, (b) SLD and (c) polymer VF profiles of the 198 Å PNIPAM brush in a 0.2 mol% KCl electrolyte in $x_\mathrm{D} = 0$ at 40.0 °C with the superimposed distribution of fits from PT-MCMC sampling.

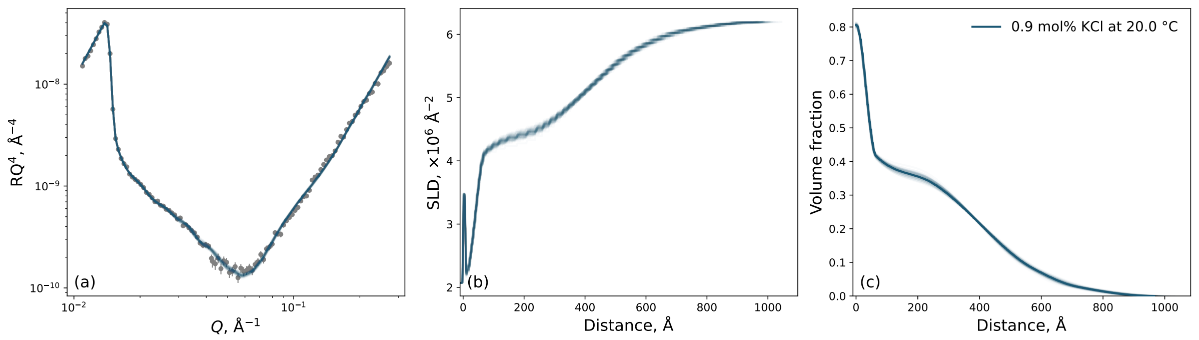

Figure S4.1.1xiii: (a) Reflectivity, (b) SLD and (c) polymer VF profiles of the 198 Å PNIPAM brush in a 0.9 mol% KCl electrolyte in $x_\mathrm{D} = 0$ at 20.0 °C with the superimposed distribution of fits from PT-MCMC sampling.

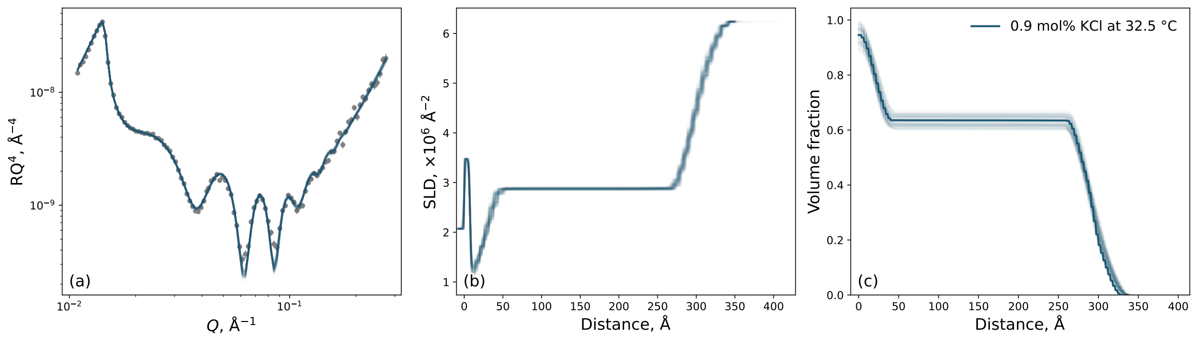

Figure S4.1.1xiv: (a) Reflectivity, (b) SLD and (c) polymer VF profiles of the 198 Å PNIPAM brush in a 0.9 mol% KCl electrolyte in $x_{\mathrm{D}} = 0$ at 32.5 °C with the superimposed distribution of fits from PT-MCMC sampling.

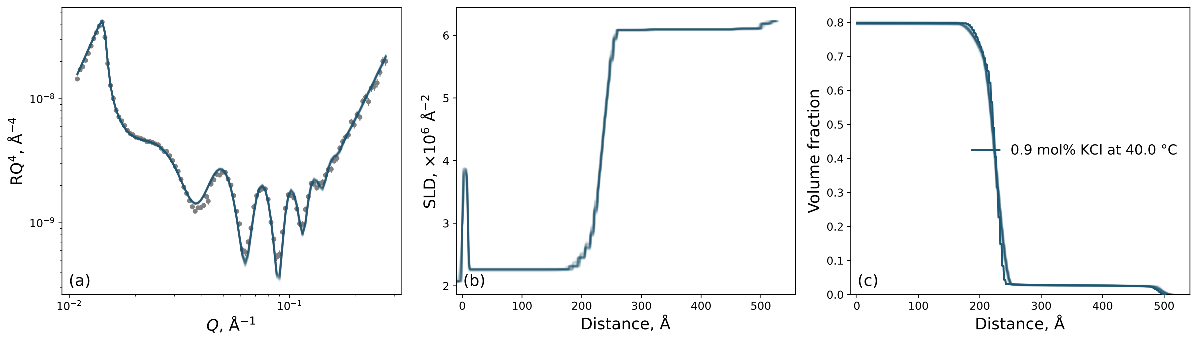

Figure S4.1.1xv: (a) Reflectivity, (b) SLD and (c) polymer VF profiles of the 198 Å PNIPAM brush in a 0.9 mol% KCl electrolyte in $x_{\mathrm{D}} = 0$ at 40.0 °C with the superimposed distribution of fits from PT-MCMC sampling.

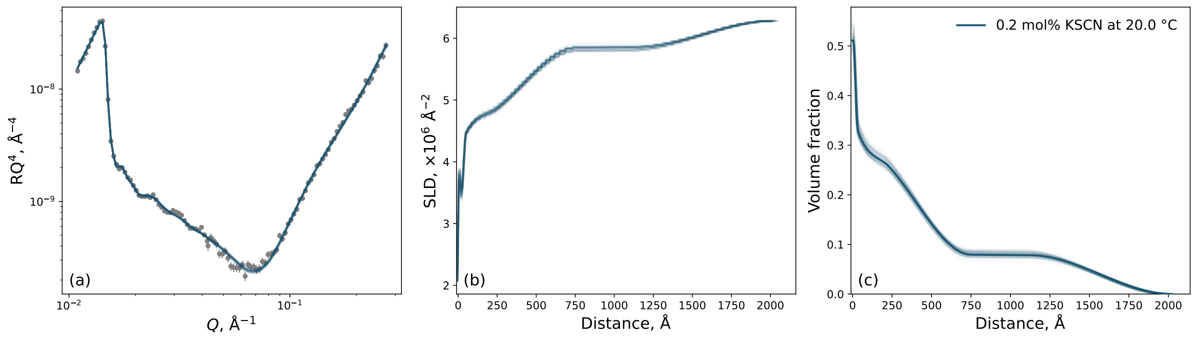

Figure S4.1.1xvi: (a) Reflectivity, (b) SLD and (c) polymer VF profiles of the 198 Å PNIPAM brush in a 0.2 mol% KSCN electrolyte in $x_{\mathrm{D}} = 0$ at 20.0 °C with the superimposed distribution of fits from PT-MCMC sampling.

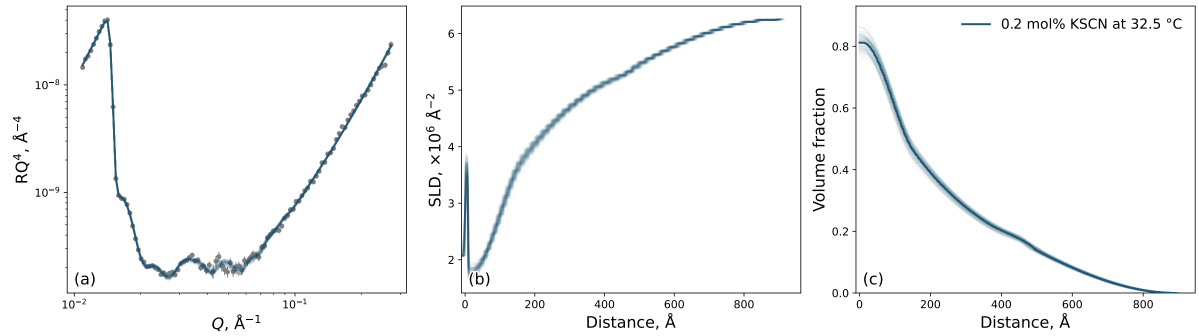

Figure S4.1.1xvii: (a) Reflectivity, (b) SLD and (c) polymer VF profiles of the 198 Å PNIPAM brush in a 0.2 mol% KSCN electrolyte in $x_{\mathrm{D}} = 0$ at 32.5 °C with the superimposed distribution of fits from PT-MCMC sampling.

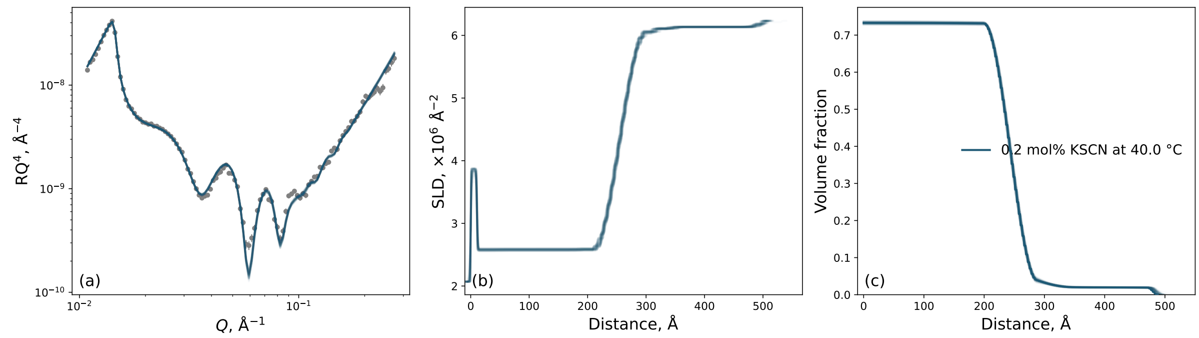

Figure S4.1.1xviii: (a) Reflectivity, (b) SLD and (c) polymer VF profiles of the 198 Å PNIPAM brush in a 0.2 mol% KSCN electrolyte in $x_{\mathrm{D}} = 0$ at 40.0 °C with the superimposed distribution of fits from PT-MCMC sampling.

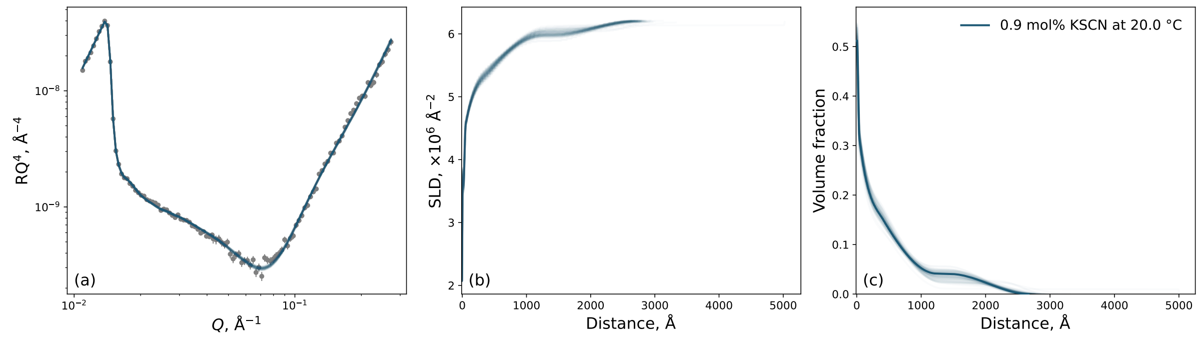

Figure S4.1.1xix: (a) Reflectivity, (b) SLD and (c) polymer VF profiles of the 198 Å PNIPAM brush in a 0.9 mol% KSCN electrolyte in $x_{\mathrm{D}} = 0$ at 20.0 °C with the superimposed distribution of fits from PT-MCMC sampling.

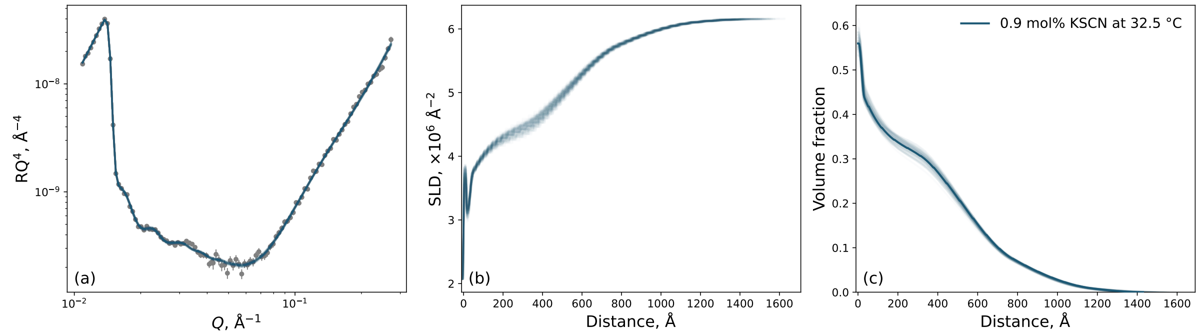

Figure S4.1.1xx: (a) Reflectivity, (b) SLD and (c) polymer VF profiles of the 198 Å PNIPAM brush in a 0.9 mol% KSCN electrolyte in $x_{\mathrm{D}} = 0$ at 32.5 °C with the superimposed distribution of fits from PT-MCMC sampling.

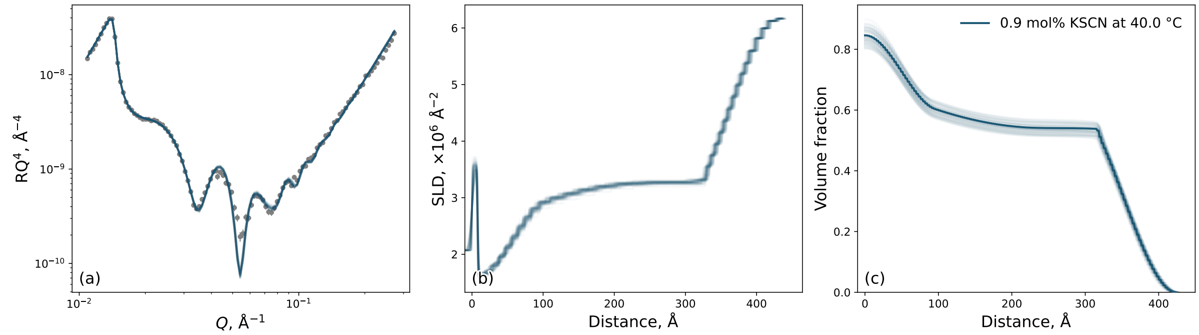

Figure S4.1.1xxi: (a) Reflectivity, (b) SLD and (c) polymer VF profiles of the 198 Å PNIPAM brush in a 0.9 mol% KSCN electrolyte in $x_{\mathrm{D}} = 0$ at 40.0 °C with the superimposed distribution of fits from PT-MCMC sampling.

4.1.2 247 Å PNIPAM brush

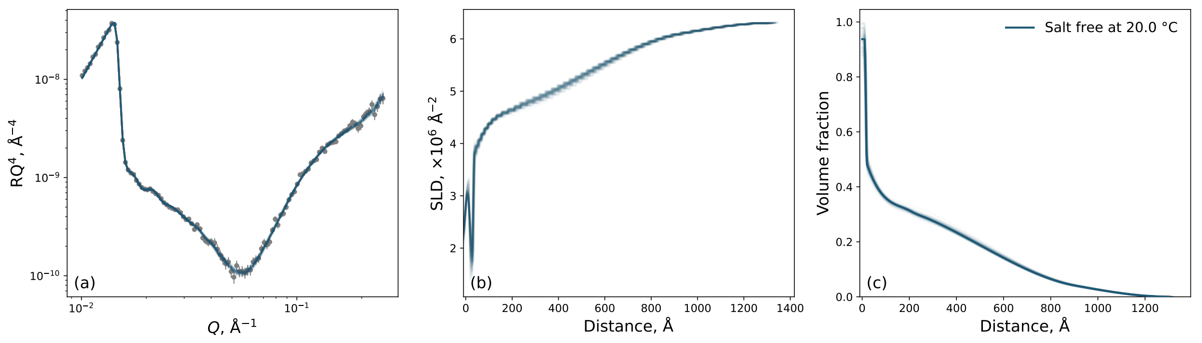

Figure S4.1.2i: (a) Reflectivity, (b) SLD and (c) polymer VF profiles of the 247 Å PNIPAM brush in $x_{\mathrm{D}} = 0$ at 20.0 °C with the superimposed distribution of fits from PT-MCMC sampling.

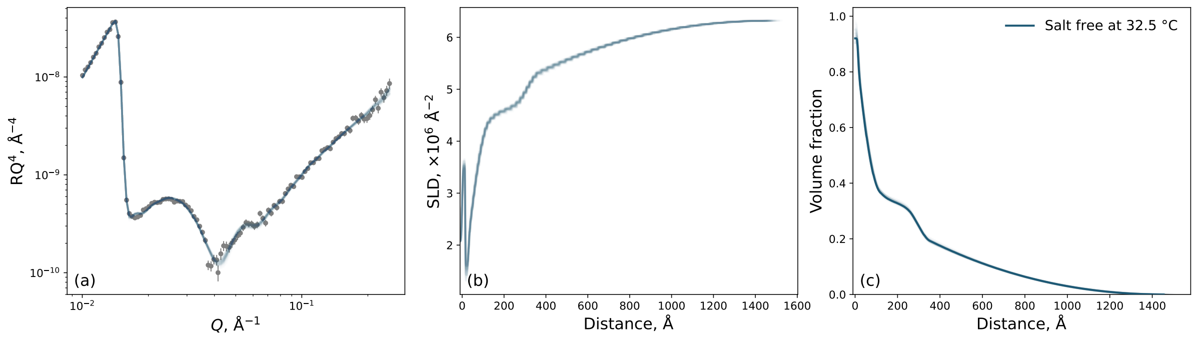

Figure S4.1.2ii: (a) Reflectivity, (b) SLD and (c) polymer VF profiles of the 247 Å PNIPAM brush in $x_{\mathrm{D}} = 0$ at 32.5 °C with the superimposed distribution of fits from PT-MCMC sampling.

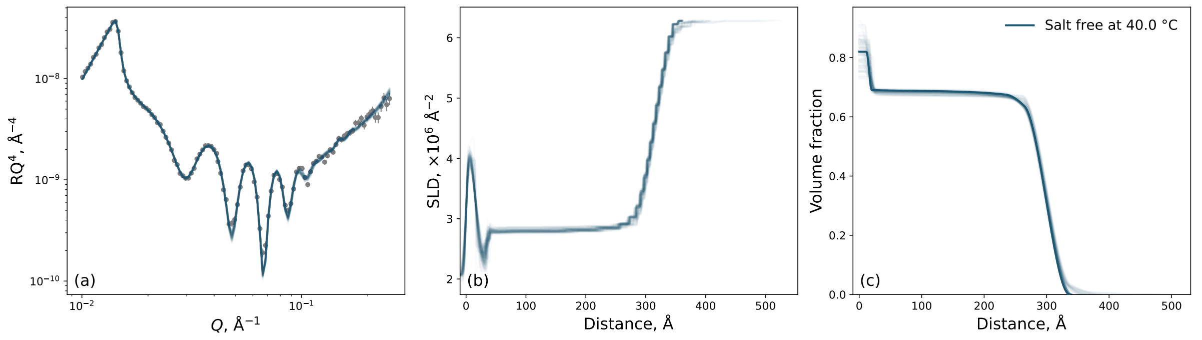

Figure S4.1.2iii: (a) Reflectivity, (b) SLD and (c) polymer VF profiles of the 247 Å PNIPAM brush in $x_{\mathrm{D}} = 0$ at 40.0 °C with the superimposed distribution of fits from PT-MCMC sampling.

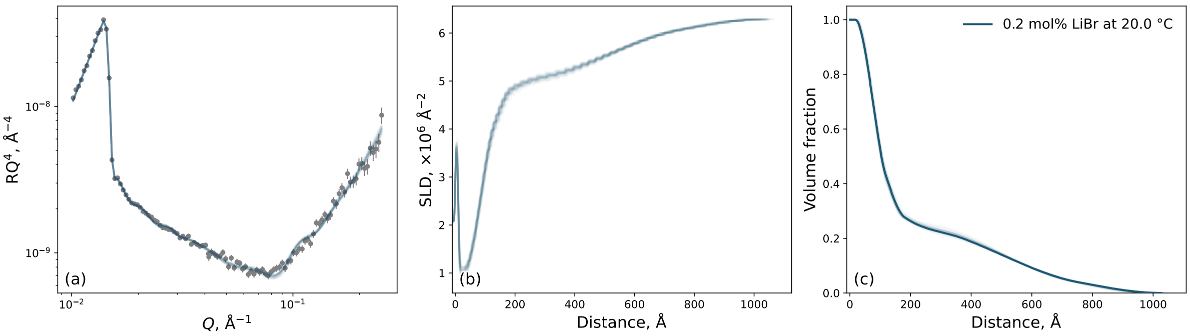

Figure S4.1.2iv: (a) Reflectivity, (b) SLD and (c) polymer VF profiles of the 247 Å PNIPAM brush in a 0.2 mol% LiBr electrolyte in $x_D = 0$ at 20.0 °C with the superimposed distribution of fits from PT-MCMC sampling.

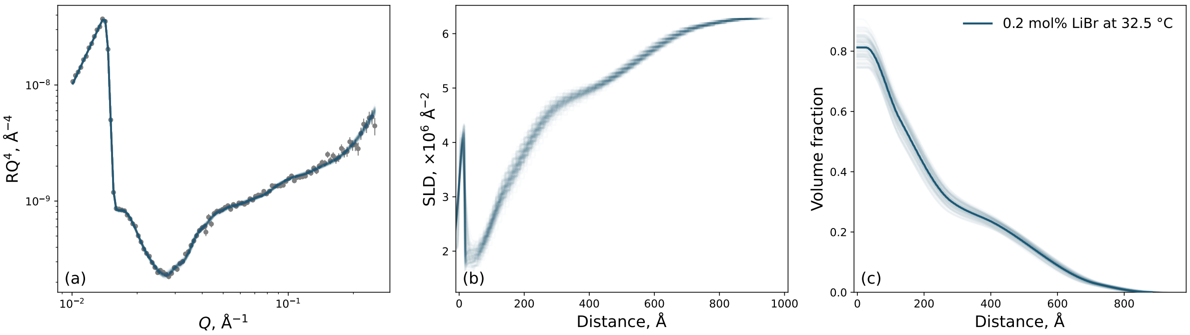

Figure S4.1.2v: (a) Reflectivity, (b) SLD and (c) polymer VF profiles of the 247 Å PNIPAM brush in a 0.2 mol% LiBr electrolyte in $x_D = 0$ at 32.5 °C with the superimposed distribution of fits from PT-MCMC sampling.

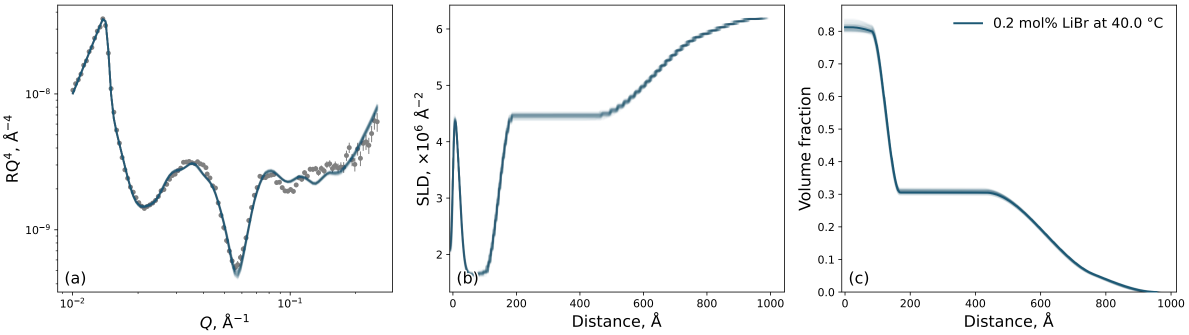

Figure S4.1.2vi: (a) Reflectivity, (b) SLD and (c) polymer VF profiles of the 247 Å PNIPAM brush in a 0.2 mol% LiBr electrolyte in $x_D = 0$ at 40.0 °C with the superimposed distribution of fits from PT-MCMC sampling.

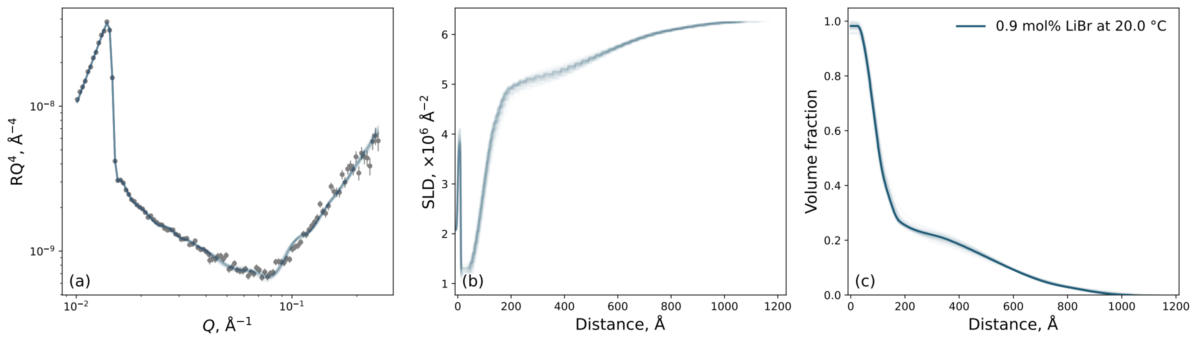

Figure S4.1.2vii: (a) Reflectivity, (b) SLD and (c) polymer VF profiles of the 247 Å PNIPAM brush in a 0.9 mol% LiBr electrolyte in $x_{\mathrm{D}} = 0$ at 20.0 °C with the superimposed distribution of fits from PT-MCMC sampling.

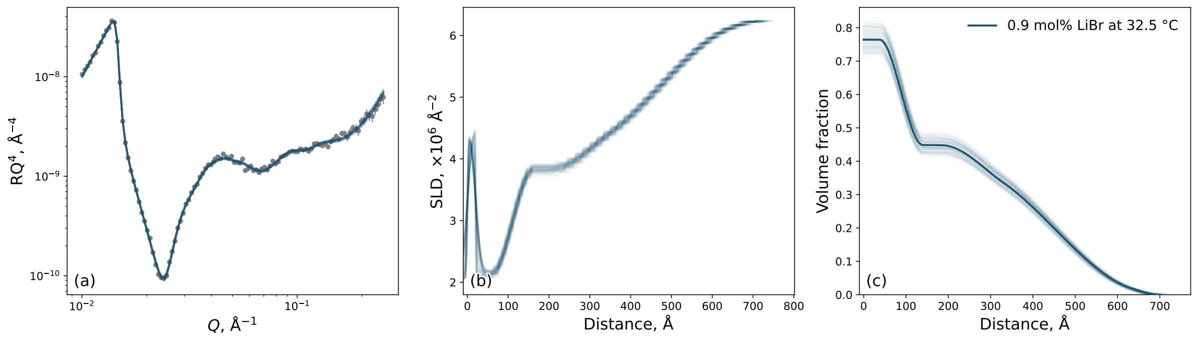

Figure S4.1.2viii: (a) Reflectivity, (b) SLD and (c) polymer VF profiles of the 247 Å PNIPAM brush in a 0.9 mol% LiBr electrolyte in $x_{\mathrm{D}} = 0$ at 32.5 °C with the superimposed distribution of fits from PT-MCMC sampling.

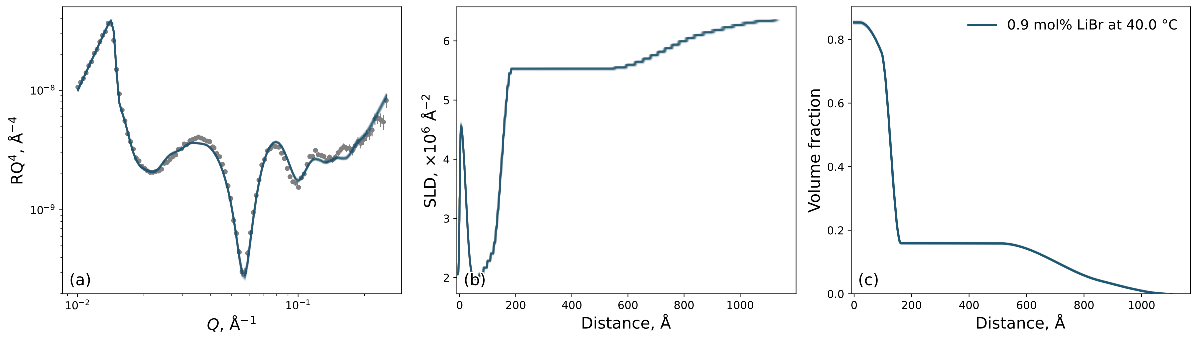

Figure S4.1.2ix: (a) Reflectivity, (b) SLD and (c) polymer VF profiles of the 247 Å PNIPAM brush in a 0.9 mol% LiBr electrolyte in $x_{\mathrm{D}} = 0$ at 40.0 °C with the superimposed distribution of fits from PT-MCMC sampling.

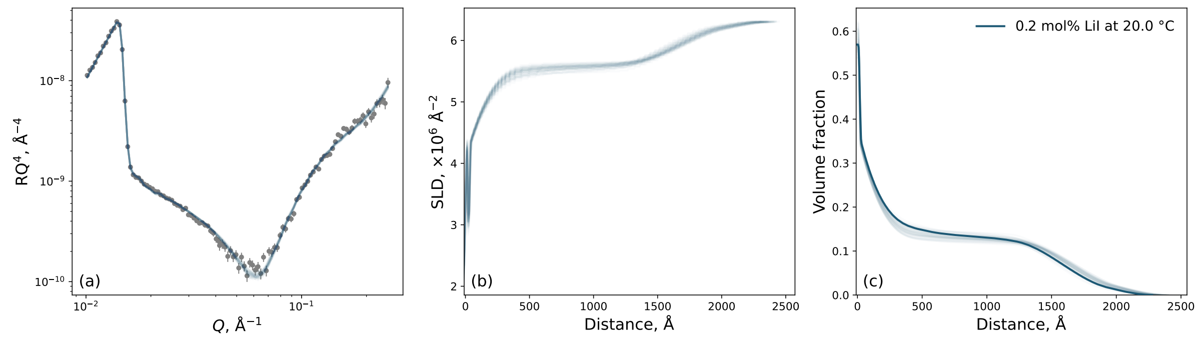

Figure S4.1.2x: (a) Reflectivity, (b) SLD and (c) polymer VF profiles of the 247 Å PNIPAM brush in a 0.2 mol% LiI electrolyte in $x_{\mathrm{D}} = 0$ at 20.0 °C with the superimposed distribution of fits from PT-MCMC sampling.

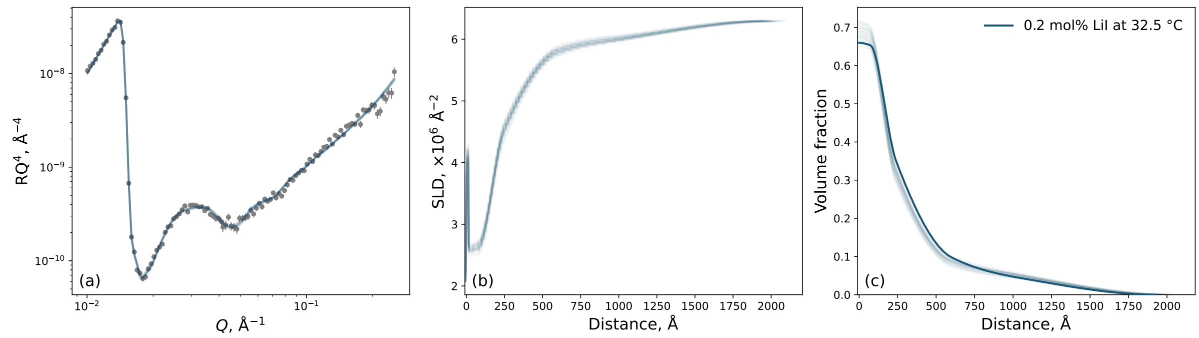

Figure S4.1.2xi: (a) Reflectivity, (b) SLD and (c) polymer VF profiles of the 247 Å PNIPAM brush in a 0.2 mol% LiI electrolyte in $x_{\mathrm{D}} = 0$ at 32.5 °C with the superimposed distribution of fits from PT-MCMC sampling.

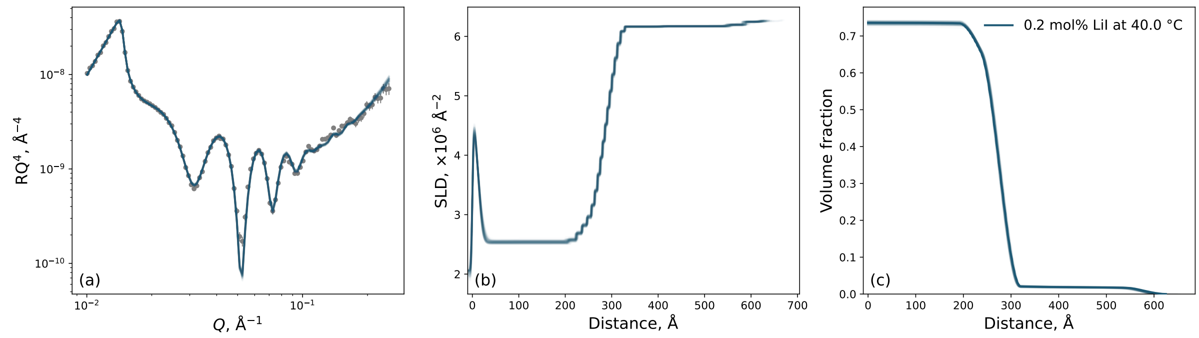

Figure S4.1.2xii: (a) Reflectivity, (b) SLD and (c) polymer VF profiles of the 247 Å PNIPAM brush in a 0.2 mol% LiI electrolyte in $x_{\mathrm{D}} = 0$ at 40.0 °C with the superimposed distribution of fits from PT-MCMC sampling.

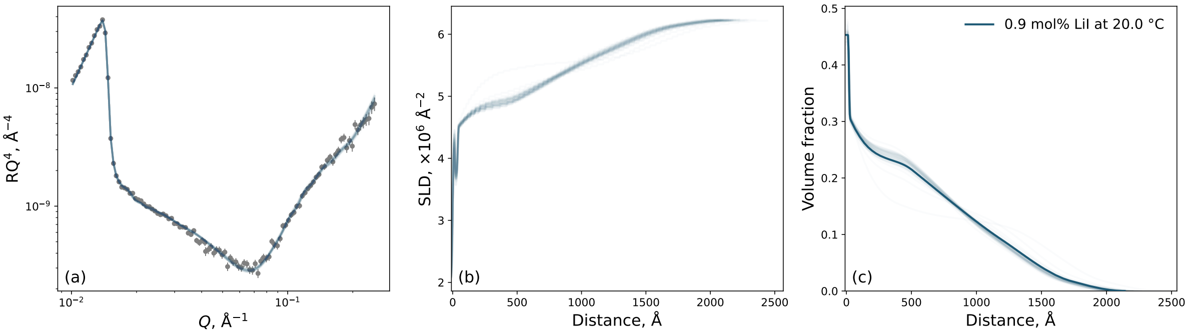

Figure S4.1.2xiii: (a) Reflectivity, (b) SLD and (c) polymer VF profiles of the 247 Å PNIPAM brush in a 0.9 mol% LiI electrolyte in $x_{\mathrm{D}} = 0$ at 20.0 °C with the superimposed distribution of fits from PT-MCMC sampling.

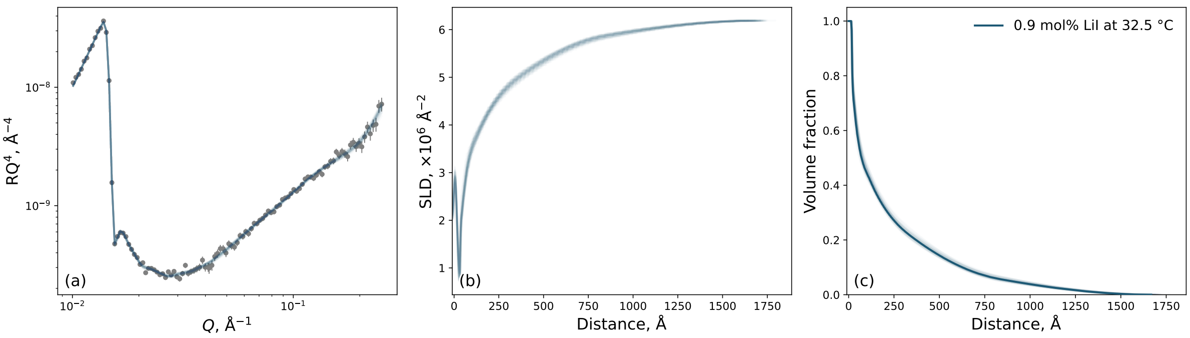

Figure S4.1.2xiv: (a) Reflectivity, (b) SLD and (c) polymer VF profiles of the 247 Å PNIPAM brush in a 0.9 mol% LiI electrolyte in $x_{\mathrm{D}} = 0$ at 32.5 °C with the superimposed distribution of fits from PT-MCMC sampling.

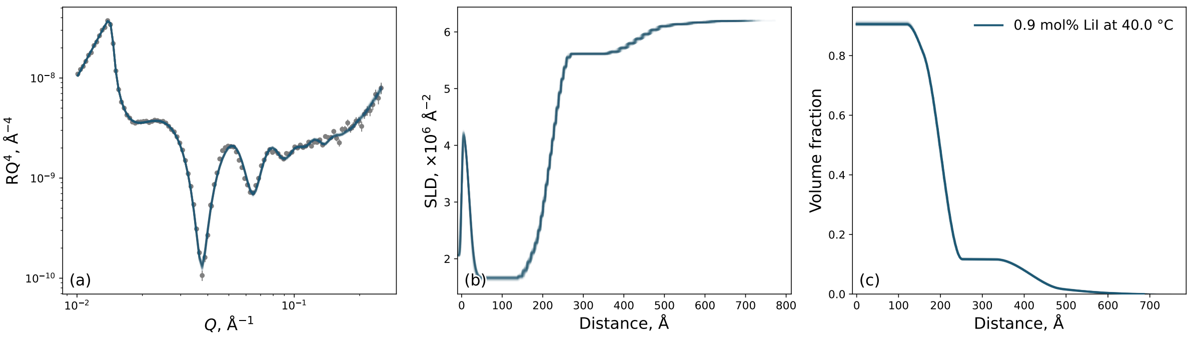

Figure S4.1.2xv: (a) Reflectivity, (b) SLD and (c) polymer VF profiles of the 247 Å PNIPAM brush in a 0.9 mol% LiI electrolyte in $x_{\mathrm{D}} = 0$ at 40.0 °C with the superimposed distribution of fits from PT-MCMC sampling.

4.2 6 mol% DMSO

4.2.1 198 Å PNIPAM brush

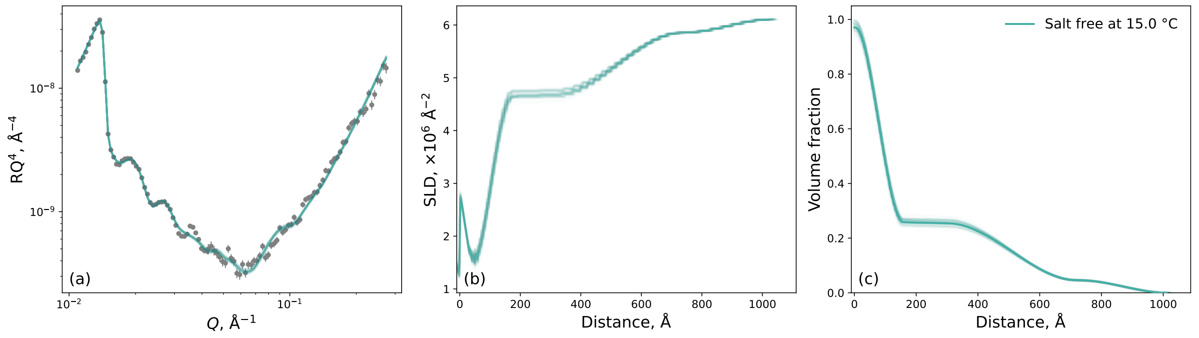

Figure S4.2.1i: (a) Reflectivity, (b) SLD and (c) polymer VF profiles of the 198 Å PNIPAM brush in $x_{\mathrm{D}} = 0.06$ at 15.0 °C with the superimposed distribution of fits from PT-MCMC sampling.

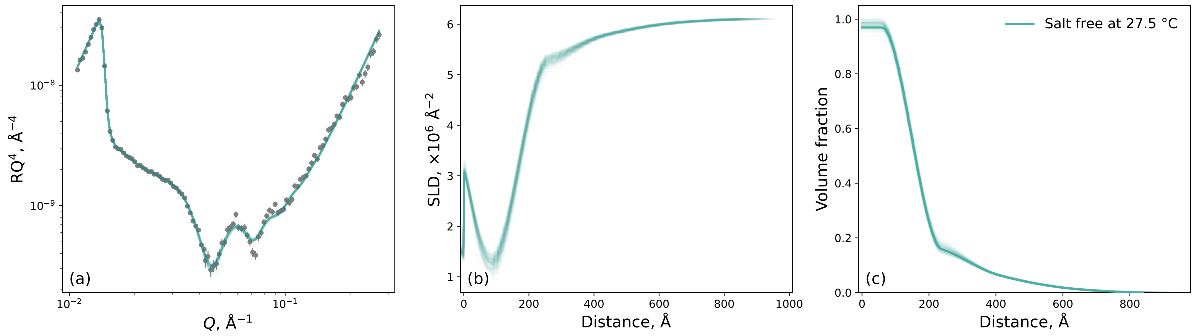

Figure S4.2.1ii: (a) Reflectivity, (b) SLD and (c) polymer VF profiles of the 198 Å PNIPAM brush in $x_{\mathrm{D}} = 0.06$ at 27.5 °C with the superimposed distribution of fits from PT-MCMC sampling.

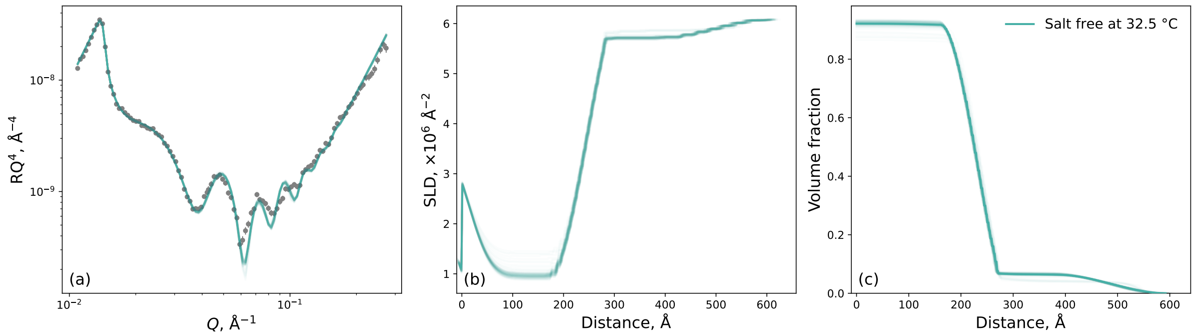

Figure S4.2.1iii: (a) Reflectivity, (b) SLD and (c) polymer VF profiles of the 198 Å PNIPAM brush in $x_{\mathrm{D}} = 0.06$ at 32.5 °C with the superimposed distribution of fits from PT-MCMC sampling.

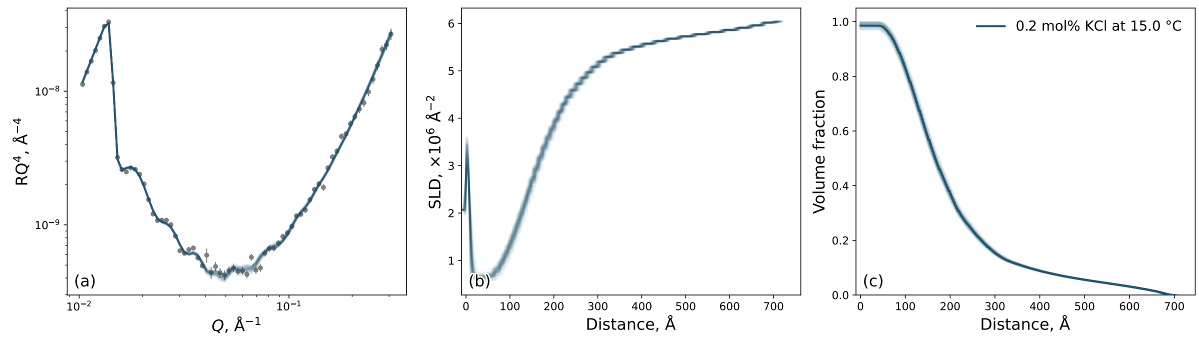

Figure S4.2.1iv: (a) Reflectivity, (b) SLD and (c) polymer VF profiles of the 198 Å PNIPAM brush in a 0.2 mol% KCl electrolyte in $x_{\mathrm{D}} = 0.06$ at 15.0 °C with the superimposed distribution of fits from PT-MCMC sampling.

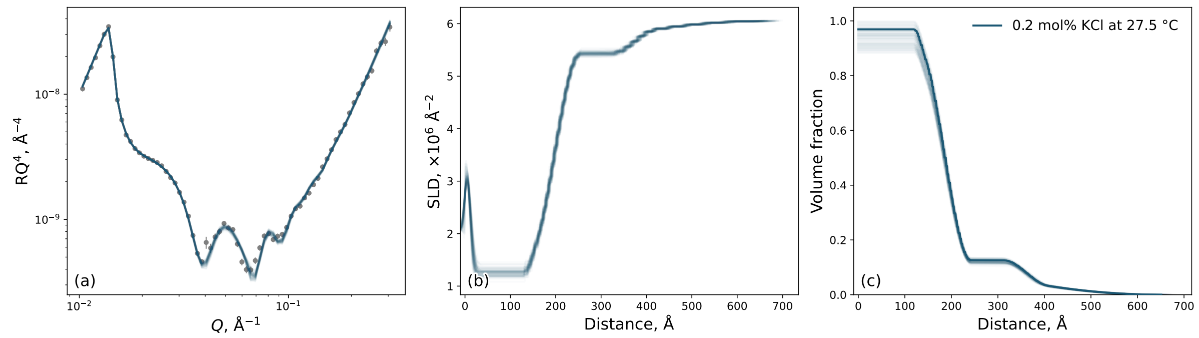

Figure S4.2.1v: (a) Reflectivity, (b) SLD and (c) polymer VF profiles of the 198 Å PNIPAM brush in a 0.2 mol% KCl electrolyte in $x_{\mathrm{D}} = 0.06$ at 27.5 °C with the superimposed distribution of fits from PT-MCMC sampling.

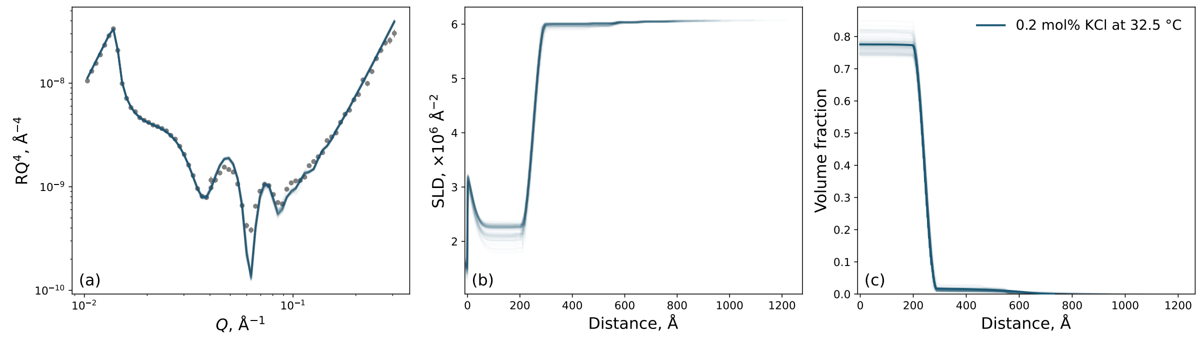

Figure S4.2.1vi: (a) Reflectivity, (b) SLD and (c) polymer VF profiles of the 198 Å PNIPAM brush in a 0.2 mol% KCl electrolyte in $x_{\mathrm{D}} = 0.06$ at 32.5 °C with the superimposed distribution of fits from PT-MCMC sampling.

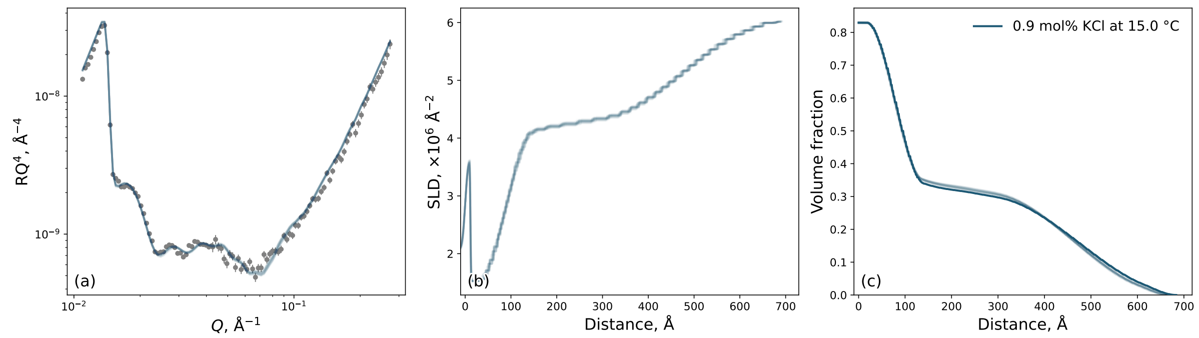

Figure S4.2.1vii: (a) Reflectivity, (b) SLD and (c) polymer VF profiles of the 198 Å PNIPAM brush in a 0.9 mol% KCl electrolyte in $x_{\mathrm{D}} = 0.06$ at 15.0 °C with the superimposed distribution of fits from PT-MCMC sampling.

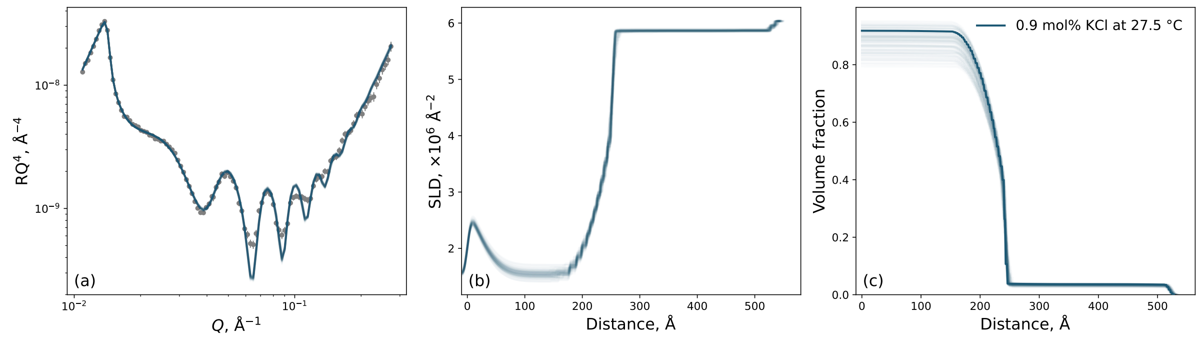

Figure S4.2.1viii: (a) Reflectivity, (b) SLD and (c) polymer VF profiles of the 198 Å PNIPAM brush in a 0.9 mol% KCl electrolyte in $x_{\mathrm{D}} = 0.06$ at 27.5 °C with the superimposed distribution of fits from PT-MCMC sampling.

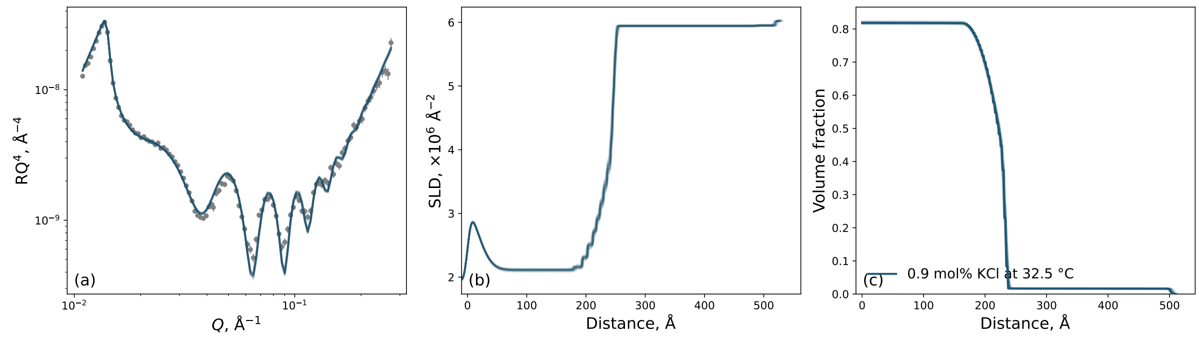

Figure S4.2.1ix: (a) Reflectivity, (b) SLD and (c) polymer VF profiles of the 198 Å PNIPAM brush in a 0.9 mol% KCl electrolyte in $x_{\mathrm{D}} = 0.06$ at 32.5 °C with the superimposed distribution of fits from PT-MCMC sampling.

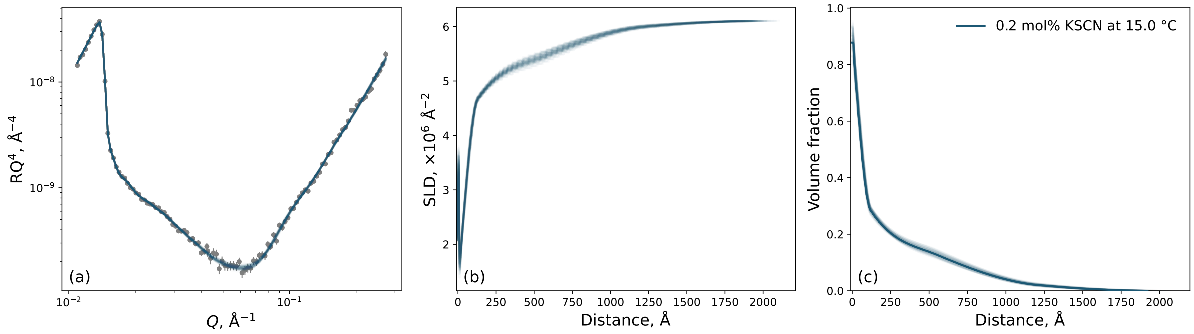

Figure S4.2.1x: (a) Reflectivity, (b) SLD and (c) polymer VF profiles of the 198 Å PNIPAM brush in a 0.2 mol% KSCN electrolyte in $x_{\mathrm{D}} = 0.06$ at 15.0 °C with the superimposed distribution of fits from PT-MCMC sampling.

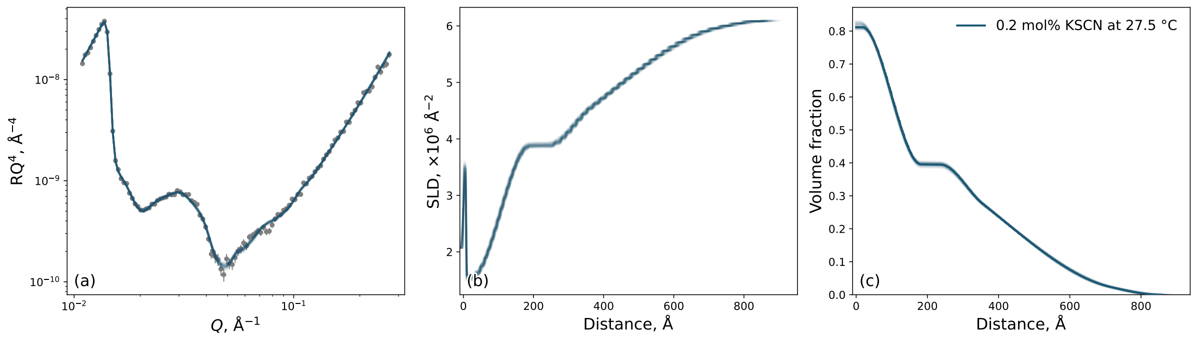

Figure S4.2.1xi: (a) Reflectivity, (b) SLD and (c) polymer VF profiles of the 198 Å PNIPAM brush in a 0.2 mol% KSCN electrolyte in $x_{\mathrm{D}} = 0.06$ at 27.5 °C with the superimposed distribution of fits from PT-MCMC sampling.

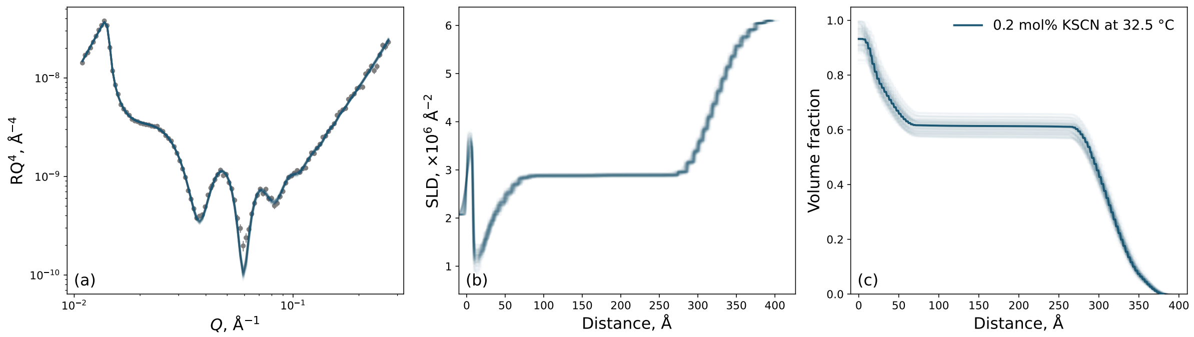

Figure S4.2.1xii: (a) Reflectivity, (b) SLD and (c) polymer VF profiles of the 198 Å PNIPAM brush in a 0.2 mol% KSCN electrolyte in $x_{\mathrm{D}} = 0.06$ at 32.5 °C with the superimposed distribution of fits from PT-MCMC sampling.

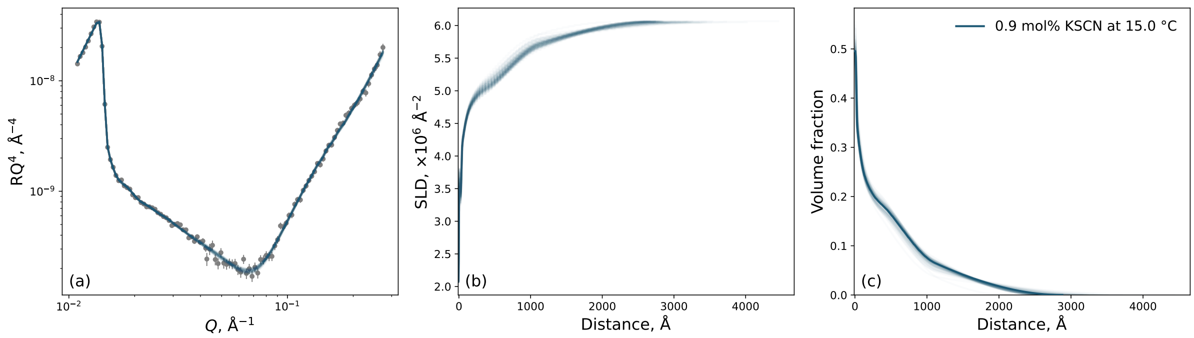

Figure S4.2.1xiii: (a) Reflectivity, (b) SLD and (c) polymer VF profiles of the 198 Å PNIPAM brush in a 0.9 mol% KSCN electrolyte in $x_{\mathrm{D}} = 0.06$ at 15.0 °C with the superimposed distribution of fits from PT-MCMC sampling.

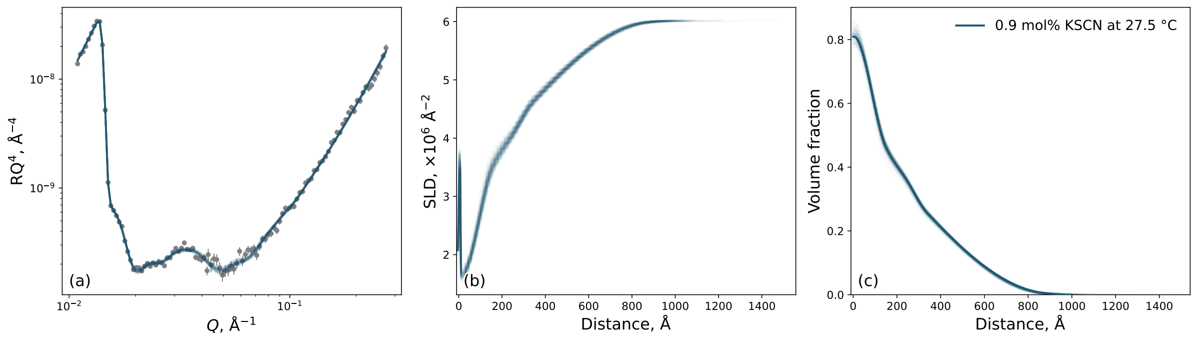

Figure S4.2.1xiv: (a) Reflectivity, (b) SLD and (c) polymer VF profiles of the 198 Å PNIPAM brush in a 0.9 mol% KSCN electrolyte in $x_{\mathrm{D}} = 0.06$ at 27.5 °C with the superimposed distribution of fits from PT-MCMC sampling.

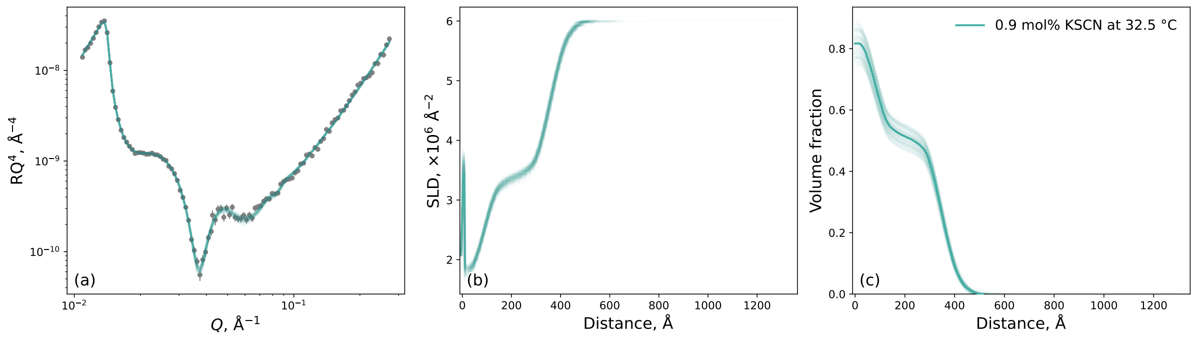

Figure S4.2.1xv: (a) Reflectivity, (b) SLD and (c) polymer VF profiles of the 198 Å PNIPAM brush in a 0.9 mol% KSCN electrolyte in $x_{\mathrm{D}} = 0.06$ at 32.5 °C with the superimposed distribution of fits from PT-MCMC sampling.

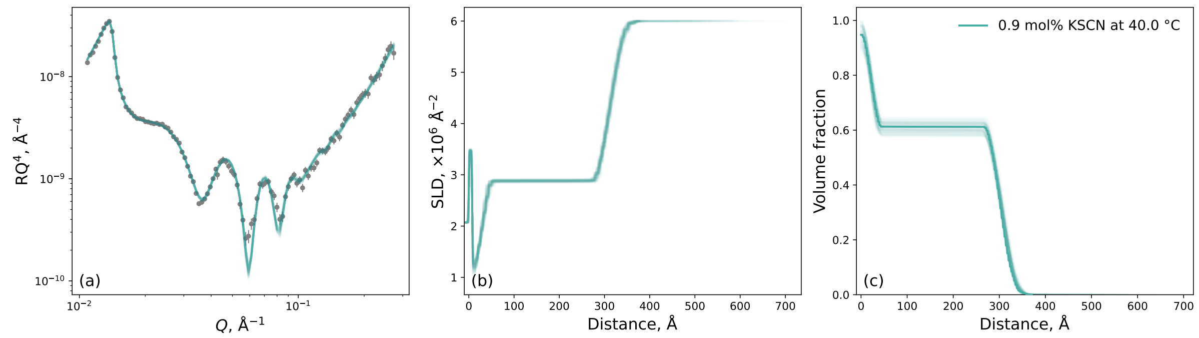

Figure S4.2.1xvi: (a) Reflectivity, (b) SLD and (c) polymer VF profiles of the 198 Å PNIPAM brush in a 0.9 mol% KSCN electrolyte in $x_{\mathrm{D}} = 0.06$ at 40.0 °C with the superimposed distribution of fits from PT-MCMC sampling.

4.2.2 247 Å PNIPAM brush

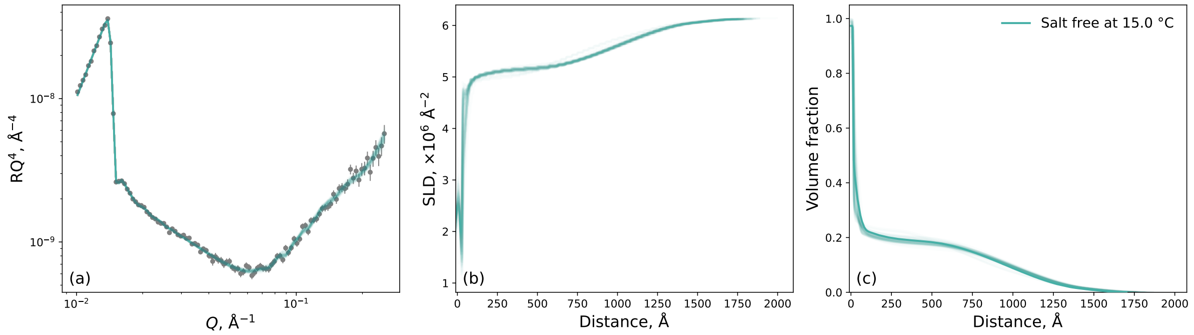

Figure S4.2.2i: (a) Reflectivity, (b) SLD and (c) polymer VF profiles of the 247 Å PNIPAM brush in $x_\mathrm{D} = 0.06$ at 15.0 °C with the superimposed distribution of fits from PT-MCMC sampling.

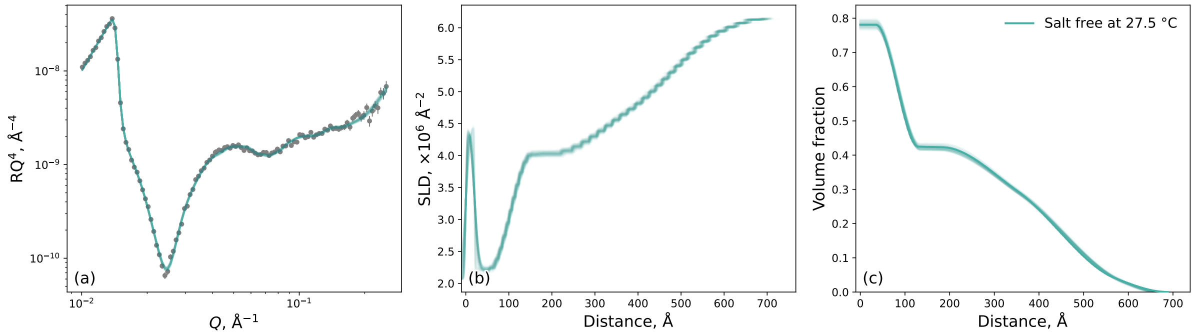

Figure S4.2.2ii: (a) Reflectivity, (b) SLD and (c) polymer VF profiles of the 247 Å PNIPAM brush in $x_\mathrm{D} = 0.06$ at 27.5 °C with the superimposed distribution of fits from PT-MCMC sampling.

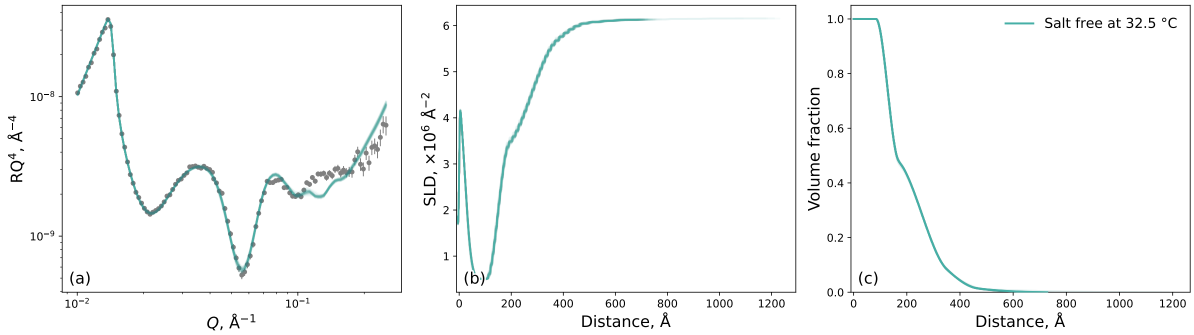

Figure S4.2.2iii: (a) Reflectivity, (b) SLD and (c) polymer VF profiles of the 247 Å PNIPAM brush in $x_\mathrm{D} = 0.06$ at 32.5 °C with the superimposed distribution of fits from PT-MCMC sampling.

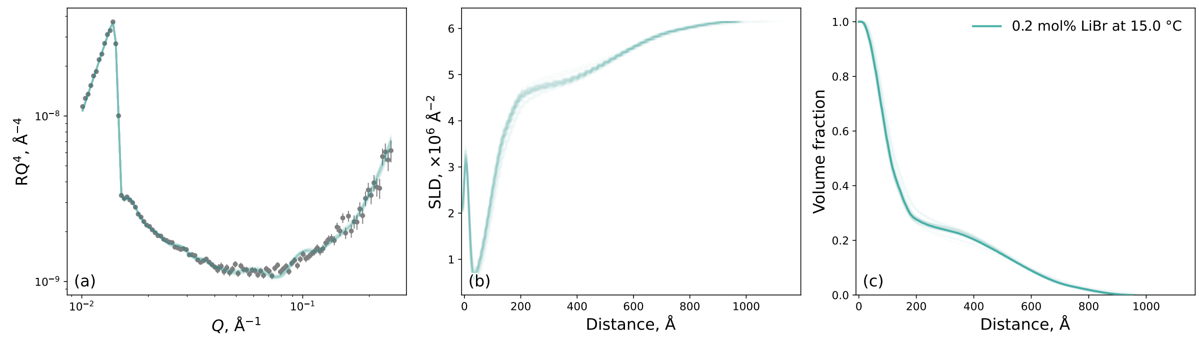

Figure S4.2.2iv: (a) Reflectivity, (b) SLD and (c) polymer VF profiles of the 247 Å PNIPAM brush in a 0.2 mol% LiBr electrolyte in $x_{\mathrm{D}} = 0.06$ at 15.0 °C with the superimposed distribution of fits from PT-MCMC sampling.

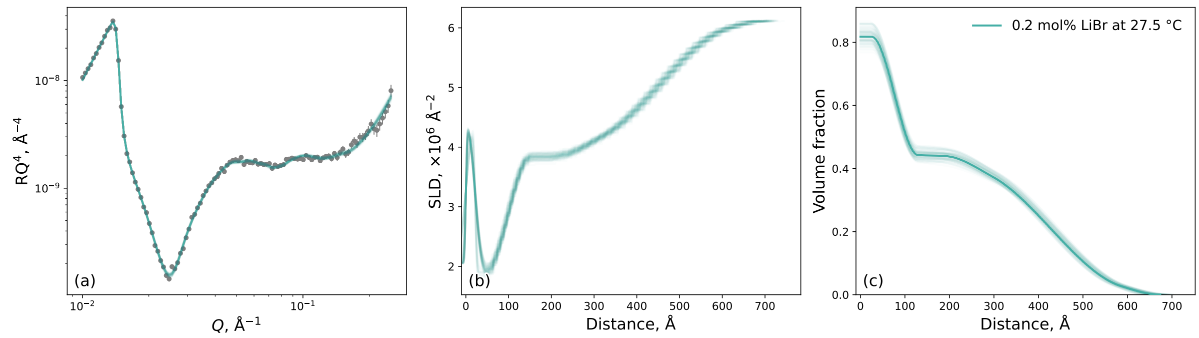

Figure S4.2.2v: (a) Reflectivity, (b) SLD and (c) polymer VF profiles of the 247 Å PNIPAM brush in a 0.2 mol% LiBr electrolyte in $x_{\mathrm{D}} = 0.06$ at 27.5 °C with the superimposed distribution of fits from PT-MCMC sampling.

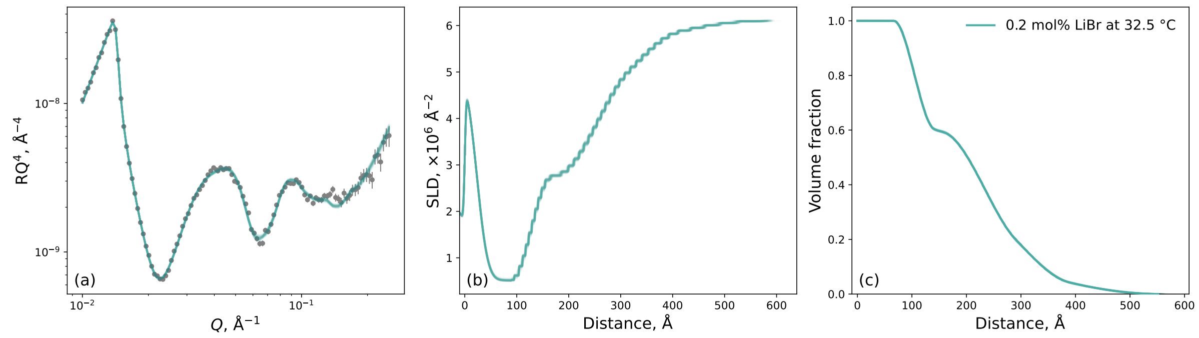

Figure S4.2.2vi: (a) Reflectivity, (b) SLD and (c) polymer VF profiles of the 247 Å PNIPAM brush in a 0.2 mol% LiBr electrolyte in $x_{\mathrm{D}} = 0.06$ at 32.5 °C with the superimposed distribution of fits from PT-MCMC sampling.

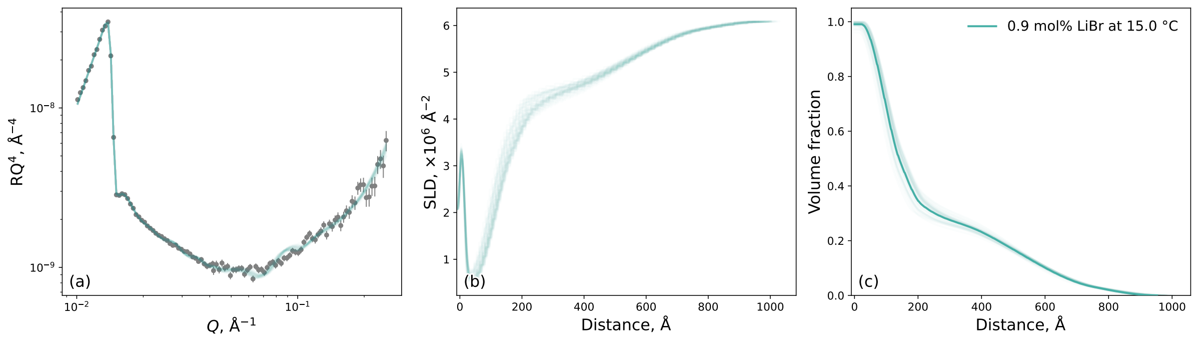

Figure S4.2.2vii: (a) Reflectivity, (b) SLD and (c) polymer VF profiles of the 247 Å PNIPAM brush in a 0.9 mol% LiBr electrolyte in $x_D = 0.06$ at 15.0 °C with the superimposed distribution of fits from PT-MCMC sampling.

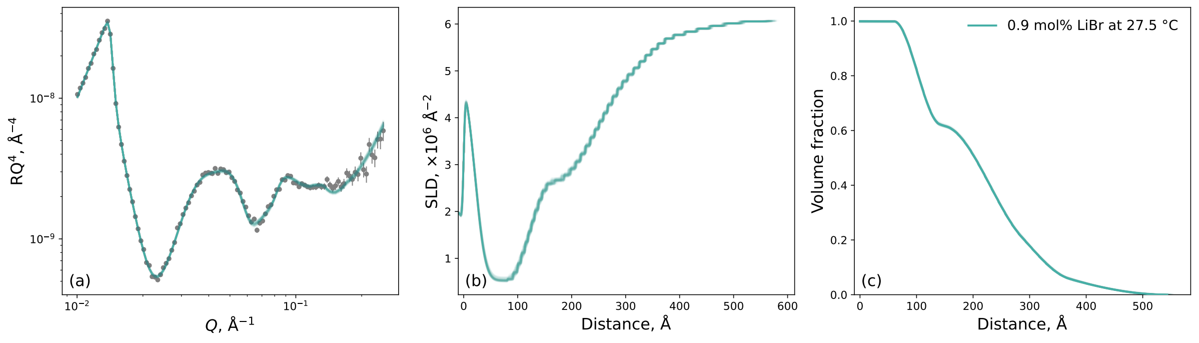

Figure S4.2.2viii: (a) Reflectivity, (b) SLD and (c) polymer VF profiles of the 247 Å PNIPAM brush in a 0.9 mol% LiBr electrolyte in $x_D = 0.06$ at 27.5 °C with the superimposed distribution of fits from PT-MCMC sampling.

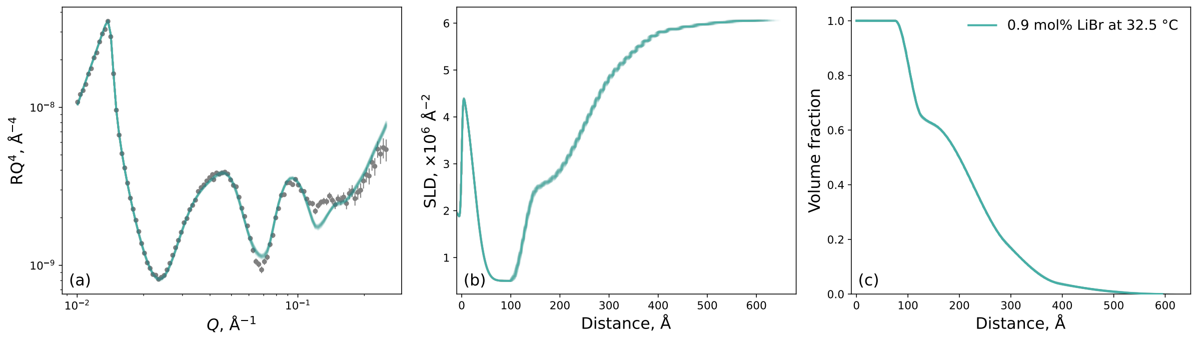

Figure S4.2.2ix: (a) Reflectivity, (b) SLD and (c) polymer VF profiles of the 247 Å PNIPAM brush in a 0.9 mol% LiBr electrolyte in $x_D = 0.06$ at 32.5 °C with the superimposed distribution of fits from PT-MCMC sampling.

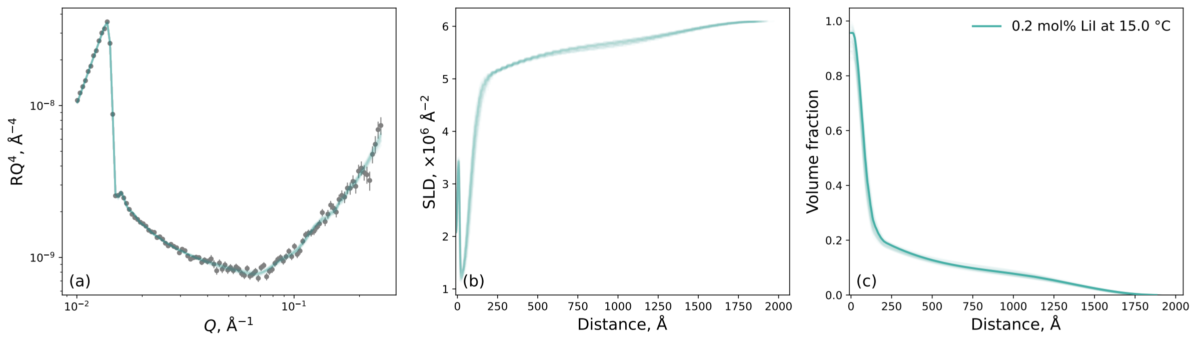

Figure S4.2.2x: (a) Reflectivity, (b) SLD and (c) polymer VF profiles of the 247 Å PNIPAM brush in a 0.2 mol% LiI electrolyte in $x_{\mathrm{D}} = 0.06$ at 15.0 °C with the superimposed distribution of fits from PT-MCMC sampling.

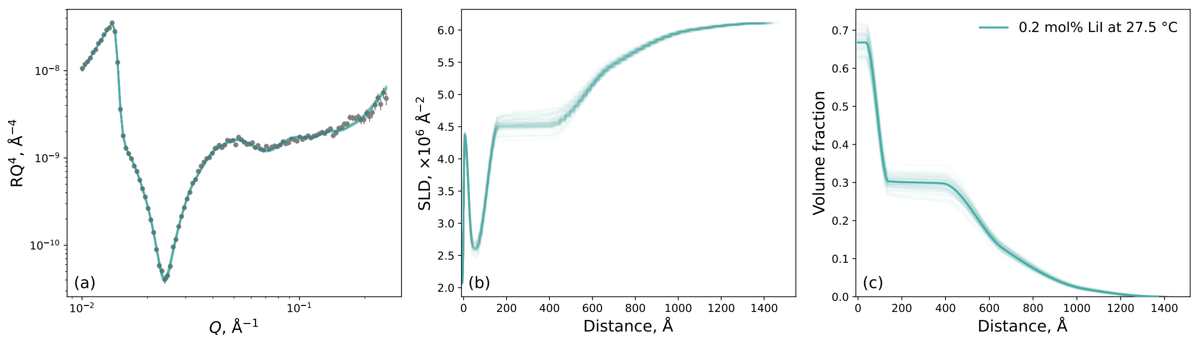

Figure S4.2.2xi: (a) Reflectivity, (b) SLD and (c) polymer VF profiles of the 247 Å PNIPAM brush in a 0.2 mol% LiI electrolyte in $x_{\mathrm{D}} = 0.06$ at 27.5 °C with the superimposed distribution of fits from PT-MCMC sampling.

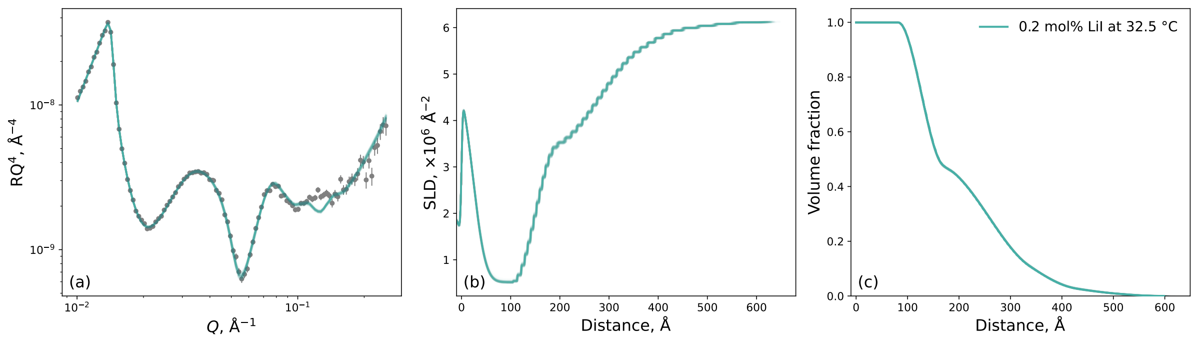

Figure S4.2.2xii: (a) Reflectivity, (b) SLD and (c) polymer VF profiles of the 247 Å PNIPAM brush in a 0.2 mol% LiI electrolyte in $x_{\mathrm{D}} = 0.06$ at 32.5 °C with the superimposed distribution of fits from PT-MCMC sampling.

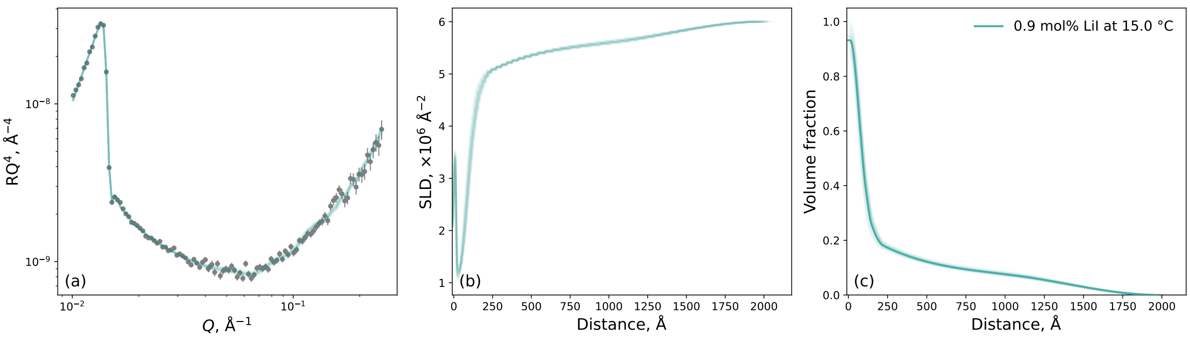

Figure S4.2.2xiii: (a) Reflectivity, (b) SLD and (c) polymer VF profiles of the 247 Å PNIPAM brush in a 0.9 mol% LiI electrolyte in $x_D = 0.06$ at 15.0 °C with the superimposed distribution of fits from PT-MCMC sampling.

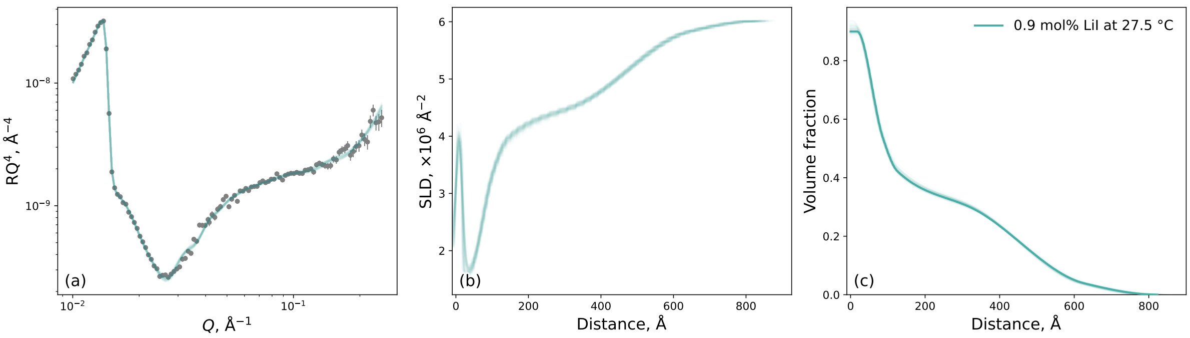

Figure S4.2.2xiv: (a) Reflectivity, (b) SLD and (c) polymer VF profiles of the 247 Å PNIPAM brush in a 0.9 mol% LiI electrolyte in $x_D = 0.06$ at 27.5 °C with the superimposed distribution of fits from PT-MCMC sampling.

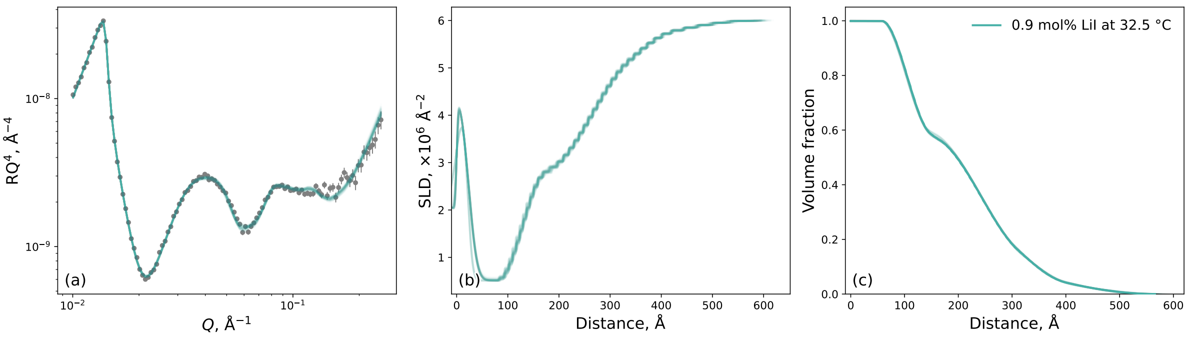

Figure S4.2.2xv: (a) Reflectivity, (b) SLD and (c) polymer VF profiles of the 247 Å PNIPAM brush in a 0.9 mol% LiI electrolyte in $x_D = 0.06$ at 32.5 °C with the superimposed distribution of fits from PT-MCMC sampling.

4.3 70 mol% DMSO

4.3.1 244 Å PNIPAM brush

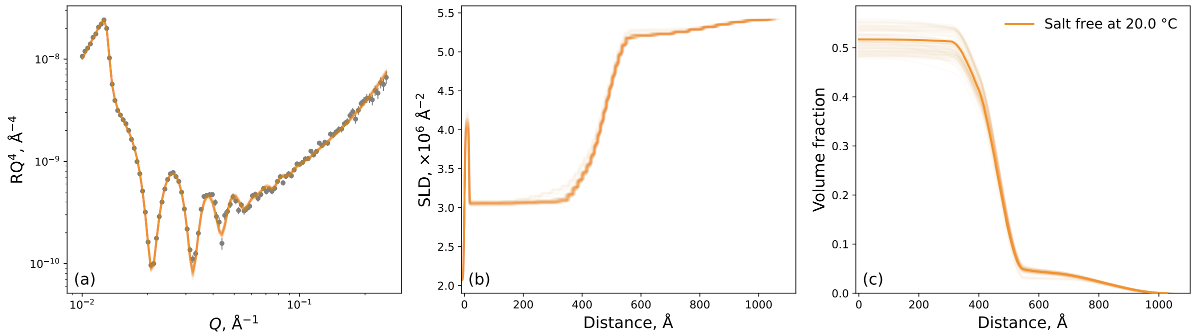

Figure S4.3.1i: (a) Reflectivity, (b) SLD and (c) polymer VF profiles of the 244 Å PNIPAM brush in $x_\mathrm{D} = 0.70$ at 20.0 °C with the superimposed distribution of fits from PT-MCMC sampling.

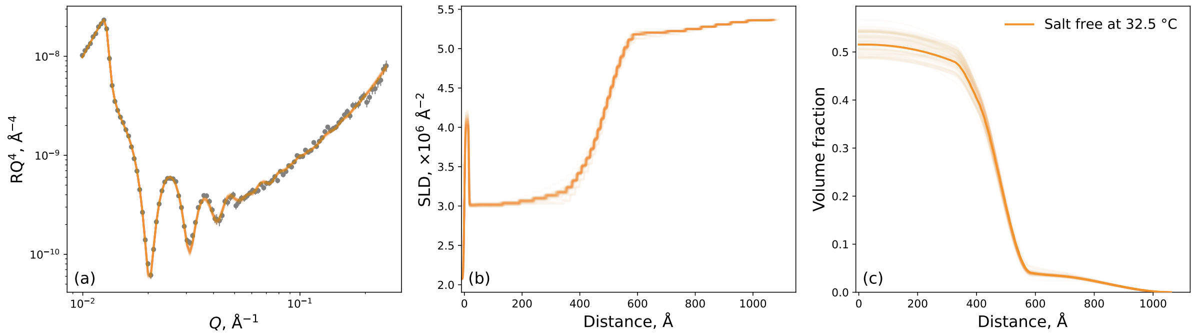

Figure S4.3.1ii: (a) Reflectivity, (b) SLD and (c) polymer VF profiles of the 244 Å PNIPAM brush in $x_\mathrm{D} = 0.70$ at 32.5 °C with the superimposed distribution of fits from PT-MCMC sampling.

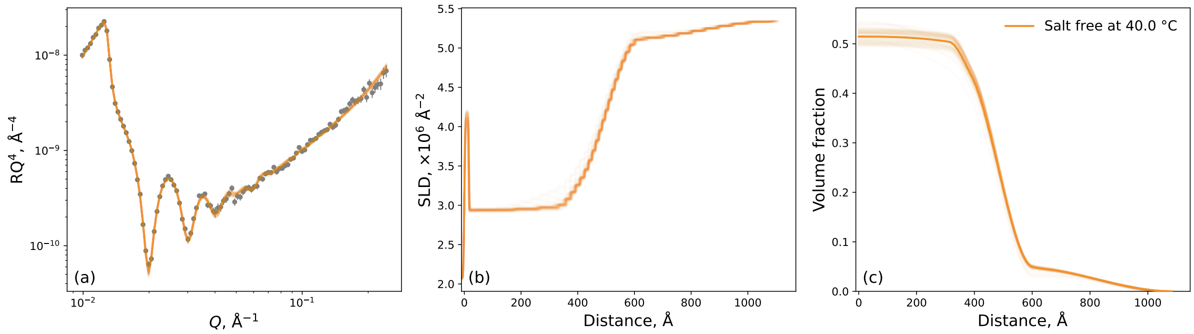

Figure S4.3.1iii: (a) Reflectivity, (b) SLD and (c) polymer VF profiles of the 244 Å PNIPAM brush in $x_\mathrm{D} = 0.70$ at 40.0 °C with the superimposed distribution of fits from PT-MCMC sampling.

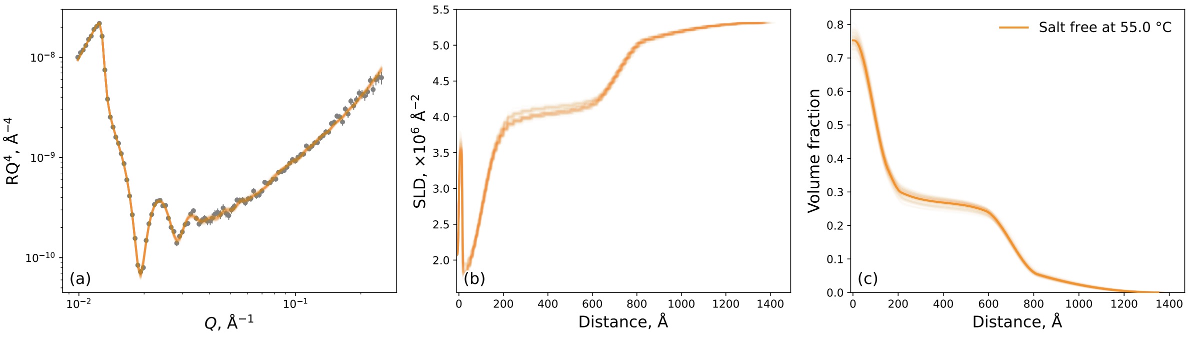

Figure S4.3.1iv: (a) Reflectivity, (b) SLD and (c) polymer VF profiles of the 244 Å PNIPAM brush in $x_\mathrm{D} = 0.70$ at 55.0 °C with the superimposed distribution of fits from PT-MCMC sampling.

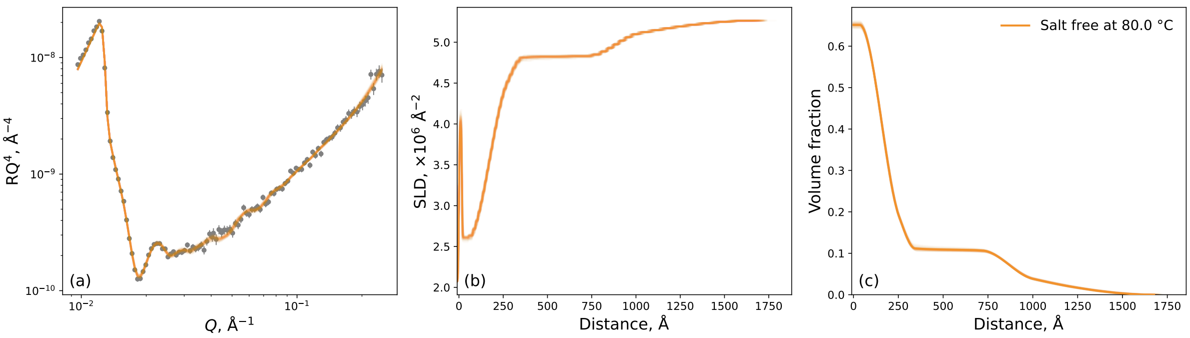

Figure S4.3.1v: (a) Reflectivity, (b) SLD and (c) polymer VF profiles of the 244 Å PNIPAM brush in $x_\mathrm{D} = 0.70$ at 80.0 °C with the superimposed distribution of fits from PT-MCMC sampling.

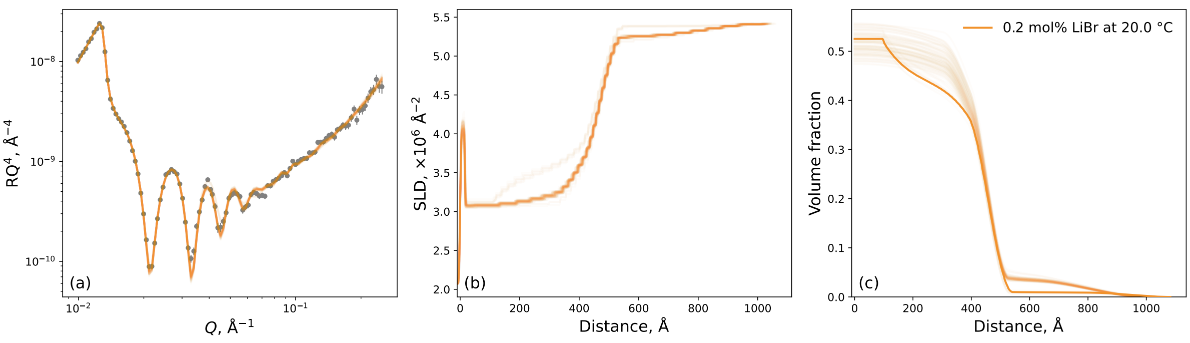

Figure S4.3.1vi: (a) Reflectivity, (b) SLD and (c) polymer VF profiles of the 244 Å PNIPAM brush in a 0.2 mol% LiBr electrolyte in $x_\mathrm{D} = 0.70$ at 20.0 °C with the superimposed distribution of fits from PT-MCMC sampling.

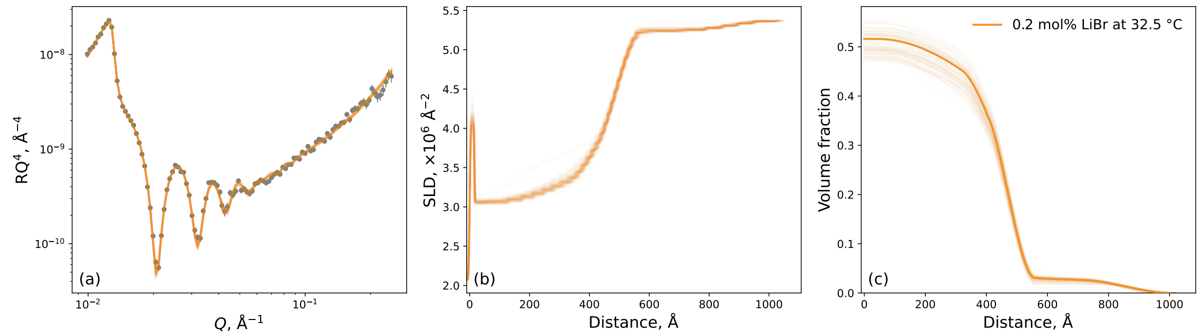

Figure S4.3.1vii: (a) Reflectivity, (b) SLD and (c) polymer VF profiles of the 244 Å PNIPAM brush in a 0.2 mol% LiBr electrolyte in $x_{\mathrm{D}} = 0.70$ at 32.5 °C with the superimposed distribution of fits from PT-MCMC sampling.

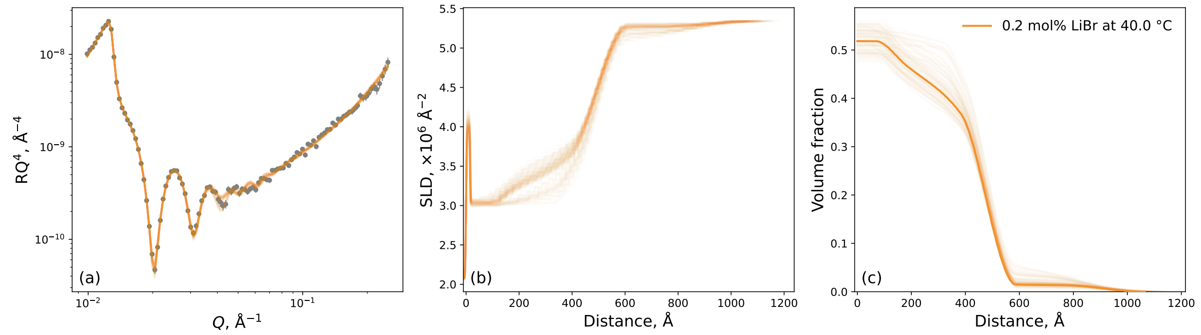

Figure S4.3.1viii: (a) Reflectivity, (b) SLD and (c) polymer VF profiles of the 244 Å PNIPAM brush in a 0.2 mol% LiBr electrolyte in $x_{\mathrm{D}} = 0.70$ at 40.0 °C with the superimposed distribution of fits from PT-MCMC sampling.

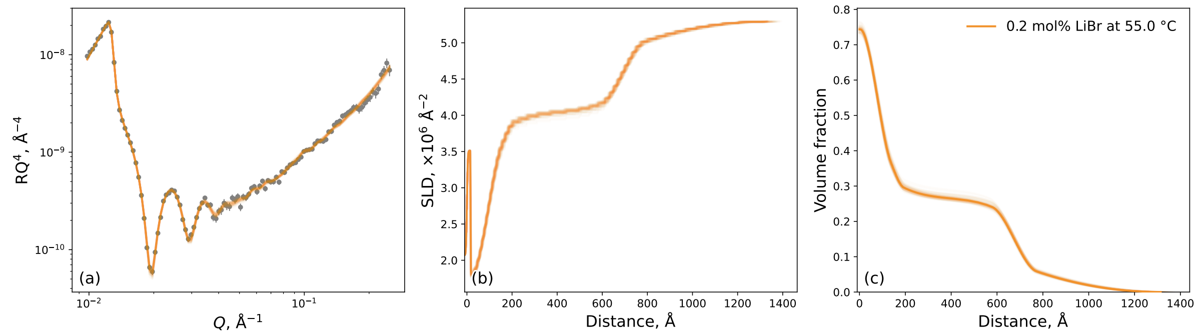

Figure S4.3.1ix: (a) Reflectivity, (b) SLD and (c) polymer VF profiles of the 244 Å PNIPAM brush in a 0.2 mol% LiBr electrolyte in $x_{\mathrm{D}} = 0.70$ at 55.0 °C with the superimposed distribution of fits from PT-MCMC sampling.

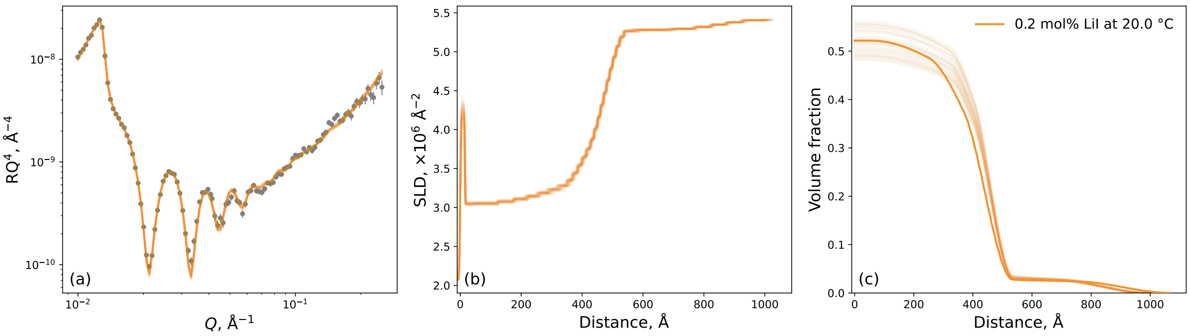

Figure S4.3.1x: (a) Reflectivity, (b) SLD and (c) polymer VF profiles of the 244 Å PNIPAM brush in a 0.2 mol% LiI electrolyte in $x_{\mathrm{D}} = 0.70$ at 20.0 °C with the superimposed distribution of fits from PT-MCMC sampling.

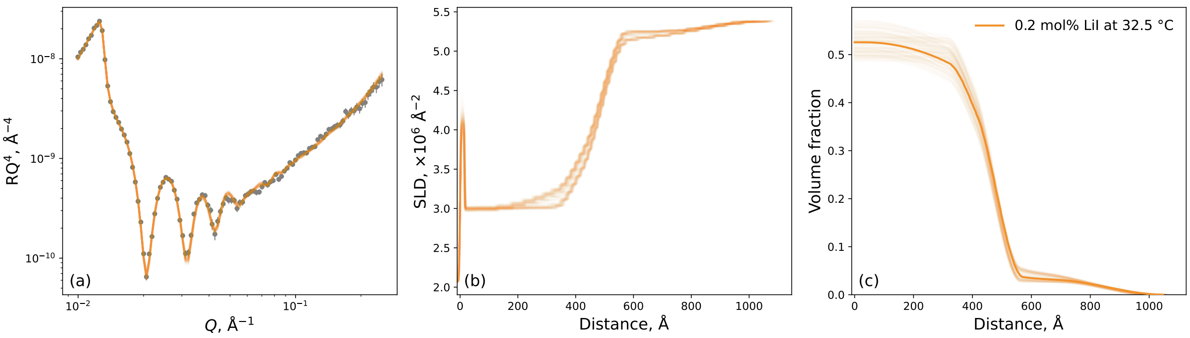

Figure S4.3.1xi: (a) Reflectivity, (b) SLD and (c) polymer VF profiles of the 244 Å PNIPAM brush in a 0.2 mol% LiI electrolyte in $x_{\mathrm{D}} = 0.70$ at 32.5 °C with the superimposed distribution of fits from PT-MCMC sampling.

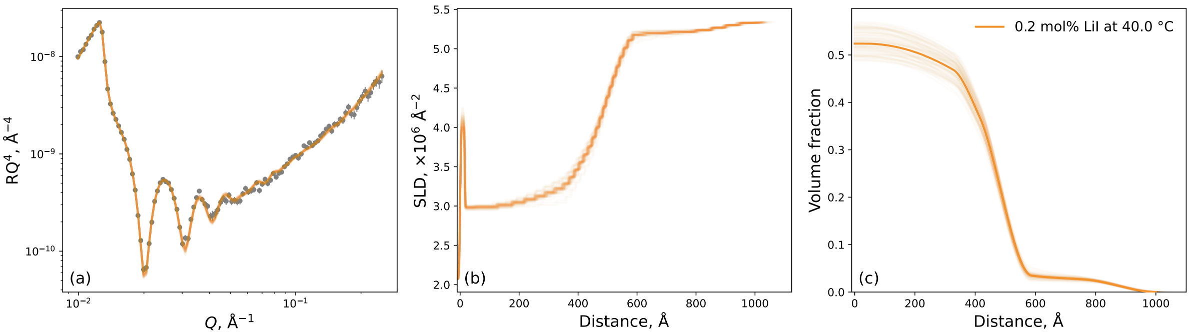

Figure S4.3.1xii: (a) Reflectivity, (b) SLD and (c) polymer VF profiles of the 244 Å PNIPAM brush in a 0.2 mol% LiI electrolyte in $x_{\mathrm{D}} = 0.70$ at 40.0 °C with the superimposed distribution of fits from PT-MCMC sampling.

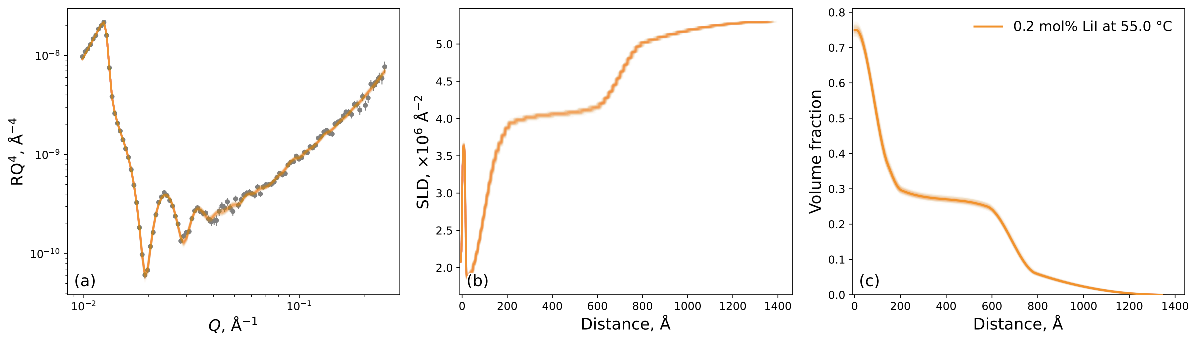

Figure S4.3.1xiii: (a) Reflectivity, (b) SLD and (c) polymer VF profiles of the 244 Å PNIPAM brush in a 0.2 mol% LiI electrolyte in $x_{\mathrm{D}} = 0.70$ at 55.0 °C with the superimposed distribution of fits from PT-MCMC sampling.

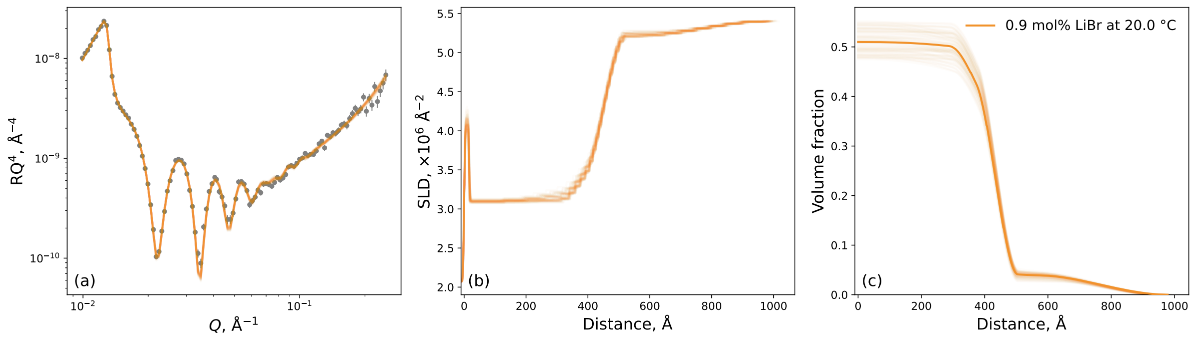

Figure S4.3.1xiv: (a) Reflectivity, (b) SLD and (c) polymer VF profiles of the 244 Å PNIPAM brush in a 0.9 mol% LiBr electrolyte in $x_{\mathrm{D}} = 0.70$ at 20.0 °C with the superimposed distribution of fits from PT-MCMC sampling.

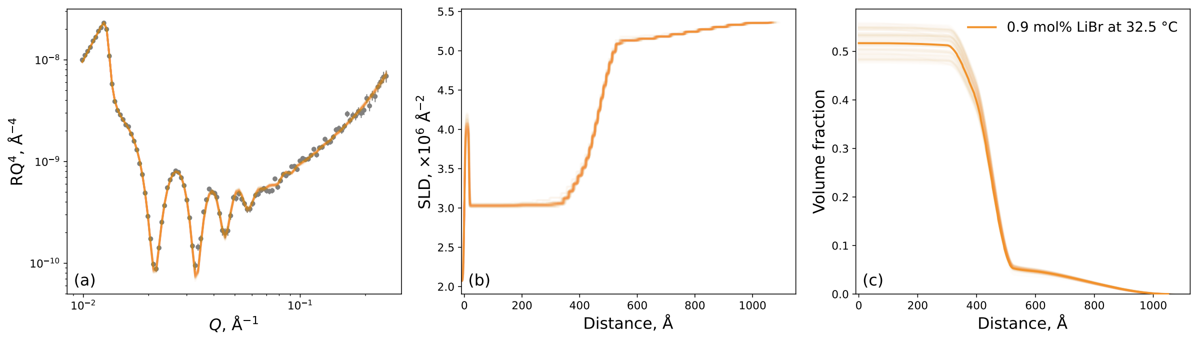

Figure S4.3.1xv: (a) Reflectivity, (b) SLD and (c) polymer VF profiles of the 244 Å PNIPAM brush in a 0.9 mol% LiBr electrolyte in $x_{\mathrm{D}} = 0.70$ at 32.5 °C with the superimposed distribution of fits from PT-MCMC sampling.

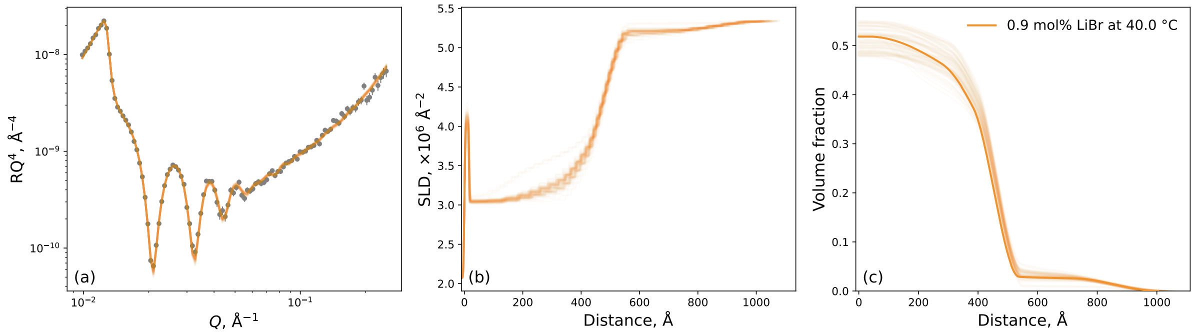

Figure S4.3.1xvi: (a) Reflectivity, (b) SLD and (c) polymer VF profiles of the 244 Å PNIPAM brush in a 0.9 mol% LiBr electrolyte in $x_D = 0.70$ at 40.0 °C with the superimposed distribution of fits from PT-MCMC sampling.

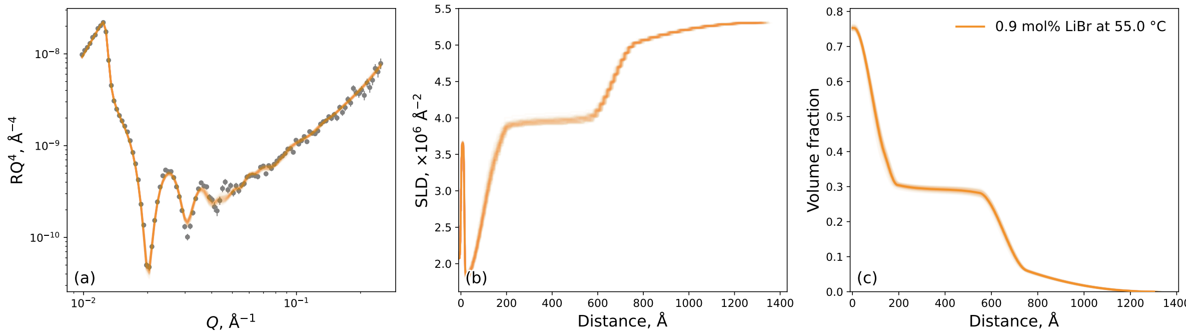

Figure S4.3.1xvii: (a) Reflectivity, (b) SLD and (c) polymer VF profiles of the 244 Å PNIPAM brush in a 0.9 mol% LiBr electrolyte in $x_D = 0.70$ at 55.0 °C with the superimposed distribution of fits from PT-MCMC sampling.

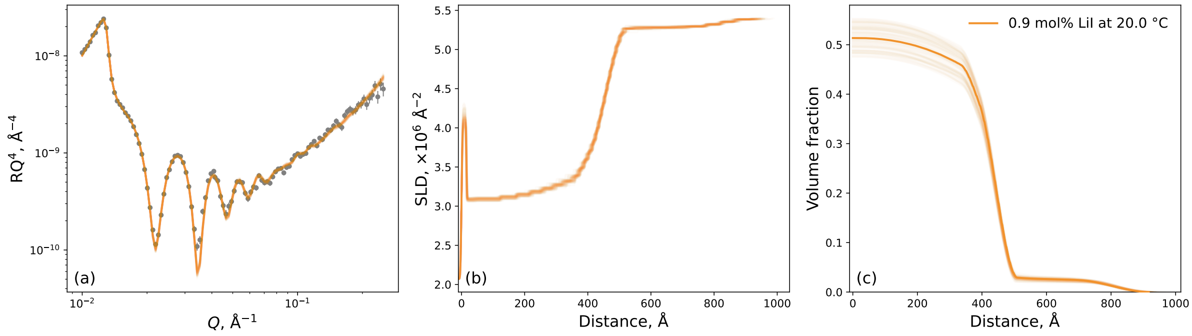

Figure S4.3.1xviii: (a) Reflectivity, (b) SLD and (c) polymer VF profiles of the 244 Å PNIPAM brush in a 0.9 mol% LiI electrolyte in $x_D = 0.70$ at 20.0 °C with the superimposed distribution of fits from PT-MCMC sampling.

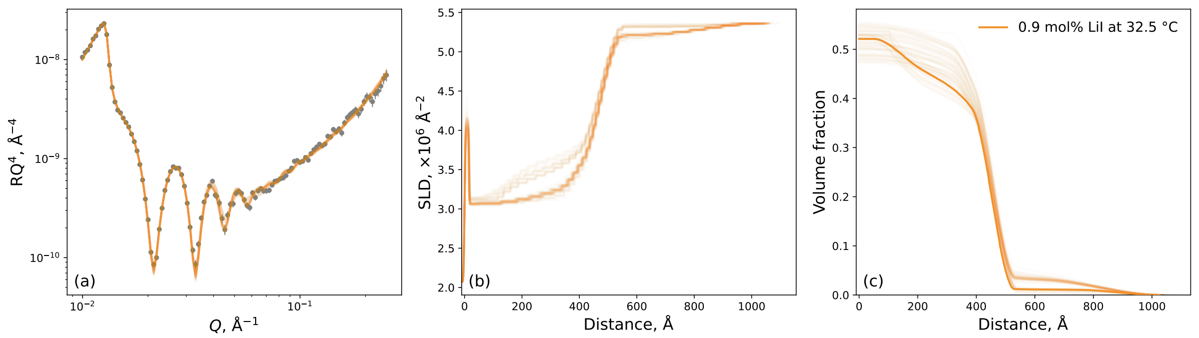

Figure S4.3.1xix: (a) Reflectivity, (b) SLD and (c) polymer VF profiles of the 244 Å PNIPAM brush in a 0.9 mol% LiI electrolyte in $x_{\mathrm{D}} = 0.70$ at 32.5 °C with the superimposed distribution of fits from PT-MCMC sampling.

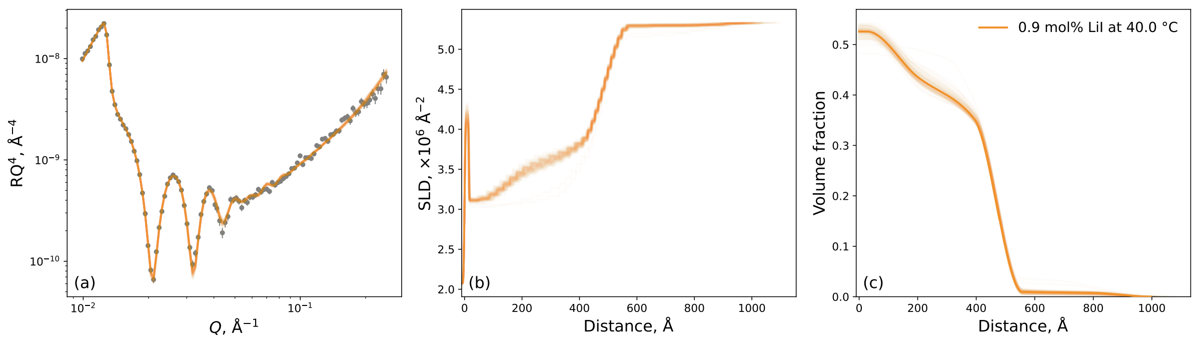

Figure S4.3.1xx: (a) Reflectivity, (b) SLD and (c) polymer VF profiles of the 244 Å PNIPAM brush in a 0.9 mol% LiI electrolyte in $x_{\mathrm{D}} = 0.70$ at 40.0 °C with the superimposed distribution of fits from PT-MCMC sampling.

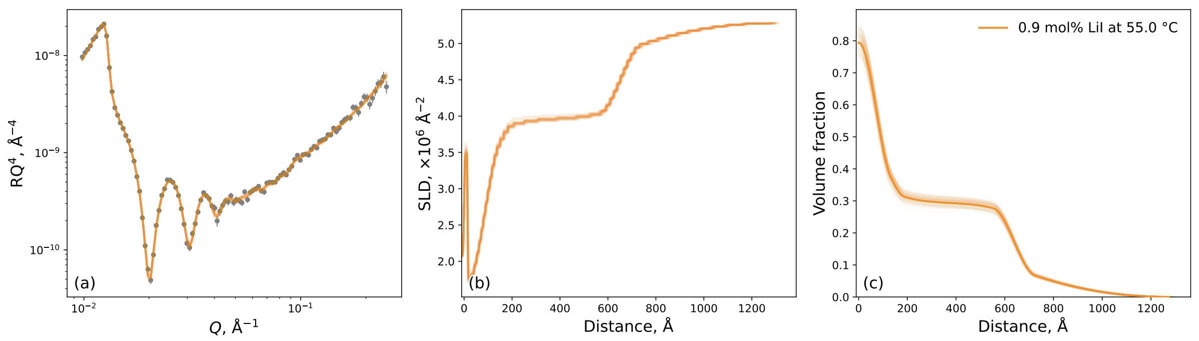

Figure S4.3.1xxi: (a) Reflectivity, (b) SLD and (c) polymer VF profiles of the 244 Å PNIPAM brush in a 0.9 mol% LiI electrolyte in $x_{\mathrm{D}} = 0.70$ at 55.0 °C with the superimposed distribution of fits from PT-MCMC sampling.

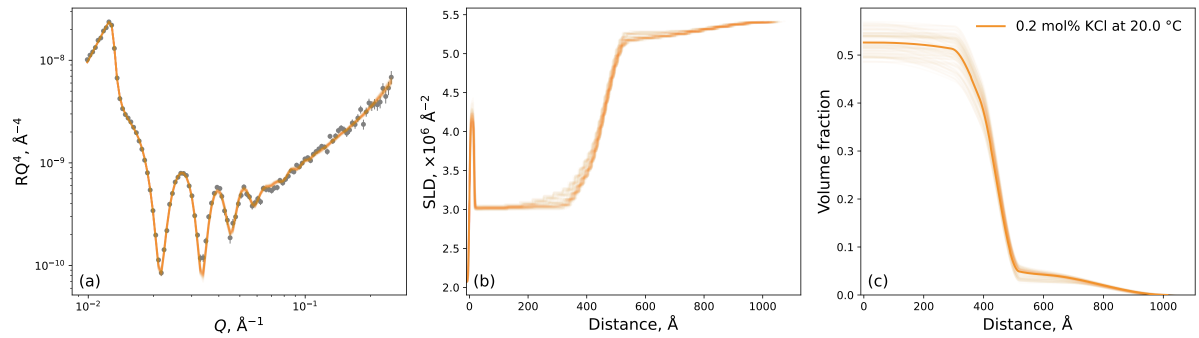

Figure S4.3.1xxii: (a) Reflectivity, (b) SLD and (c) polymer VF profiles of the 244 Å PNIPAM brush in a 0.2 mol% KCl electrolyte in $x_{\mathrm{D}} = 0.70$ at 20.0 °C with the superimposed distribution of fits from PT-MCMC sampling.

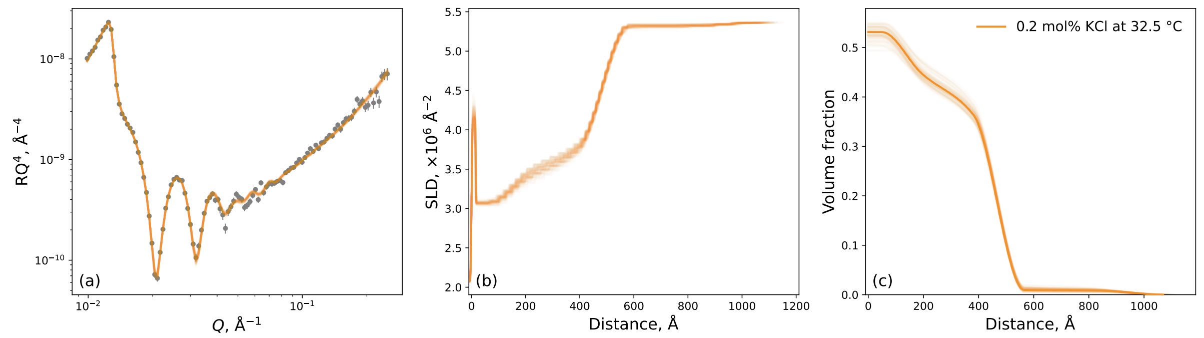

Figure S4.3.1xxiii: (a) Reflectivity, (b) SLD and (c) polymer VF profiles of the 244 Å PNIPAM brush in a 0.2 mol% KCl electrolyte in $x_{\mathrm{D}} = 0.70$ at 32.5 °C with the superimposed distribution of fits from PT-MCMC sampling.

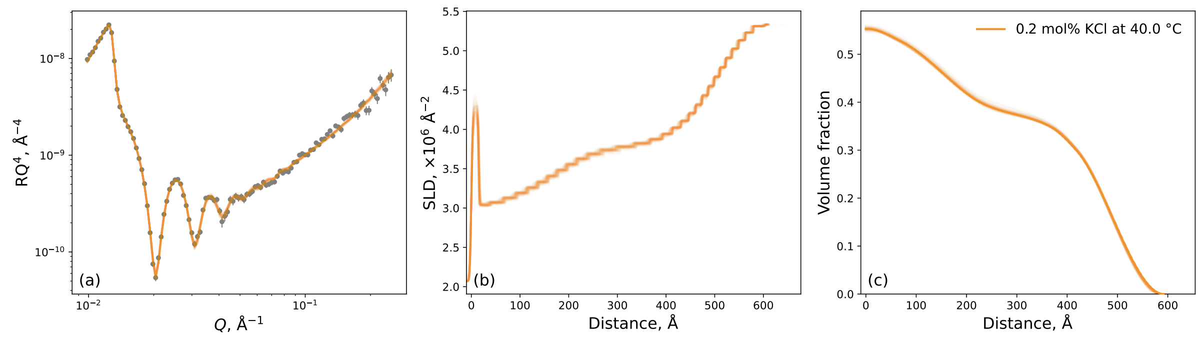

Figure S4.3.1xxiv: (a) Reflectivity, (b) SLD and (c) polymer VF profiles of the 244 Å PNIPAM brush in a 0.2 mol% KCl electrolyte in $x_{\mathrm{D}} = 0.70$ at 40.0 °C with the superimposed distribution of fits from PT-MCMC sampling.

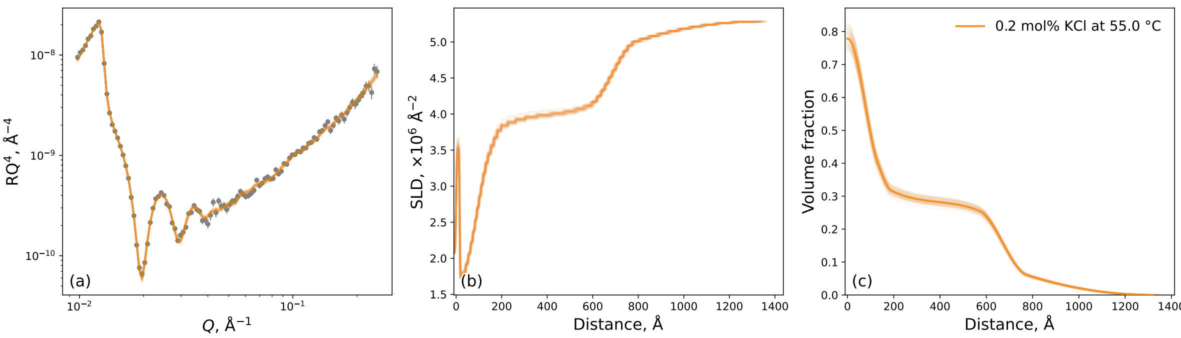

Figure S4.3.1xxv: (a) Reflectivity, (b) SLD and (c) polymer VF profiles of the 244 Å PNIPAM brush in a 0.2 mol% KCl electrolyte in $x_\mathrm{D} = 0.70$ at 55.0 °C with the superimposed distribution of fits from PT-MCMC sampling.

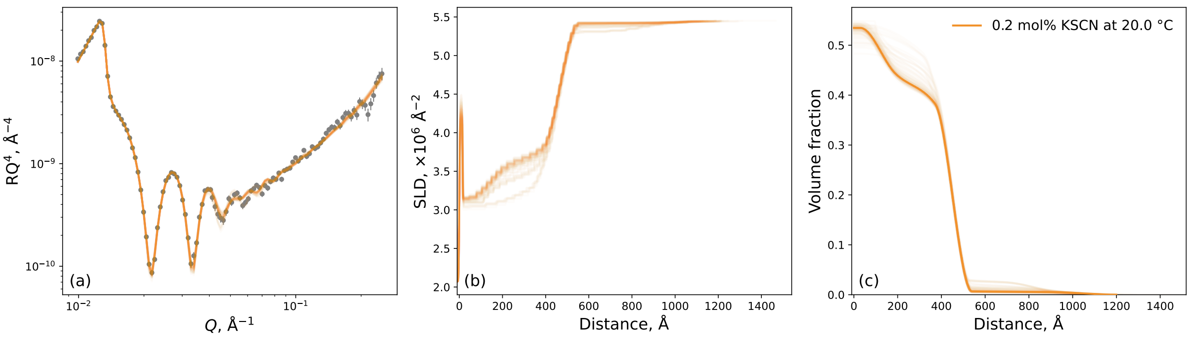

Figure S4.3.1xxvi: (a) Reflectivity, (b) SLD and (c) polymer VF profiles of the 244 Å PNIPAM brush in a 0.2 mol% KSCN electrolyte in $x_\mathrm{D} = 0.70$ at 20.0 °C with the superimposed distribution of fits from PT-MCMC sampling.

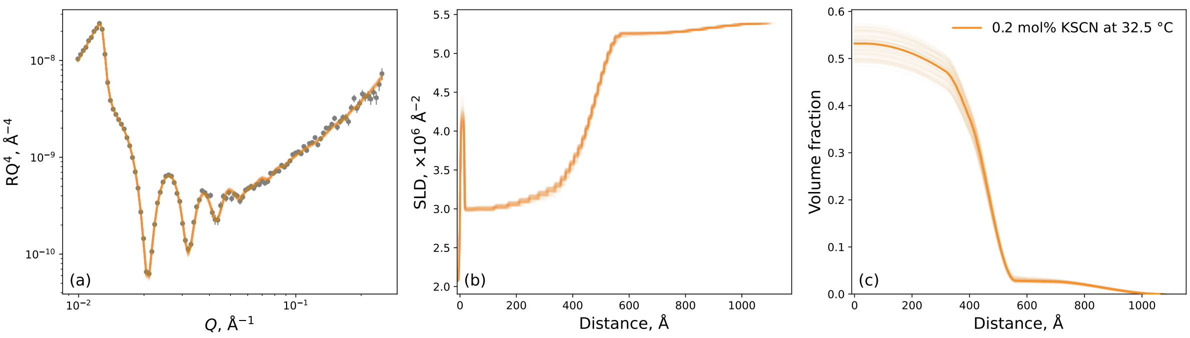

Figure S4.3.1xxvii: (a) Reflectivity, (b) SLD and (c) polymer VF profiles of the 244 Å PNIPAM brush in a 0.2 mol% KSCN electrolyte in $x_\mathrm{D} = 0.70$ at 32.5 °C with the superimposed distribution of fits from PT-MCMC sampling.

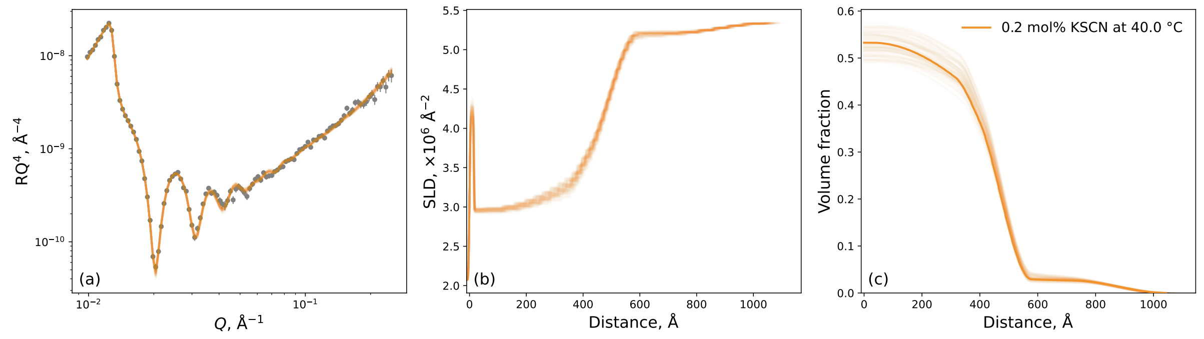

Figure S4.3.1xxviii: (a) Reflectivity, (b) SLD and (c) polymer VF profiles of the 244 Å PNIPAM brush in a 0.2 mol% KSCN electrolyte in $x_D = 0.70$ at 40.0 °C with the superimposed distribution of fits from PT-MCMC sampling.

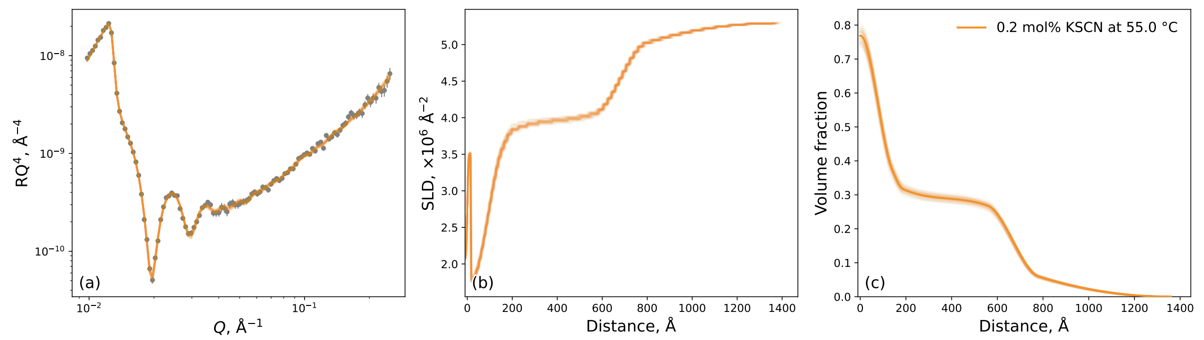

Figure S4.3.1xxix: (a) Reflectivity, (b) SLD and (c) polymer VF profiles of the 244 Å PNIPAM brush in a 0.2 mol% KSCN electrolyte in $x_D = 0.70$ at 55.0 °C with the superimposed distribution of fits from PT-MCMC sampling.

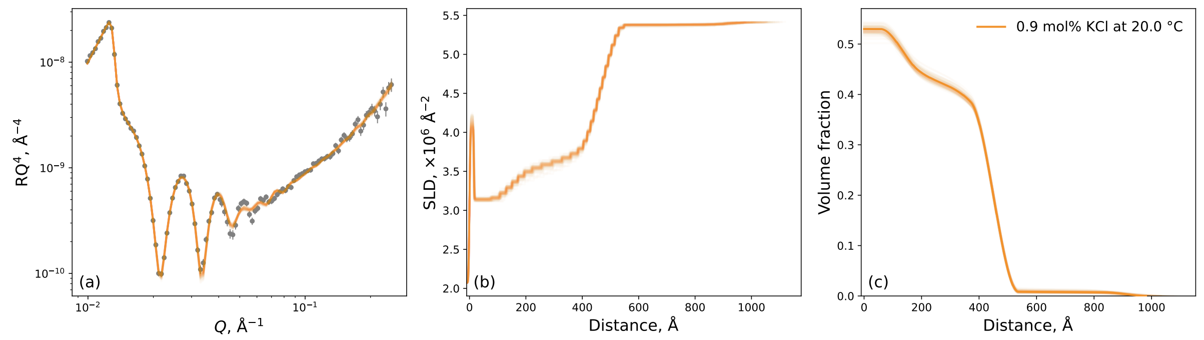

Figure S4.3.1xxx: (a) Reflectivity, (b) SLD and (c) polymer VF profiles of the 244 Å PNIPAM brush in a 0.9 mol% KCl electrolyte in $x_D = 0.70$ at 20.0 °C with the superimposed distribution of fits from PT-MCMC sampling.

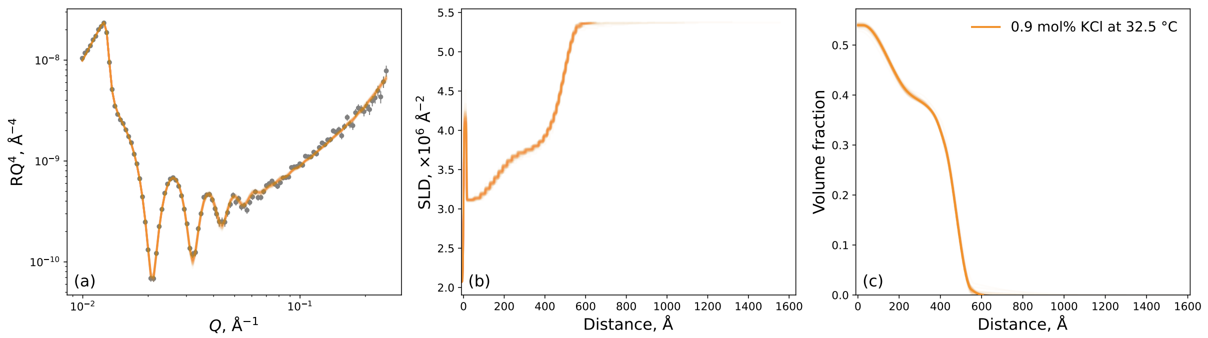

Figure S4.3.1xxxi: (a) Reflectivity, (b) SLD and (c) polymer VF profiles of the 244 Å PNIPAM brush in a 0.9 mol% KCl electrolyte in $x_D = 0.70$ at 32.5 °C with the superimposed distribution of fits from PT-MCMC sampling.

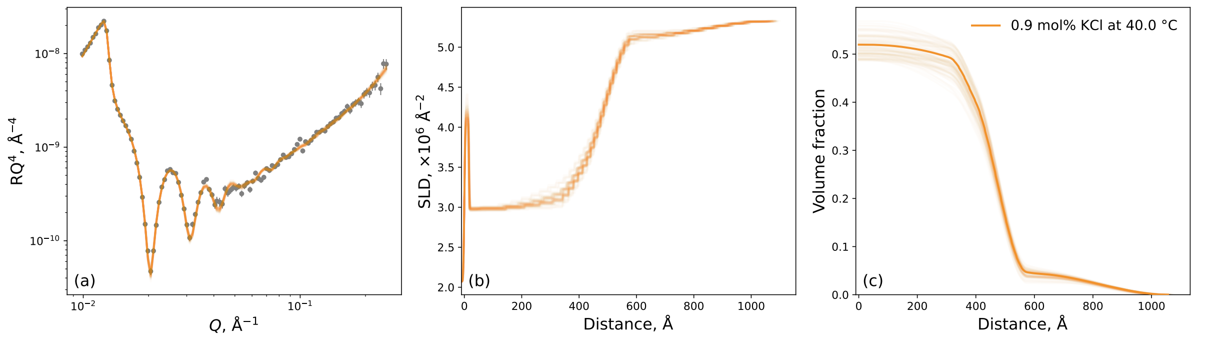

Figure S4.3.1xxxii: (a) Reflectivity, (b) SLD and (c) polymer VF profiles of the 244 Å PNIPAM brush in a 0.9 mol% KCl electrolyte in $x_D = 0.70$ at 40.0 °C with the superimposed distribution of fits from PT-MCMC sampling.

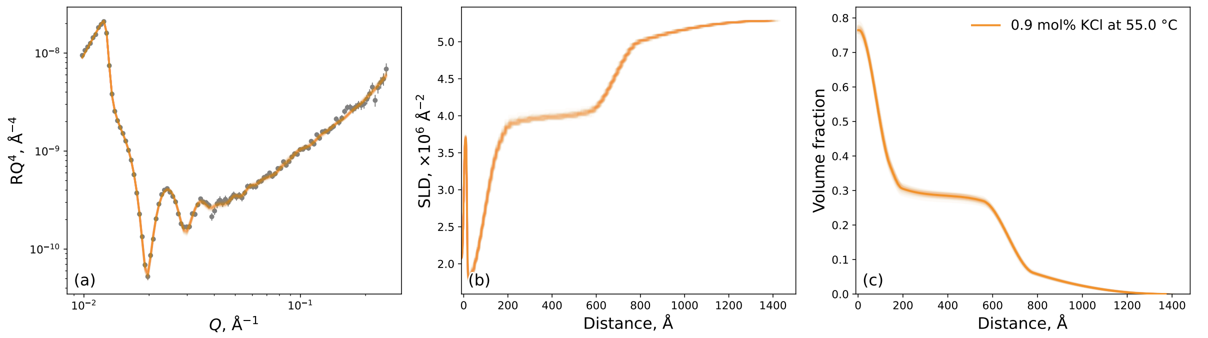

Figure S4.3.1xxxiii: (a) Reflectivity, (b) SLD and (c) polymer VF profiles of the 244 Å PNIPAM brush in a 0.9 mol% KCl electrolyte in $x_D = 0.70$ at 55.0 °C with the superimposed distribution of fits from PT-MCMC sampling.

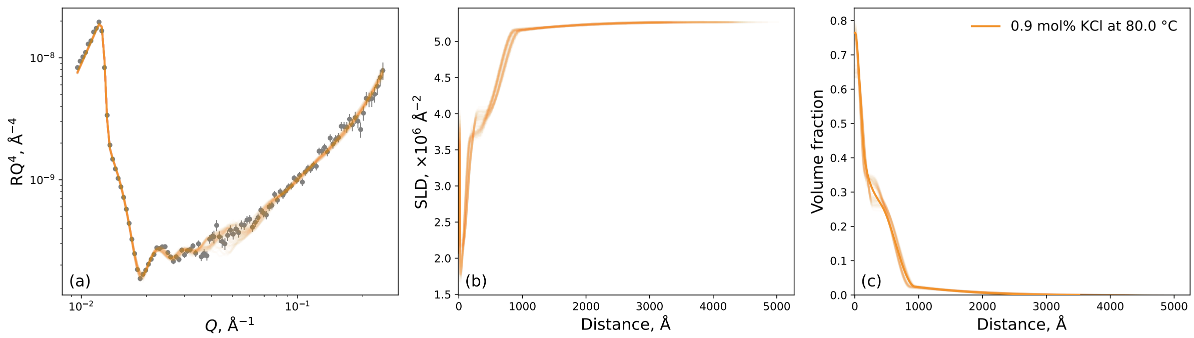

Figure S4.3.1xxxiv: (a) Reflectivity, (b) SLD and (c) polymer VF profiles of the 244 Å PNIPAM brush in a 0.9 mol% KCl electrolyte in $x_{\mathrm{D}} = 0.70$ at 80.0 °C with the superimposed distribution of fits from PT-MCMC sampling.

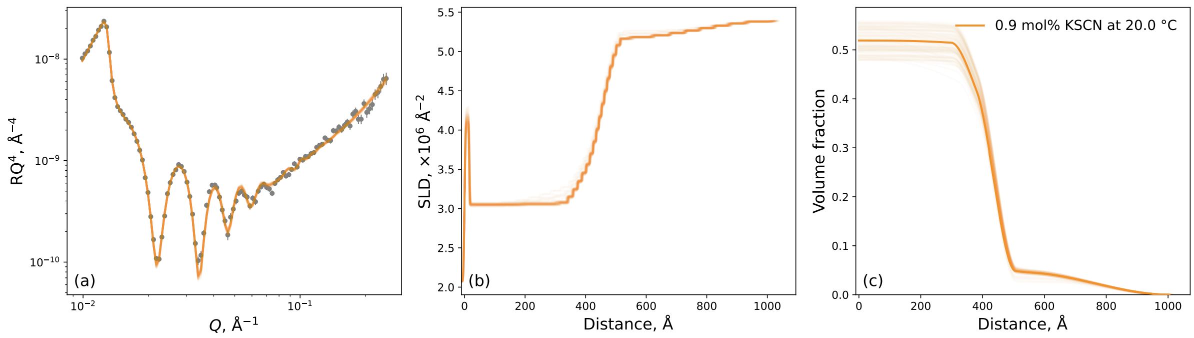

Figure S4.3.1xxxv: (a) Reflectivity, (b) SLD and (c) polymer VF profiles of the 244 Å PNIPAM brush in a 0.9 mol% KSCN electrolyte in $x_{\mathrm{D}} = 0.70$ at 20.0 °C with the superimposed distribution of fits from PT-MCMC sampling.

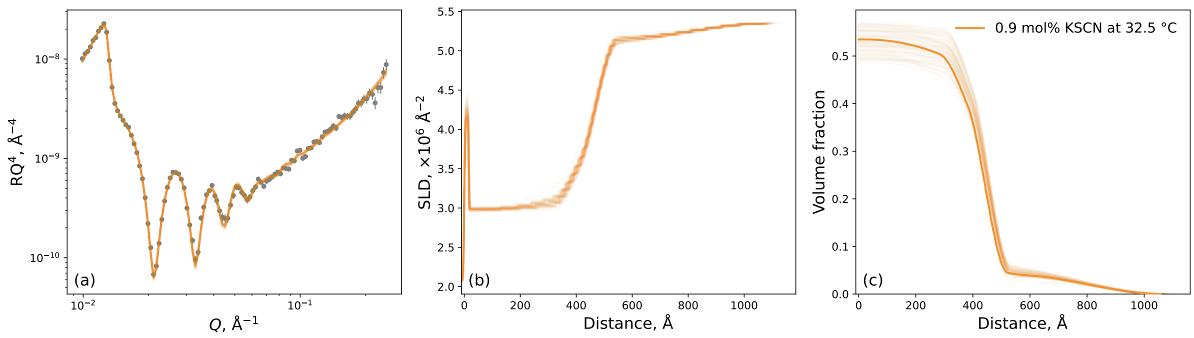

Figure S4.3.1xxxvi: (a) Reflectivity, (b) SLD and (c) polymer VF profiles of the 244 Å PNIPAM brush in a 0.9 mol% KSCN electrolyte in $x_{\mathrm{D}} = 0.70$ at 32.5 °C with the superimposed distribution of fits from PT-MCMC sampling.

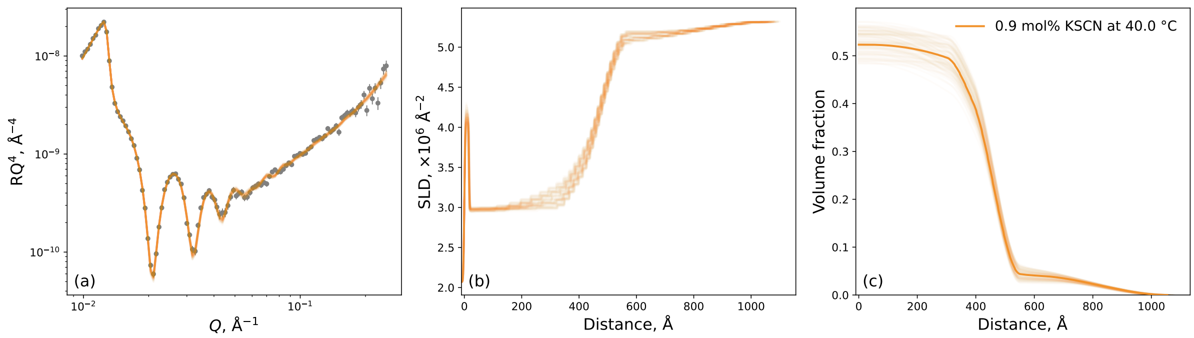

Figure S4.3.1xxxvii: (a) Reflectivity, (b) SLD and (c) polymer VF profiles of the 244 Å PNIPAM brush in a 0.9 mol% KSCN electrolyte in $x_{\mathrm{D}} = 0.70$ at 40.0 °C with the superimposed distribution of fits from PT-MCMC sampling.

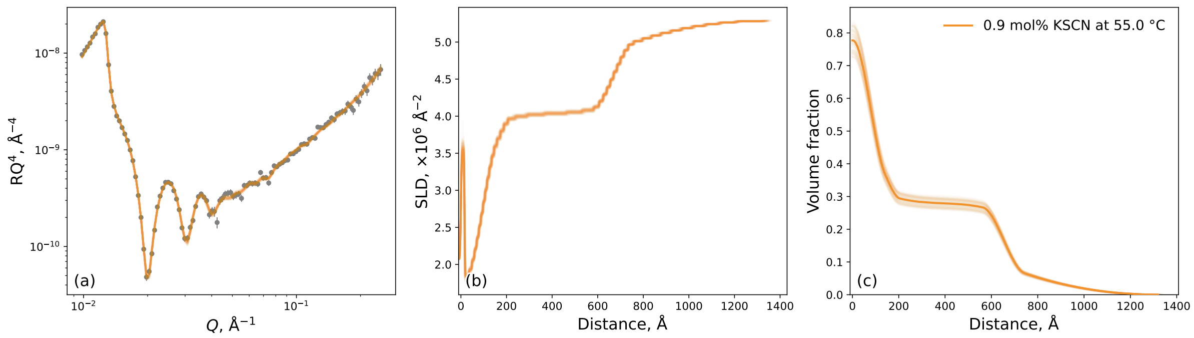

Figure S4.3.1xxxviii: (a) Reflectivity, (b) SLD and (c) polymer VF profiles of the 244 Å PNIPAM brush in a 0.9 mol% KSCN electrolyte in $x_{\mathrm{D}} = 0.70$ at 55.0 °C with the superimposed distribution of fits from PT-MCMC sampling.

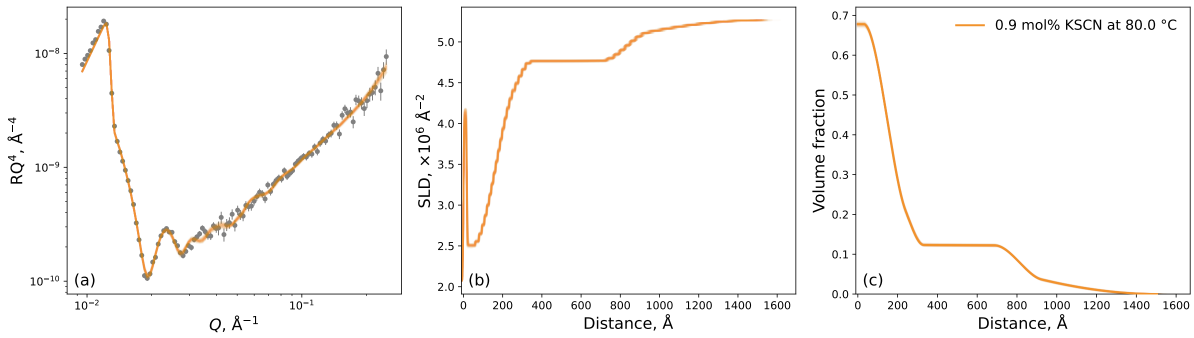

Figure S4.3.1xxxix: (a) Reflectivity, (b) SLD and (c) polymer VF profiles of the 244 Å PNIPAM brush in a 0.9 mol% KSCN electrolyte in $x_{\mathrm{D}} = 0.70$ at 80.0 °C with the superimposed distribution of fits from PT-MCMC sampling.

4.4 100 mol% DMSO

4.4.1 198 Å PNIPAM brush

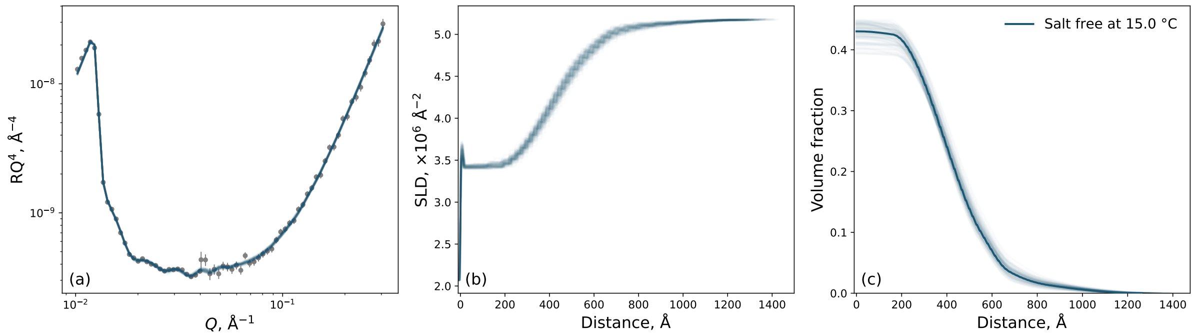

Figure S4.4.1i: (a) Reflectivity, (b) SLD and (c) polymer VF profiles of the 198 Å PNIPAM brush in $x_{\mathrm{D}} = 1.0$ at 15.0 °C with the superimposed distribution of fits from PT-MCMC sampling.

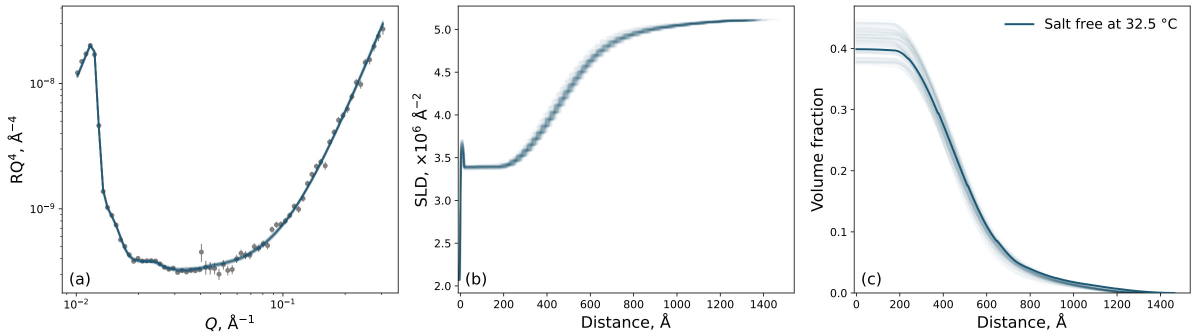

Figure S4.4.1ii: (a) Reflectivity, (b) SLD and (c) polymer VF profiles of the 198 Å PNIPAM brush in $x_{\mathrm{D}} = 1.0$ at 32.5 °C with the superimposed distribution of fits from PT-MCMC sampling.

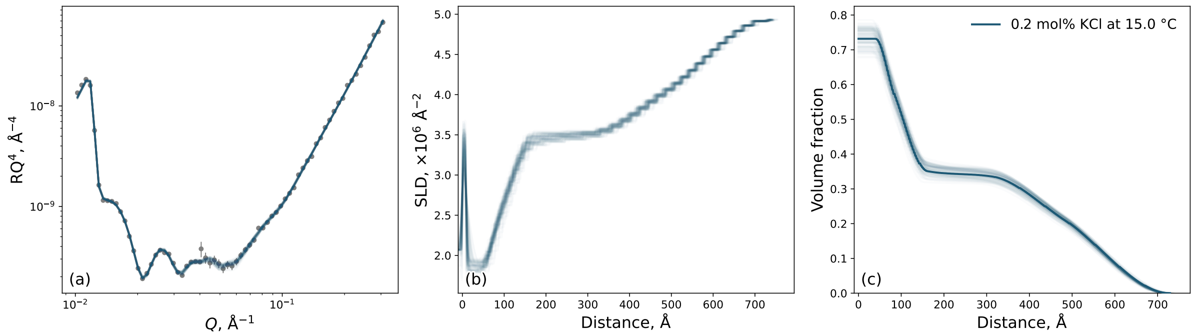

Figure S4.4.1iii: (a) Reflectivity, (b) SLD and (c) polymer VF profiles of the 198 Å PNIPAM brush in a 0.2 mol% KCl electrolyte in $x_{\mathrm{D}} = 1.0$ at 15.0 °C with the superimposed distribution of fits from PT-MCMC sampling.

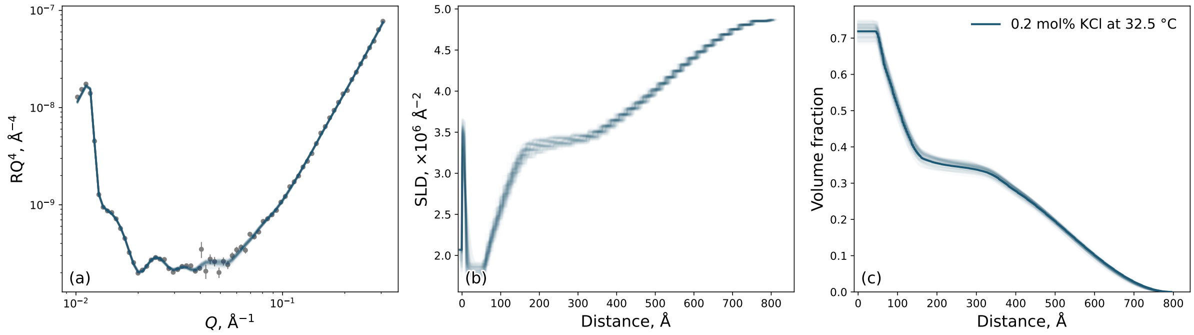

Figure S4.4.1iv: (a) Reflectivity, (b) SLD and (c) polymer VF profiles of the 198 Å PNIPAM brush in a 0.2 mol% KCl electrolyte in $x_{\mathrm{D}} = 1.0$ at 32.5 °C with the superimposed distribution of fits from PT-MCMC sampling.

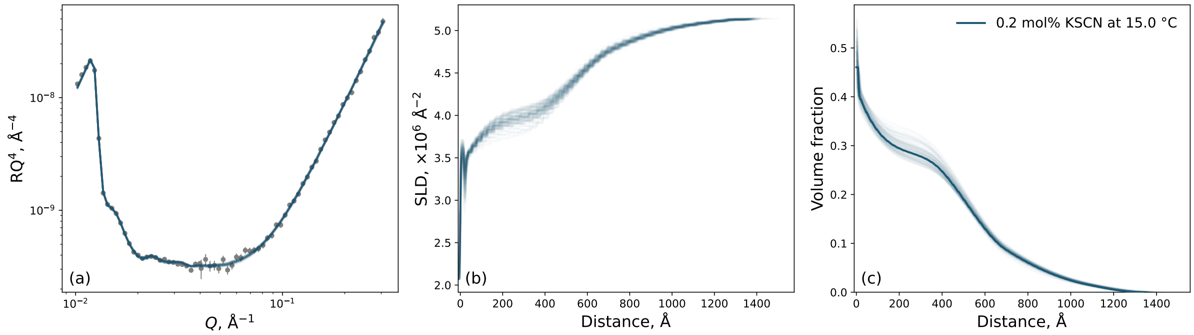

Figure S4.4.1v: (a) Reflectivity, (b) SLD and (c) polymer VF profiles of the 198 Å PNIPAM brush in a 0.2 mol% KSCN electrolyte in $x_{\mathrm{D}} = 1.0$ at 15.0 °C with the superimposed distribution of fits from PT-MCMC sampling.

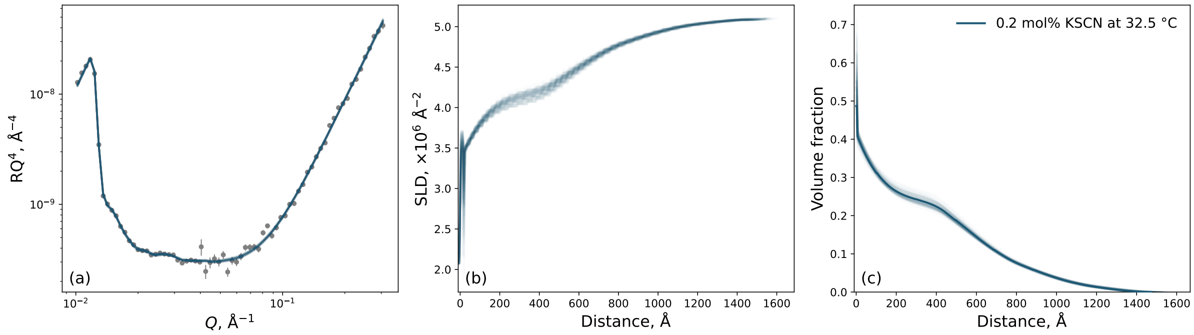

Figure S4.4.1vi: (a) Reflectivity, (b) SLD and (c) polymer VF profiles of the 198 Å PNIPAM brush in a 0.2 mol% KSCN electrolyte in $x_{\mathrm{D}} = 1.0$ at 32.5 °C with the superimposed distribution of fits from PT-MCMC sampling.

References

[1] K. P. Gregory, E. J. Wanless, G. B. Webber, V. S. Craig and A. J. Page, *Chemical Science*, 2021, **12**, 15007–15015.

[2] H. Robertson, I. J. Gresham, S. W. Prescott, G. B. Webber, E. J. Wanless and A. Nelson, *SoftwareX*, 2022, **20**, 101225.

[3] A. R. J. Nelson and S. W. Prescott, *Journal of Applied Crystallography*, 2019, **52**, 193–200.

[4] I. J. Gresham, T. J. Murdoch, E. C. Johnson, H. Robertson, G. B. Webber, E. J. Wanless, S. W. Prescott and A. R. J. Nelson, *Journal of Applied Crystallography*, 2021, **54**, 739–750.
